# Hedgehog signaling regulates cell motility and optic fissure and stalk formation during vertebrate eye morphogenesis

**DOI:** 10.1242/dev.165068

**Published:** 2018-11-19

**Authors:** Hannah B. Gordon, Sarah Lusk, Keith R. Carney, Emily O. Wirick, Brooke Froelich Murray, Kristen M. Kwan

**Affiliations:** Department of Human Genetics, University of Utah, Salt Lake City, UT 84112, USA

**Keywords:** Hedgehog signaling, Coloboma, Eye, Morphogenesis, Ptch2, Zebrafish

## Abstract

Establishment of precise three-dimensional tissue structure is vital for organ function. In the visual system, optic fissure and stalk morphogenesis is a crucial yet poorly understood process, disruptions of which can lead to coloboma, a birth defect causing visual impairment. Here, we use four-dimensional imaging, cell tracking, and molecular genetics in zebrafish to define the cell movements underlying normal optic fissure and stalk formation. We determine how these events are disrupted in a coloboma model in which the Hedgehog (Hh) receptor *ptch2* is lost, resulting in overactive Hh signaling. In the *ptch2* mutant, cells exhibit defective motile behaviors and morphology. Cells that should contribute to the fissure do not arrive at their correct position, and instead contribute to an ectopically large optic stalk. Our results suggest that overactive Hh signaling, through overexpression of downstream transcriptional targets, impairs cell motility underlying optic fissure and stalk formation, via non-cell-autonomous and cell-autonomous mechanisms. More broadly, our cell motility and morphology analyses provide a new framework for studying other coloboma-causing mutations that disrupt optic fissure or stalk formation.

## Abstract (precis)

**Summary:** Multidimensional imaging of *ptch2* mutant zebrafish uncovers a role for the Hh signaling pathway in regulating the cell and tissue dynamics underlying early eye morphogenesis.

## INTRODUCTION

**Fig. 1.**
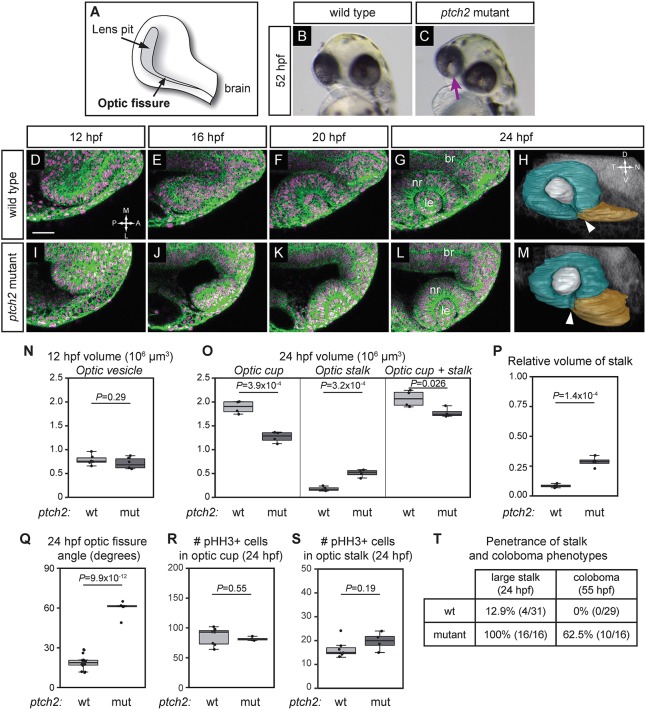
**Optic cup morphogenesis and optic fissure and stalk formation are disrupted in the *ptch2^tc294z^* mutant.** (A) Schematic of optic fissure at optic cup stage, 24 hpf. (B) Wild-type embryo, 52 hpf: the eye is evenly pigmented. (C) *ptch2^tc294z^* mutant embryo, 52 hpf: coloboma is apparent as a region of hypopigmentation in the eye (arrow). (D-G,I-L) Wild-type (D-G) and *ptch2^tc294z^* mutant (I-L) optic cup formation, single confocal slices from four-dimensional imaging data set (12-24 hpf). Dorsal view. Green, EGFP-CAAX (membranes); magenta, H2A.F/Z-mCherry (nuclei). (H,M) Volume rendering of wild-type (H) and *ptch2^tc294z^* mutant (M) embryos, 24 hpf. Lateral view. Teal, optic cup; gray, lens; gold, optic stalk. Arrowhead indicates the optic fissure, which has not formed correctly in the mutant. (N) Optic vesicle volume in wild-type (wt) and *ptch2^tc294z^* mutant (mut) embryos, 12 hpf. *n*=6 for each genotype. (O) Optic cup, stalk, and total volume, 24 hpf. *n*=4 for each genotype. (P) Relative volume of stalk as a proportion of optic cup+stalk volume. *n*=4 for each genotype. (Q) Angle measurement of optic fissure opening, 24 hpf. *n*=16 wt, 5 mut. (R,S) Number of phospho-histone H3-positive cells in the optic cup (R) and stalk (S), 24 hpf. *n*=7 wt, 4 mut. (T) Comparison of penetrance of large stalk and coloboma phenotypes at 24 and 55 hpf, respectively. (N,O,Q,S) Unpaired Student's *t*-test. (P,R) Unpaired Welch's *t*-test to account for unequal variance. br, brain; le, lens; nr, neural retina. RPE is very flattened and difficult to see; thus it is not labeled. Scale bar: 50 µm.

During eye development, cells and tissues must undergo dynamic movements to generate the complex, precise three-dimensional architecture that is crucial for its function. The embryonic structure of the eye is initially established in the process of optic cup morphogenesis: the optic vesicle evaginates as an outpocketing of the neuroepithelium, then elongates and invaginates to generate the optic cup, as the connection between the optic cup and prospective brain narrows to become the optic stalk (Chow and Lang, 2001; Yang, 2004; Adler and Canto-Soler, 2007; Martinez-Morales and Wittbrodt, 2009; Fuhrmann, 2010; Sinn and Wittbrodt, 2013; Bazin-Lopez et al., 2015). This process occurs rapidly in zebrafish, from 10 to 24 h post-fertilization (hpf). During optic cup formation, a cleft structure, known as the optic fissure, appears at the ventral side of the optic cup and optic stalk (Fig. 1A). Subsequently, the optic fissure closes along its entire length in the optic cup and stalk: the margins of the fissure fuse to generate the seamless conduit through which retinal vasculature enters the eye and retinal ganglion cell axons exit. Significant work has focused on the later stages of optic fissure development, with an emphasis on optic fissure fusion (24-48 hpf in zebrafish), including genetic and descriptive studies in multiple organisms (Hero, 1989, 1990; Hero et al., 1991; James et al., 2016; Bernstein et al., 2018). Yet the early stages of optic cup morphogenesis that generate the optic fissure and optic stalk remain a mystery: little is known of the cellular dynamics driving their initial formation.

The molecular mechanisms regulating optic fissure and stalk formation are also poorly understood, yet disruptions to the development of these crucial structures manifest as the birth defect uveal coloboma, a significant cause of pediatric blindness (Gregory-Evans et al., 2004; Fitzpatrick and van Heyningen, 2005). The genetic architecture underlying coloboma is heterogeneous; human genetic studies have uncovered numerous putative causative mutations (Gregory-Evans et al., 2004; Williamson and FitzPatrick, 2014; Patel and Sowden, 2017). Work in model organisms (mouse, chick, zebrafish), has begun to examine these genes during eye development, also identifying crucial molecules, signaling pathways and extraocular cell populations. For example, human mutations in *PAX6*, *PITX2* and *SOX11* can all result in coloboma, and animal models have uncovered transcriptional network interactions (Gage et al., 1999; Ozeki et al., 1999; Stull and Wikler, 2000; Baulmann et al., 2002; Singh et al., 2002; Azuma et al., 2003; Gregory-Evans et al., 2004; Pillai-Kastoori et al., 2014). Signaling molecules such as Gdf6, Lrp6 and retinoic acid have also been implicated through a combination of human and model organism genetics (Asai-Coakwell et al., 2007; Zhou et al., 2008; Lupo et al., 2011; French et al., 2013). Yet even as genetic models and a growing coloboma gene network continue to emerge, an understanding of how these mutations disrupt the actual underlying morphogenetic events remains elusive.

One pathway vital to optic fissure development is the Hedgehog (Hh) signaling pathway: mutations upstream, within and downstream of Hh signaling can induce coloboma in humans and model organisms (Gregory-Evans et al., 2004). For example, upstream of Hh signaling, mutations in Sox genes disrupt optic fissure development in zebrafish by altering Hh ligand expression (Pillai-Kastoori et al., 2014; Wen et al., 2015). Additionally, SHH itself can be mutated in human coloboma (Schimmenti et al., 2003). The downstream transcriptional target *PAX2* is mutated in human renal-coloboma syndrome and has been studied in mouse and zebrafish (Keller et al., 1994; Sanyanusin et al., 1995; Favor et al., 1996; Torres et al., 1996; Macdonald et al., 1997; Eccles and Schimmenti, 1999).

The Hh receptor *ptch2* is also associated with coloboma. Human mutations in *PTCH* result in Gorlin syndrome (Hahn et al., 1996; Smyth et al., 1999); affected individuals can present with coloboma (Ragge et al., 2005). Ptch2 is a negative-feedback regulator: its expression is induced as a downstream transcriptional target of Hh signal transduction, and the protein inhibits signaling via the transmembrane molecule Smoothened. Therefore, loss-of-function mutations in *ptch2* result in overactive Hh signaling specifically within cells responding to Hh ligand. In zebrafish, the loss-of-function *ptch2^tc294z^* mutant (Lee et al., 2008) exhibits coloboma (Fig. 1B,C). Rescue experiments using the Hh signaling inhibitor cyclopamine demonstrated that coloboma is caused by overactive Hh signaling (Lee et al., 2008); however, the cellular and molecular mechanisms by which this disrupts optic fissure development remain unknown.

Optic fissure morphogenesis, a multi-stage process including formation and fusion, could potentially be disrupted at any step to result in coloboma. Additionally, the optic stalk, through which the optic fissure extends, is itself a poorly understood structure that is crucial for the visual system. Here, we set out to directly visualize and determine the cellular events underlying the initial step of optic fissure and stalk formation. What cell movements are involved? How is this disrupted in a specific coloboma model of overactive Hh signaling? Defining the basic cellular processes provides a framework to begin to understand how these structures form and develop. Furthermore, this will lay the groundwork for dissecting additional coloboma-causing mutations and establishing the spectrum of cellular events that are sensitive to genetic perturbations.

Here, using a combination of four-dimensional microscopy, computational methods and molecular genetics, we define the cell movements underlying normal optic fissure and stalk formation; determine the morphogenetic defects in the *ptch2^tc294z^* mutant, in which optic fissure and stalk formation are disrupted; and examine the molecular basis by which overactive Hh signaling causes these defects.

## RESULTS

### Optic cup morphogenesis and optic fissure and stalk formation are disrupted in the *ptch2^tc294z^* mutant

The cellular events underlying normal optic fissure and stalk formation are not yet known. To determine the cellular basis of their formation, we acquired four-dimensional time-lapse imaging datasets of optic cup morphogenesis in wild-type embryos, spanning 12-24 hpf. Embryos were labeled for membranes (EGFP-CAAX) and chromatin (H2A.F/Z-mCherry). In wild-type embryos, tissue rearrangements are executed (Fig. 1D-G; Movie 1), many of which have been described by multiple groups (Martinez-Morales and Wittbrodt, 2009; Picker et al., 2009; Kwan et al., 2012; Heermann et al., 2015; Sidhaye and Norden, 2017). These tissue rearrangements lead to the formation of the optic cup at 24 hpf, comprising neural retina, retinal pigmented epithelium (RPE), and lens. At this time point, we can visualize the three-dimensional optic cup structure by manually segmenting the neural retina and RPE (teal), lens (gray) and optic stalk (gold) (Fig. 1H). The optic fissure is apparent as a cleft structure with two closely apposed margins (temporal and nasal) at the ventral side of the optic cup (Fig. 1H, arrowhead; Movie 2).

The loss-of-function *ptch2^tc294z^* mutant displays coloboma, a structural defect in the eye caused by aberrant development of the optic fissure (Lee et al., 2008). Although the morphological features of the phenotype have been described in detail at stages after optic cup morphogenesis, the precise timing of the underlying cellular defects is unknown. We first investigated whether optic fissure and stalk formation might be disrupted in the *ptch2^tc294z^* mutant, by performing four-dimensional imaging of optic cup morphogenesis. In the *ptch2^tc294z^* mutant, we observe no gross defect in optic vesicle formation: the volumes of mutant and wild-type optic vesicles are not significantly different (Fig. 1N; wt 0.79±0.1×10^6^ µm^3^, mutant 0.71±0.1×10^6^ µm^3^). As optic cup morphogenesis progresses, the eye appears (in single optical sections) to form normally (Fig. 1I-L; Movie 3), with neural retina and RPE enwrapping the lens. However, three-dimensional visualization of the optic cup reveals defects in the ventral portion of the eye and optic stalk. Whereas the wild-type optic cup contains closely apposed optic fissure margins (Fig. 1H, arrowhead), the *ptch2^tc294z^* mutant optic fissure does not (Fig. 1M, arrowhead; Movie 4). Quantification reveals that the optic fissure opening is significantly larger in *ptch2^tc294z^* mutant embryos (Fig. 1Q; wt 19±5.25°, mutant 59.6±6.2°). In addition to this structural defect, the mutant optic cup is smaller than the wild-type optic cup (Fig. 1O; wt 1.89±0.13×10^6^ µm^3^, mutant 1.27±0.11×10^6^ µm^3^), and the mutant optic stalk is significantly larger than the wild-type optic stalk (Fig. 1O; wt 0.18±0.05×10^6^ µm^3^, mutant 0.51±0.08×10^6^ µm^3^). The total volume of the mutant optic cup and stalk is significantly smaller than that of wild type (Fig. 1O; wt 2.07±0.17×10^6^ µm^3^, mutant 1.77±0.1×10^6^ µm^3^); yet, despite the differences in total volume, the optic stalk is still a larger relative volume (Fig. 1P; wt 0.085±0.016, mutant 0.287±0.045). The appearance of this volume defect suggests that some aspect of optic cup morphogenesis is disrupted in the *ptch2^tc294z^* mutant.

In other contexts, Hh signaling can drive cell proliferation (Evangelista et al., 2006), and hyperproliferation can be sufficient to cause coloboma (Kim et al., 2007). However, *ptch2* mutants were not previously found to exhibit increased proliferation, at stages subsequent to optic cup formation (Lee et al., 2008). Because we observed morphological defects prior to when proliferation had been previously assayed, we tested whether mitosis might be altered earlier (24 hpf). We find no significant difference in the number of phospho-histone-H3-positive cells in the optic cup or stalk in *ptch2^tc294z^* mutants (Fig. 1R,S; wt optic cup 85±15, mutant optic cup 82±3; wt stalk 16±4, mutant stalk 20±4). Therefore, changes in proliferation are less likely to account for the difference in eye volume observed in the *ptch2^tc294z^* mutants; instead, these defects may be due to altered morphogenetic movements.

Finally, the coloboma phenotype in *ptch2^tc294z^* mutants is incompletely penetrant at 2 days post-fertilization (Lee et al., 2008), so we assayed penetrance of the 24 hpf phenotype, in particular the large optic stalk and its relationship to the appearance of coloboma. Embryos were imaged at 24 hpf, then scored blindly for coloboma at 55 hpf. We find that the optic stalk phenotype at 24 hpf is fully penetrant, whereas coloboma is incompletely penetrant; all embryos with coloboma at 55 hpf previously exhibited a large optic stalk (Fig. 1T). We conclude from these data that optic cup morphogenesis, optic stalk formation and optic fissure formation are defective in *ptch2^tc294z^* mutants, and that optic fissure and stalk defects are apparent prior to optic fissure fusion. Thus, we focused on how these phenotypes originate, at this early stage of development.

### Cell movements underlying optic fissure and stalk formation are disrupted in the *ptch2^tc294z^* mutant

Because the *ptch2^tc294z^* mutant displayed defective optic fissure and stalk formation, we sought to identify the specific cell movements disrupted during this process, using two independent assays.

**Fig. 2.**
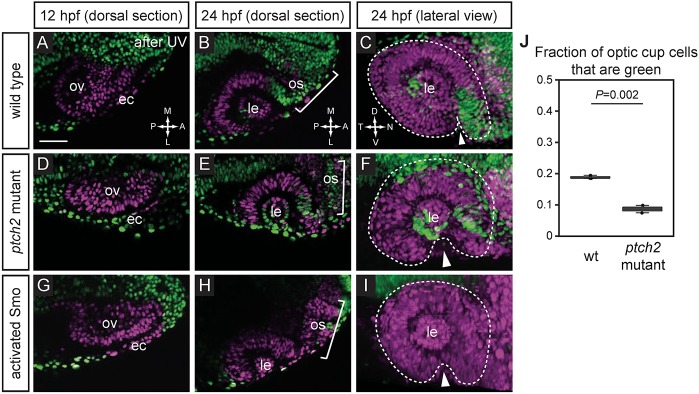
**Extended evagination cell movements are disrupted by overactive Hh signaling.** (A-I) Wild-type (A-C), *ptch2^tc294z^* mutant (D-F) and activated Smo RNA-injected (G-I) embryos subjected to nls-Kaede photoconversion at the optic vesicle stage (A,D,G). The entire optic vesicle was converted from green to red (magenta) fluorescence (along with some ectoderm). (B,E,H) Single confocal sections of optic cups from photoconverted embryos, 24 hpf, dorsal view. (C,F,I) 3D-rendered optic cups, 24 hpf, lateral view. Dashed lines outline the optic cup. Arrowheads indicate the optic fissure. (J) Quantification of extended evagination movements, indicated by the proportion of green nuclei in the optic cup (green nuclei divided by the total number of nuclei) in wild-type (wt) and *ptch2^tc294z^* mutant embryos. The proportion of green nuclei in the optic cup is significantly reduced in *ptch2^tc294z^* mutants. *n*=3 wt, 2 mut. Unpaired Student's *t*-test. ec, ectoderm; le, lens; os, optic stalk; ov, optic vesicle. Scale bar: 50 µm.

First, we utilized a previously developed photoactivation-based fate-mapping assay using nls-Kaede, a nuclear-localized version of the photoconvertible fluorophore Kaede (Ando et al., 2002; Kwan et al., 2012). This assay allows us to visualize the last cells to move into the optic vesicle during evagination, during a period we termed extended evagination (Kwan et al., 2012). These cells contribute directly to the nasal margin of the optic fissure in wild-type embryos (Kwan et al., 2012). At 12 hpf, we photoconverted the entire optic vesicle (and some overlying ectoderm) to red fluorescence (pseudocolored magenta) (Fig. 2A). At 24 hpf, we visualize extended evagination as green nuclei within the optic cup, as these cells have moved out of the midline region and into the eye after 12 hpf. In wild-type embryos, green nuclei are found in the nasal margin of the optic fissure, the ventronasal retina and the optic stalk (Fig. 2B,C). Green nuclei in the lens are derived from ectodermal cells not photoconverted. In the *ptch2^tc294z^* mutant, these late-moving cells appear to be excluded from the defective optic fissure (Fig. 2D-F). Green nuclei are still found in the nasal retina (dorsal to the optic fissure) and optic stalk, indicating that some cell movement occurred, but not into the optic fissure. The temporal margin remains devoid of green nuclei. We quantified the proportion of green cells within the optic cup under wild-type and *ptch2*^tc294z^ mutant conditions, using a custom MATLAB-based three-dimensional cell counting script Abacus (see Materials and Methods and supplementary Materials and Methods). The *ptch2^tc294z^* mutant optic cup contains a significantly smaller proportion of green nuclei in the optic cup (Fig. 2J; wt 18.9±0.5%, mutant 8.7±1.7%), indicating impaired extended evagination cell movements. As an independent method to overactivate Hh signaling, we injected RNA encoding an activated form of the Smoothened (Smo) transmembrane protein (rSmoM2-EGFP; Huang and Schier, 2009). Expression of activated Smo also impairs extended evagination cell movements (Fig. 2G-I): no green nuclei are found in the optic cup or optic stalk, a more severe phenotype than observed in the *ptch2*^tc294z^ mutant. Consistent with this, the optic fissure opening is also more severely affected by activated Smo (wt 19±5.3°; *ptch2*^tc294z^ mutant 59.6±6.2°; activated Smo 88.4±13.4°; data not shown, Fig. 1Q). This could reflect functional differences in the manipulations: *ptch2* is a negative-feedback regulator, loss of which leads to overactive Hh signaling, but only within cells responding to Hh ligand. In contrast, activated Smo RNA injection induces overactive Hh signaling in all cells, even those not responding to Hh ligand. Yet both independent methods of overactivating Hh signaling disrupt cell movements contributing to the optic fissure.

**Fig. 3.**
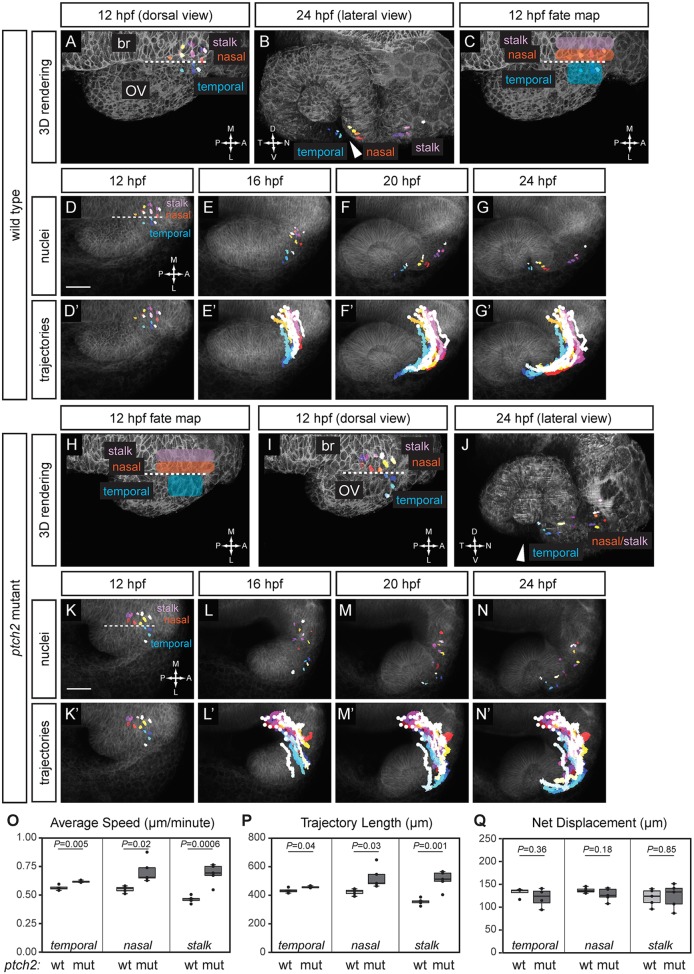
**Origins and trajectories of cells contributing to the optic fissure are disrupted in the *ptch2^tc294z^* mutant.** (A-G′) Wild-type optic fissure and stalk cell movements (12-24 hpf). (A,B) Rendering of nuclei and membrane channel, at 12 hpf, dorsal view (A) and 24 hpf, lateral view (B). (C) Fate map of optic fissure and stalk cells. (D-G) Nuclei over membrane channel average projection. (D′-G′) Trajectories over membrane channel average projection. (H-N′) *ptch2^tc294z^* mutant optic fissure cell movements (12-24 hpf). (H) Application of wild-type fate map to *ptch2^tc294z^* mutant optic vesicle. (I,J) Rendering of selected nuclei and membrane channel, at 12 hpf, dorsal view (I) and 24 hpf, lateral view (J). Arrowheads in B and J indicate the optic fissure opening. (K-N) Nuclei over membrane channel average projection. (K′-N′) Trajectories over membrane channel average projection. (O-Q) Quantification of cell-tracking data in wild-type (wt) and *ptch2^tc294z^* mutant (mut) embryos. *n*=4 temporal cells, 5 nasal cells, and 5 stalk cells per genotype. (O) Three-dimensional average speed. (P) Three-dimensional trajectory length. (Q) Three-dimensional net displacement. White circles indicate trajectory origins. Blue shades, temporal nuclei; red/yellow shades, nasal nuclei; purple shades, stalk nuclei. Dashed line indicates the boundary between the optic vesicle and the midline region. br, brain; OV, optic vesicle. Scale bars: 50 µm.

These fate-mapping experiments provide initial indications of defects in the *ptch2*^tc294z^ mutant. In order to identify precisely when and where morphogenetic movements are disrupted, we utilized a second assay to examine cell movements: four-dimensional cell tracking using our MATLAB-based manual cell-tracking software, LongTracker (Kwan et al., 2012). Because the cellular basis of normal optic fissure and stalk formation is unknown, we first mapped the origins and trajectories of cells contributing to the optic fissure margins in the optic cup and the optic stalk. LongTracker allows us to carry out retrospective cell tracking; therefore, we selected cells in the optic fissure margins (nasal and temporal) and optic stalk at optic cup stage (Fig. 3B). We tracked the movement of these nuclei backwards in four dimensions to determine their origins at optic vesicle stage (12 hpf). In wild-type embryos, cells contributing to the optic fissure margins and stalk arise from adjacent domains at optic vesicle stage (Fig. 3A). There is a key spatial difference: the nasal margin (red/yellow shades) and optic stalk cells (purple shades) are found within the midline domain, whereas the temporal margin cells (blue shades) are found within the optic vesicle. This is consistent with our nls-Kaede results: nuclei contributing to the temporal margin of the optic fissure are converted to red/magenta fluorescence at 12 hpf, whereas nuclei contributing to the nasal margin and optic stalk, arising from the midline region, remain green. Having mapped the origins of these cells, we visualized trajectories of prospective optic fissure and optic stalk cells. All three groups of cells move along a ‘J-shaped’ trajectory, with nasal margin cells moving into the optic vesicle and following temporal margin cells. Optic stalk cells subsequently follow nasal margin cells (Fig. 3D-G′; Movies 5,6).

With these wild-type data in hand, we investigated when and where optic fissure and stalk cell movements are affected in the *ptch2^tc294z^* mutant. Using the wild-type data, we first generated a fate map of our regions of interest (Fig. 3C; temporal margin, nasal margin and optic stalk), and applied it to the *ptch2^tc294z^* mutant data set (Fig. 3H,I), reasoning that forward cell tracking from those domains would allow us to pinpoint defective cell movements. We find that cells arising from wild-type optic stalk, nasal and temporal domains at optic vesicle stage do not achieve their correct positions in the *ptch2^tc294z^* mutant optic cup (Fig. 3I,J). Cells that originate from the prospective temporal domain instead contribute to the nasal side of the disrupted *ptch2^tc294z^* mutant optic fissure; cells from nasal and stalk origins are intermingled within the stalk; and these positions are shifted proximally toward the midline relative to wild type. To determine whether the final positions of these cells were incorrect due to aberrant trajectories, we visualized their movements: mutant cells travel along a shallow J-shaped trajectory, with cells from the prospective temporal region (blue shades) followed by cells from the prospective nasal and stalk regions (Fig. 3K-N′; Movie 7,8; red/yellow shades and purple shades). Therefore, trajectory shape is not grossly changed.

Although the mutant trajectory shape appears superficially normal, we quantified individual cell movements (all in three dimensions) for average speed, trajectory length and net displacement. In the *ptch2^tc294z^* mutant, cells from each domain move significantly faster than their wild-type counterparts (Fig. 3O; wt temporal 0.56±0.02 µm/min, mutant temporal 0.62±0.01 µm/min; wt nasal 0.55±0.03 µm/min, mutant nasal 0.71±0.1 µm/min; wt stalk 0.46±0.03 µm/min, mutant stalk 0.69±0.09 µm/min). Mutant cells also execute a significantly longer total three-dimensional trajectory length (Fig. 3P; wt temporal 432.8±17.6 µm, mutant temporal 457.3±6.7 µm; wt nasal 422.9±20.5 µm, mutant nasal 524.9±75.8 µm; wt stalk 355.5±23.1 µm, mutant stalk 507.2±64.4 µm). However, three-dimensional net displacement is not significantly different in any domain (Fig. 3Q; wt temporal 132.4±10.3 µm, mutant temporal 120.8±20.8 µm; wt nasal 137.1±5.7 µm, mutant nasal 127.4±13.8 µm; wt stalk 121.2±18.3 µm, mutant stalk 124±26.5 µm). We hypothesize that processive cell motility may be affected in the *ptch2^tc294z^* mutant optic fissure and stalk: cells move faster and over a longer total path, but do not achieve the same destination as their wild-type counterparts.

The tracked cells from the three domains all contributed to the nasal side of the optic fissure and stalk; therefore, we investigated from where the mutant temporal margin arises. We selected nuclei of temporal margin cells from the *ptch2^tc294z^* mutant optic fissure at 24 hpf and tracked them in four dimensions retrospectively to their origin at 12 hpf (Fig. S1A,B). These cells arise from a position which, in wild type, should contribute to temporal retina (Kwan et al., 2012), but they move along a novel trajectory (Fig. S1C-F′; Movies 9,10). These cells may be either directly affected by overactive Hh signaling, or indirectly affected as a secondary consequence of other disrupted cell movements; to resolve these possibilities, we assayed localization of overactive Hh signaling (discussed later in the article).

Taken together, our data indicate that cell movements underlying optic fissure and stalk formation are disrupted in the *ptch2^tc294z^* mutant. No fissure cells achieve their correct final positions and, notably, nasal fissure cells contribute to the stalk, instead of the optic cup.

### Single-cell behaviors and morphology are disrupted in the *ptch2^tc294z^* mutant

**Fig. 4.**
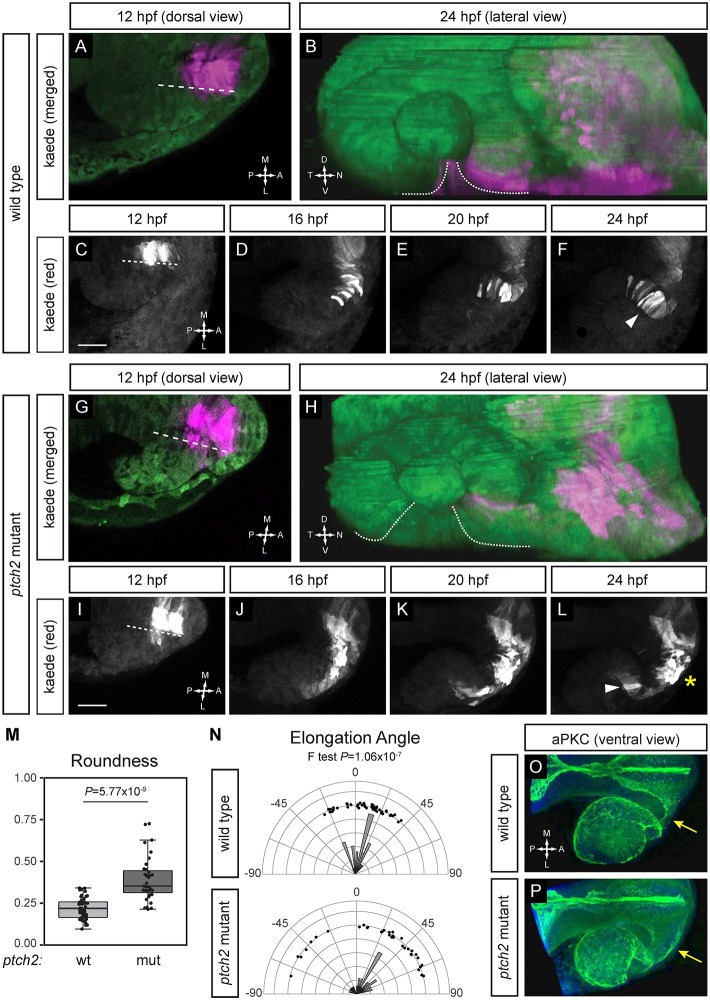
**Migratory behaviors of cells contributing to optic fissure formation are disrupted in the *ptch2^tc294z^* mutant.** (A-F) Wild-type cell movements, cells marked with cytoplasmic Kaede. (A) Merged image of marked cells, 12 hpf, dorsal view. (B) Merged rendering of marked cells, 24 hpf, lateral view. (C-F) Projections from four-dimensional imaging data set of marked cells moving to the ventronasal retina and optic fissure nasal margin. (G-L) Cell movements in the *ptch2^tc294z^* mutant. (G) Merged image of marked cells, 12 hpf, dorsal view. (H) Merged rendering of marked cells, 24 hpf, lateral view. (I-L) Projections from four-dimensional imaging data set of marked cells: most cells (asterisk) remain in the optic stalk. (M) Comparison of roundness in wild-type (wt) and *ptch2^tc294z^* mutant (mut) embryos (unpaired Welch's *t*-test to account for unequal variance). *n*=49 cells from 18 embryos for wt; 35 cells from 11 embryos for mut. (N) Comparison of elongation angle in wild-type (wt) and *ptch2^tc294z^* mutant (mut) embryos (*F*-test to determine the probability that the variances are not significantly different). *n*=49 cells from 18 embryos for wt; 35 cells from 11 embryos for mut. (O,P) aPKC antibody staining in wild-type (O) and *ptch2^tc294z^* mutant (P) embryos, 24 hpf, ventral view. No gross defects in apicobasal polarity in *ptch2^tc294z^* mutants are apparent. Yellow arrows indicate the optic stalk. Dashed lines indicate the boundary between optic vesicle and midline region. Dotted lines indicate optic fissure margins. Arrowheads indicate bipolar cells; asterisk indicates cells with an aberrant morphology. Scale bars: 50 µm.

The data presented thus far suggest that cell motility is affected during *ptch2^tc294z^* mutant optic fissure and stalk formation. To observe motile behaviors and morphologies directly, we first used cytoplasmic Kaede. We sought to visualize cell behavior in the prospective nasal margin; in the *ptch2^tc294z^* mutant, these cells aberrantly contribute to the optic stalk instead of optic cup. Using our cell-tracking data and fate map, we photoconverted prospective nasal margin and some optic stalk cells within the midline region at 12 hpf. In wild-type embryos, cells exit the midline region, adopt an elongated morphology, and move through the forming optic stalk and into the retina (Fig. 4A-F; Movie 11), where they contribute to the ventronasal retina and nasal margin of the optic fissure (Fig. 4B,F; arrowhead; Fig. S2A,A′). A few optic stalk cells are also marked (Fig. 4F).

In *ptch2^tc294z^* mutant embryos, when the same midline region is photoconverted, we observe altered cell behaviors and morphologies (Fig. 4G-L; Movie 12). Initially, cells appear normal, moving out of the midline region and appearing elongated. The first cells to exit the midline continue to exhibit wild-type behaviors, moving through the optic stalk and into the optic cup (Fig. 4L, arrowhead). These cells contribute to nasal retina, but dorsal to the optic fissure. However, the majority of cells take on an aberrant, less-elongated morphology and exhibit motile behaviors within the optic stalk (Fig. 4L, asterisk; Fig. S2B,B′). No marked cells contribute to the disrupted optic fissure, consistent with the nls-Kaede experiment and cell-tracking analyses (Fig. 2F, Fig. 4H).

We quantified the changes in cell morphology within the optic stalk, using mosaic labeling via plasmid DNA injection (pCS2-mCherryCAAX) to visualize individual stalk cells (Fig. S2C-D′). First, we quantified roundness (Fig. 4M), a measure of an object's aspect ratio; a perfect circle has a roundness of 1.0, whereas an extremely elongated ellipse would have a roundness approaching zero. In wild-type embryos, stalk cells are elongated (0.21±0.06); in *ptch2^tc294z^* mutants, stalk cells are significantly less so (0.39±0.13). Second, we quantified elongation angle, that is, the orientation of cells with respect to the anterior-posterior axis of the embryo (Fig. 4N). In wild-type embryos, stalk cells are elongated within a narrow range of angles with respect to the anterior-posterior axis of the embryo; the average cell is elongated closely along the anterior-posterior axis. In *ptch2^tc294z^* mutant embryos, stalk cells are elongated more variably with respect to the anterior-posterior axis; the variance of elongation angles is significantly greater in the *ptch2^tc294z^* mutant than in wild type (*P*=1.06×10^−7^).

The greater variance in *ptch2^tc294z^* mutant stalk orientation angle led us to ask whether tissue polarity is affected: variable cell orientation could be due to disrupted epithelial polarity. Thus, we performed antibody staining for the apical epithelial marker aPKC. In wild-type embryos, aPKC labels the contiguous apical surface of the optic cup, optic stalk and prospective brain (Fig. 4O; Movie 13). In *ptch2^tc294z^* mutant embryos, aPKC still marks an intact apical domain, though the stalk apical surface appears expanded (Fig. 4O,P, yellow arrows; Movie 14). This is in contrast to other mutants, such as *lama1*, in which apical polarity markers do not mark a coherent surface (Bryan et al., 2016). We further sought to assay cell polarity in the stalk with single cell resolution. To this end, we marked centrosomes via RNA injection of an EGFP-tagged Xcentrin construct (Sepich et al., 2011). We find that in both wild-type and *ptch2^tc294z^* mutant embryos, individual centrosomes are oriented toward the apical surface of the optic stalk (Fig. S2E-F′). Therefore, disruption of cell polarity does not underlie the increased variability in stalk cell orientation in *ptch2^tc294z^* mutants.

Our analyses reveal motile behaviors and cell morphologies underlying normal optic fissure and stalk formation; in the *ptch2^tc294z^* mutant, we have identified defects in motility and morphology directly within these cells.

### Loss of *ptch2* acts via both cell-autonomous and non-cell-autonomous mechanisms to disrupt optic fissure and stalk cell movements

Having defined the cellular events underlying normal optic fissure formation and precise defects in the *ptch2^tc294z^* mutant, we sought to determine in which cells the *ptch2^tc294z^* mutation acts to impair cell movements. Does this mutation act directly within migrating prospective optic fissure cells to alter behavior, or does it act in surrounding cells, within the milieu, to alter motile behaviors of neighboring prospective optic fissure cells?

**Fig. 5.**
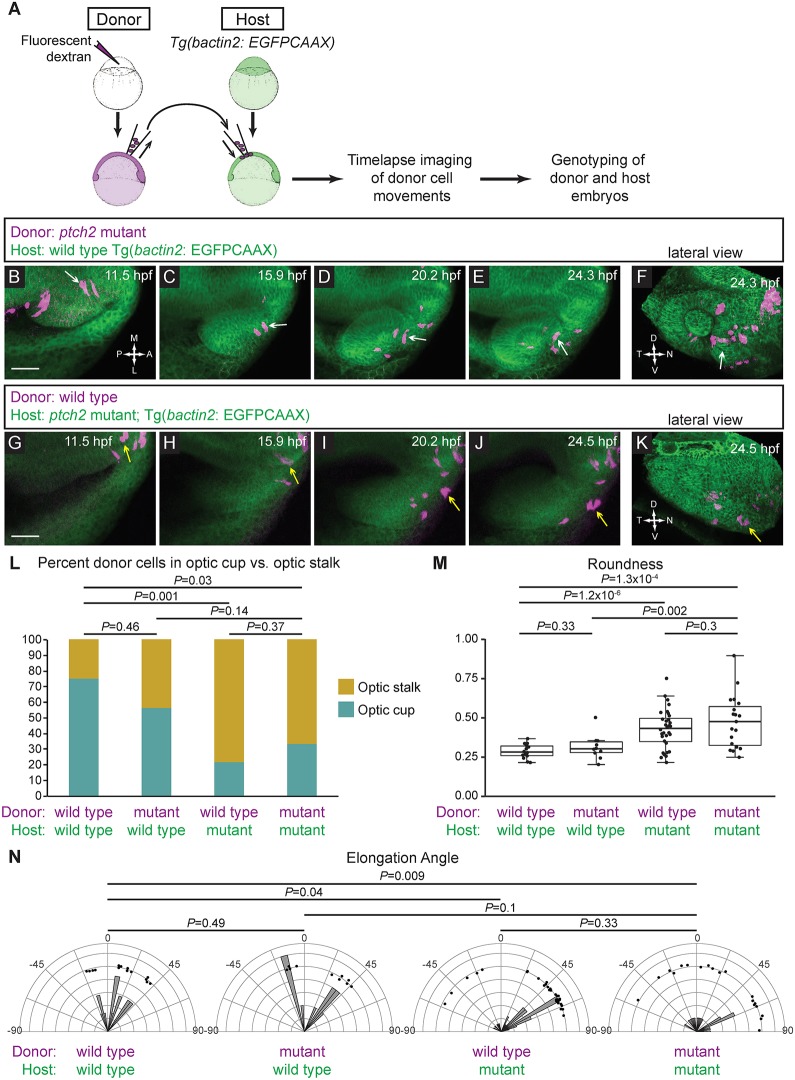
***ptch2^tc294z^* acts in a non-cell-autonomous manner to disrupt cell movements.** (A) Schematic of cell-transplantation approach. Cells were transplanted from donor to host at blastula stages, and time-lapse microscopy performed on embryos in which transplanted cells were in the prospective optic fissure forming region at 12 hpf. After imaging, both donor and host embryos were genotyped. (B-F) Transplantation of *ptch2^tc294z^* mutant cells into a wild-type host. (B-E) Mutant cells exhibit an elongated morphology, move out of the midline region, through the optic stalk, and into the optic cup, where a mutant donor cell contributes to the optic fissure (white arrows). (F) Lateral view of 3D rendering, final time point. White arrow marks a cell that contributes directly to the optic fissure margin. (G-K) Transplantation of wild-type cells into a *ptch2^tc294z^* mutant host. (G-J) Wild-type cells move out of the midline region; few cells move to the central retina within the optic cup. Most cells become less elongated and reside in the optic stalk region (yellow arrows). (K) Lateral view of 3D rendering, final time point. Yellow arrow marks cells that arose from the prospective optic cup/fissure region, but contribute to the optic stalk. (L-N) Quantification of transplantation results. *n*=14 cells (wt into wt); 10 cells (mut into wt); 32 cells (wt into mut); and 19 cells (mut into mut). (L) Proportions of transplanted cells contributing to optic stalk (gold) or optic cup/fissure (teal). Fisher's exact test. (M) Roundness of transplanted cells: morphology reflects host genotype. Unpaired Welch's *t*-test to account for unequal variance. (N) Elongation angle of transplanted cells. *F*-test to determine the probability that the variances are not significantly different. Scale bars: 50 µm.

To distinguish between these possibilities, we utilized blastula cell transplantation (Fig. 5A). At blastula stages, cells were transplanted from donor embryos (injected with fluorescent dextran) to transgenic host embryos [Tg(*bactin2*:EGFPCAAX)^z200^], in which membrane-bound EGFP ubiquitously labels cell membranes. Time-lapse microscopy was carried out on transplanted embryos in which donor cells were found in the midline, the prospective nasal optic fissure and optic stalk region at 12 hpf. We quantified donor cell behaviors using three criteria to distinguish wild-type and mutant cell phenotypes: (1) final position (optic stalk or nasal optic cup/fissure); (2) roundness; and (3) elongation angle.

Control transplantation experiments were performed (Fig. S3; Table S1). When wild-type cells are transplanted into a wild-type host, donor cells contribute more frequently to optic cup and fissure than to stalk; donor cells exhibit wild-type cell behaviors and elongated morphologies; and stalk cells are oriented closely along the anterior-posterior axis of the embryo (Fig. S3A-E; Fig. 5L-N; Movie 15). When mutant cells are transplanted into a mutant host, donor cells contribute more frequently to optic stalk than optic cup/fissure (wt 25% stalk, mutant 66% stalk); exhibit a less elongated morphology (wt roundness 0.28, mutant 0.48); and are oriented with more variability with respect to the anterior-posterior axis (Fig. S3F-J; Fig. 5L-N; Movie 16). These differences are statistically significant between these control transplants (Fig. 5L-N).

When *ptch2^tc294z^* mutant donor cells are transplanted into a wild-type host, mutant cells move out of the midline region, through the optic stalk, and into the optic cup (Fig. 5B-F; Movie 17). These mutant cells (*n*=7 transplants, 23 cells scored) contribute to both optic cup/fissure and optic stalk, with a frequency intermediate to, and not significantly different from, either control condition (43.5% stalk; Fig. 5L; Table S1). Mutant cells maintain an elongated, wild-type morphology (roundness 0.31; Fig. 5F,M, white arrow); are significantly different from mutant control; and are elongated along a narrow range of angles with respect to the anterior-posterior axis, not significantly different from either control (Fig. 5N). We interpret these data to suggest that in this condition, there may be cell-autonomous and non-cell-autonomous factors influencing final donor cell position and orientation angle, but cell elongation is influenced largely by non-cell-autonomous factors.

We next analyzed wild-type donor cells transplanted into a *ptch2^tc294z^* mutant host. These cells (*n*=7 transplants, 41 cells scored) move out of the midline region and contribute primarily to the optic stalk (Fig. 5G-K, yellow arrow; Movie 18), with a frequency similar to mutant control and significantly different from wild-type control (78% stalk; Fig. 5L; Table S1). These cells are less elongated, similar to mutant control and significantly less than wild-type control (roundness 0.43; Fig. 5M), and they are oriented along a wide range of angles, similar to the mutant control, and significantly different from the wild-type control (*P*=0.04; Fig. 5N). Under these conditions, wild-type cells appear more similar to mutant cells. This suggests, in this condition, a significant role for non-cell-autonomous mechanisms regulating these behaviors.

Taken together, our data suggest that a *ptch2^tc294z^* mutant environment disrupts cell position, roundness and orientation angle of transplanted wild-type cells, suggesting the presence of non-cell-autonomous factors. A wild-type host environment rescues the roundness of *ptch2^tc294z^* mutant cells, again suggesting the presence of non-cell-autonomous factors. This same wild-type environment, however, is not sufficient to convert final position and orientation angle of the mutant cells to that of wild type, suggesting the presence of cell-intrinsic factors regulating these specific phenotypes. We conclude that optic fissure and stalk cell position, roundness and orientation angle are governed by a combination of cell-autonomous and non-cell-autonomous factors.

### Hh transcriptional activity is expanded at early stages in the *ptch2^tc294z^* mutant

We next set out to determine the signaling mechanisms by which the *ptch2^tc294z^* mutation disrupts cell motility. Downstream target gene expression is upregulated in the *ptch2^tc294z^* mutant (Koudijs et al., 2008; Lee et al., 2008), yet this was not examined at early stages of optic cup morphogenesis relevant to when we observe disrupted cell motility. Determining, with cellular resolution, the location(s) of cells exhibiting overactive Hh signaling would help identify the primary source(s) of defects in mutants.

To determine if Hh signaling is increased at early stages of optic cup morphogenesis, we established a new transgenic Hh signaling reporter line, Tg(*GBS-ptch2*:H2A-mCherry)^z206^, in which a *ptch2* promoter fragment containing an additional Gli recognition sequence (Shen et al., 2013) drives H2A-mCherry. We also generated a new transgenic reporter line marking Shh-producing cells, Tg(*Shh*:H2A-GFP)^z205^, in which the *Shh* promoter and arABC downstream enhancer elements drive H2A-GFP (Chang et al., 1997; Muller et al., 1999; Ertzer et al., 2007). With these two lines, we generated double-transgenic embryos in which both Shh-producing and Hh-responding cells are marked simultaneously.

**Fig. 6.**
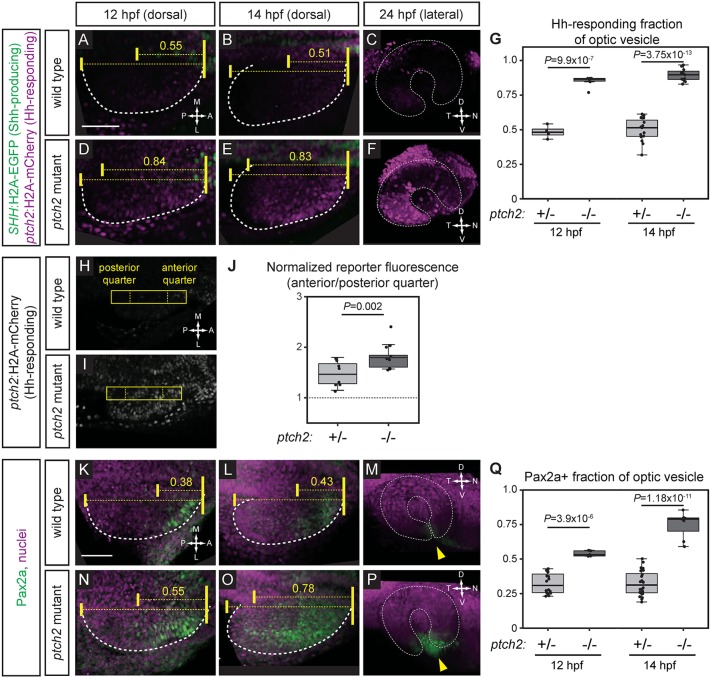
**Hedgehog signaling is increased at early optic vesicle stages in the *ptch2^tc294z^* mutant.** (A-G) Hh signaling reporter expression is expanded in the *ptch2^tc294z^* mutant at 12, 14 and 24 hpf. Embryos are labeled for Shh-producing cells [green; Tg(*SHH*:H2A-GFP)^z205^] and Hh-responding cells [magenta; Tg(*GBS-ptch2*:H2A-mCherry)^z206^]. (A-C) Wild-type double transgenic. (D-F) *ptch2^tc294z^* mutant double transgenic. (G) Quantification of the anterior-posterior fraction of the optic vesicle occupied by reporter-positive cells. *n*=4 wt, 6 mut at 12 hpf; 15 wt, 11 mut at 14 hpf. (H-J) Hh signaling reporter expression [Tg(*GBS-ptch2*:H2A-mCherry)^z206^] is upregulated in the *ptch2^tc294z^* mutant at 13 hpf. A rectangle (44 pixels wide) spanning the anterior-posterior length of the optic vesicle was drawn. Fluorescence intensity in the anterior quarter was normalized to the posterior quarter. A value of 1 (J, dotted line) represents equal fluorescence intensity in the quarters. (H) Wild-type transgenic. (I) *ptch2^tc294z^* mutant transgenic. (J) Normalized fluorescence intensity. *n*=8 wt, 13 mut. (K-Q) Endogenous Hh target gene expression (Pax2a) is expanded in the *ptch2^tc294z^* mutant at 12, 14 and 24 hpf. Antibody staining performed for Pax2a (green), nuclei counterstained with TO-PRO-3 (magenta). (K-M) Wild type. (N-P) *ptch2^tc294z^* mutant*.* Yellow arrowheads indicate the optic fissure. (Q) Quantification of the anterior-posterior fraction of optic vesicle occupied by Pax2a-positive cells. Unpaired Student's *t*-tests. *n*=16 wt, 5 mut at 12 hpf; 24 wt, 7 mut at 14 hpf. Dashed lines indicate border of optic vesicle. Dotted lines indicate border of optic cup. Yellow brackets indicate anterior-posterior extents of the optic vesicle and either reporter expression or Pax2a staining. The number above indicates the fraction of the optic vesicle occupied by the reporter expression or Pax2a staining. Scale bars: 50 µm.

At early optic cup morphogenesis stages (12 and 14 hpf), the Hh signaling reporter is significantly expanded along the anterior-posterior axis in *ptch2^tc294z^* mutant embryos compared with wild type (Fig. 6A,B,D,E,G). Expansion persists through the end of optic cup formation (24 hpf; Fig. 6C,F). This suggests that Hh signaling in the *ptch2^tc294z^* mutant is significantly expanded at time points relevant to the disrupted cell movements.

In addition, reporter expression also appears increased in intensity. To quantify reporter expression, we used a region of interest capturing the anterior-posterior length of the optic vesicle, and measured fluorescence intensity (Fig. 6H-J). We normalized fluorescence intensity in each embryo by dividing the anterior quarter by the posterior quarter (where specific reporter expression was not observed); the reporter is more strongly induced in *ptch2^tc294z^* mutants compared with wild-type embryos (Fig. 6J).

To determine whether these results reflect changes in signaling or simply fluorescent reporter protein perdurance, we performed antibody staining for an endogenous target of Hh signaling, Pax2a. We find that the domain of endogenous Pax2a localization is significantly expanded along the anterior-posterior axis of 12 and 14 hpf optic vesicles of *ptch2^tc294z^* mutants compared with wild type (Fig. 6K,L,N,O,Q); expansion persists to optic cup stage (Fig. 6M,P). These data indicate that loss of *ptch2* results in significantly expanded and increased Hh transcriptional output during early optic cup morphogenesis, which may play a role in the mechanism by which cell movements are disrupted.

### Overactive Hh signaling acts through a canonical Gli1-dependent pathway to disrupt optic fissure and stalk development

We have shown that overactive Hh signaling in the *ptch2^tc294z^* mutant can lead to increased downstream gene expression. Hh signaling, acting through Gli-dependent transcriptional output, is known as canonical Hh signaling. Additionally, Hh signaling in neuronal growth cones can act more directly on the cytoskeleton, in a transcription-independent manner, through Src-family kinases (SFKs) (Yam et al., 2009). As disrupted cell motility is a key feature underlying morphogenetic defects, we investigated which downstream signaling mechanisms are necessary for *ptch2^tc294z^* mutation to cause coloboma.

**Fig. 7.**
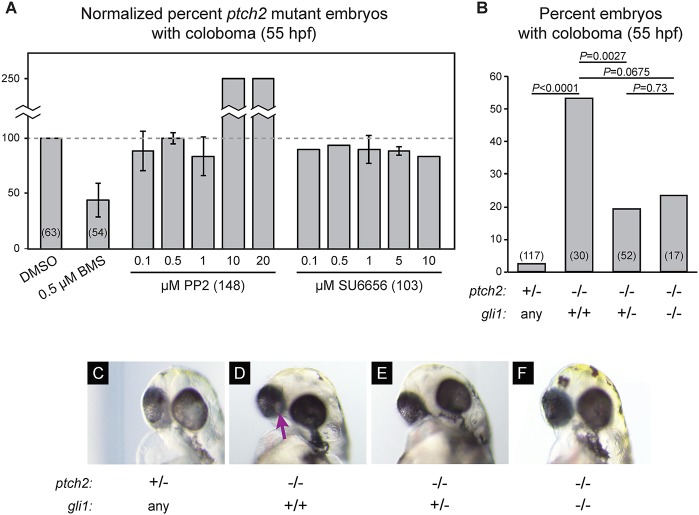
**Canonical signaling via Gli1 is required for coloboma in the *ptch2^tc294z^* mutant.** (A) Inhibition of non-canonical Hh signaling via Src-family kinases (SFKs) does not rescue coloboma in the *ptch2^tc294z^* mutant. Graph shows percentage of *ptch2^tc294z^* mutant embryos with coloboma, normalized to the DMSO control group. (B) Genetic loss of one or both copies of *gli1* (using *gli1^ts269^*) partially rescues the coloboma phenotype in the *ptch2^tc294z^* mutant. Percentage of embryos with coloboma is shown. Numbers in parentheses indicate *n*. (C-F) Representative images of embryos from the genetic experiments shown in B, 55 hpf. (C) *ptch2^+/−^; gli1^any^*. (D) *ptch2^−/−^; gli1^+/+^*. (E) *ptch2^−/−^; gli1^+/−^*. (F) *ptch2^−/−^; gli1^−/−^*. Arrow indicates coloboma.

To test a role for non-canonical Hh signaling via SFKs, we utilized two structurally unrelated SFK inhibitors, PP2 and SU6656 (Molina et al., 2007; Murphy et al., 2011; Gallardo et al., 2015). We treated embryos from 5.5 to 13 hpf, as previously established for rescue of the *ptch2^tc294z^* mutant coloboma with cyclopamine (Lee et al., 2008). The coloboma phenotype is incompletely penetrant at 52 hpf; therefore, the percentage of embryos displaying coloboma was normalized to DMSO control within each individual experiment. As a positive control, we treated embryos with the Smo inhibitor BMS-833923 (Armstrong et al., 2017), which yields ∼50% rescue (Fig. 7A). In contrast, neither SFK inhibitor rescues the coloboma phenotype (PP2 exacerbated it). We conclude that non-canonical Hh signaling via SFKs is not required to cause coloboma in the *ptch2^tc294z^* mutant.

To test a role for Gli transcription factors, we carried out a genetic epistasis experiment in which the *ptch2^tc294z^* mutant was crossed to the *gli1^ts269^* loss-of-function mutant (Brand et al., 1996; Karlstrom et al., 1996). Gli1 is the major transcriptional activator downstream of Hh signaling in zebrafish (Karlstrom et al., 2003), and we reasoned that if Gli1 activity is necessary for coloboma, loss of *gli1* would rescue this phenotype. For these experiments, coloboma was scored at 52-55 hpf (Lee et al., 2008). We find that, indeed, loss of one or both copies of *gli1* partially rescues the coloboma phenotype in *ptch2^tc294z^* mutants (Fig. 7B-F). Although it was initially surprising that loss of one copy of *gli1* might confer rescue, haploinsufficient phenotypes have been described with this specific line; *gli1^ts269^* heterozygotes can display phenotypes (Tyurina et al., 2005). This rescue is incomplete when removing either one or both copies of Gli1 (Fig. 7B), suggesting other contributions, possibly from other Gli proteins (such as Gli2 or Gli3), or other Hh signaling outputs. We conclude that overactive Hh signaling acts at least in part via canonical Gli1 activity to disrupt eye development.

## DISCUSSION

We describe here, for the first time, the cell movements underlying optic fissure and stalk formation. Somewhat surprisingly, we find that neighboring cells contribute to opposing margins of the optic fissure. This suggests that optic fissure formation may not be the passive byproduct of optic cup invagination: if the fissure margins were simply folded together, we might expect opposing margins to be derived from distant positions, with cells taking reversed trajectories to abut each other on either side of the fissure. In contrast, we see that neighboring cell populations take similar trajectories but come to reside on opposite margins. This suggests that there may be an active process, potentially involving tissue involution or folding, to split these cell populations. Future work will address the mechanisms underlying this process.

Uncovering the cell movements underlying optic fissure and stalk formation allowed us to establish a framework to identify cell migration and motility defects that might contribute to impaired development and coloboma. We then determined the defects in cell movements underlying optic fissure and stalk formation in the *ptch2^tc294z^* mutant (Fig. 3). By performing four-dimensional cell tracking, we detected motility defects in cells that normally contribute to optic fissure and optic stalk, but in the mutant fail to achieve their correct position owing to defective motility. Cells that should contribute to optic fissure and cup instead contribute to the optic stalk. Temporal fissure cells in the *ptch2^tc294z^* mutant arise from a novel location, which, under normal conditions, contributes to temporal retina (Fig. S1). At 12 hpf, this novel origin does not exhibit increased Hh target gene expression in *ptch2^tc294z^* mutants (Fig. 6); therefore, we hypothesize that the novel trajectory observed is secondary to failure of midline cells to properly move into the optic stalk and optic cup.

**Fig. 8.**
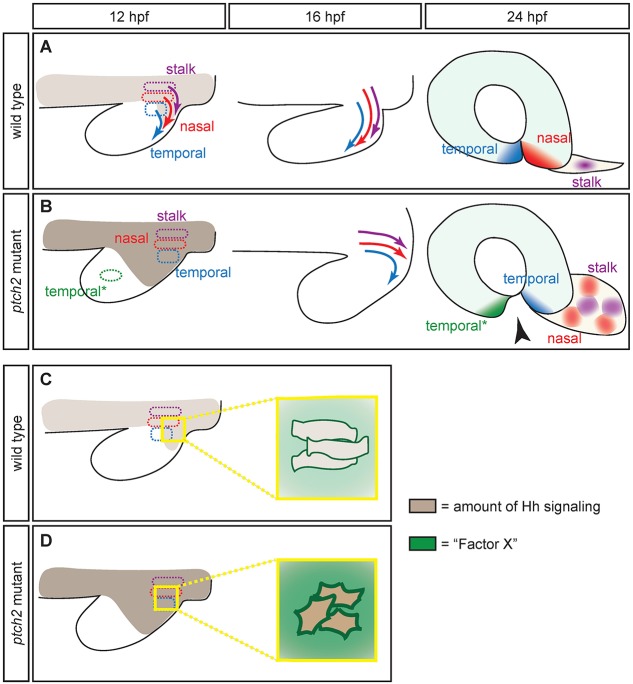
**Model for optic fissure and stalk cell movements and regulation by Hh signaling.** (A) Optic fissure and stalk cell movements in wild type: adjacent cell populations (from origins marked as dashed circles) move along a J-shaped trajectory to occupy the optic fissure margins and stalk. (B) Optic fissure and stalk cell movements in the *ptch2^tc294z^* mutant: cells that should normally contribute to the optic cup are altered in their movement and do not achieve their correct position. (C) Proposed model for Factor X, a non-cell-autonomously acting cell surface or secreted molecule and downstream target of Hh signaling. Under wild-type conditions, Factor X is expressed at a level that ensures appropriate regulation of optic fissure and stalk cell morphology and orientation. (D) In the *ptch2^tc294z^* mutant, overexpression and expansion of Factor X leads to diminished elongation and defective motility.

Our results indicate that cell motility can be affected by overactive Hh signaling; we sought to understand how this might occur. Hh signaling can act through both canonical Gli-dependent and non-canonical Gli-independent pathways; we find evidence for Gli1-dependent activity but not non-canonical SFK signaling in the etiology of the *ptch2^tc294z^* mutant coloboma phenotype. Using cell transplantation, we also find that the *ptch2^tc294z^* mutation acts via both cell-autonomous and non-cell-autonomous mechanisms to regulate cell position and orientation, but non-cell-autonomous mechanisms largely regulate cell elongation. Taken together, we propose the following model (Fig. 8): in a wild-type embryo, the nasal and temporal optic fissure margins and optic stalk are derived from neighboring cell populations. The cells move in a wide J-shaped trajectory, with temporal cells moving first, followed by nasal cells, followed by stalk cells (Fig. 8A). In the *ptch2^tc294z^* mutant, cells derived from the prospective optic fissure and stalk domains do not achieve their correct positions (Fig. 8B) and have aberrant morphology: they are significantly less elongated, and more variably oriented with respect to the anterior-posterior axis of the embryo. Although cell morphology is altered, tissue polarity is still intact. We speculate that optic fissure and stalk formation fail because inappropriate positioning of prospective fissure and stalk cells impedes formation of a functional structure.

How might overactive Hh signaling actually affect cell motility? In our working model, we propose that in a wild-type embryo, Hh signaling leads to Gli1-dependent induction of crucial factors that control cell elongation, orientation and motility. At least one factor, ‘Factor X’ (Fig. 8C, green) acts in a non-cell-autonomous manner, potentially as a cell surface or secreted molecule. We hypothesize that expression level of Factor X is crucial for regulation of motile cell behaviors, either directly or indirectly, and that in the *ptch2^tc294z^* mutant, expansion and overexpression of Factor X leads to altered cell elongation and orientation, and disruption of motility (Fig. 8D). We are currently working to identify Factor X.

Our results reveal the cellular basis of the optic fissure and stalk defect in one coloboma model: moving forward, and using our wild-type analysis as a framework for comparison, it will be important to examine other coloboma models with aberrant optic fissure and stalk formation. We speculate that there may be many ways to disrupt this process, and multiple cell populations that can be affected, reflecting the genetic heterogeneity found in the human condition.

## MATERIALS AND METHODS

### Zebrafish husbandry and mutant/transgenic lines

All zebrafish husbandry (*Danio rerio*) was performed under standard conditions in accordance with University of Utah Institutional Animal Care and Use Committee (IACUC) Protocol approval (Protocol #18-02006). Embryos (Tü or TL strains) were raised at 28.5-30°C and staged according to time post fertilization and morphology (Kimmel et al., 1995). Mutant lines were previously described: *ptch2*/*blowout^tc294z^* (Karlstrom et al., 1996; Koudijs et al., 2008; Lee et al., 2008); *gli1/detour^ts269^* (Brand et al., 1996; Karlstrom et al., 1996).

For genotyping, genomic DNA was extracted from single embryos or adult fins, incubated at 95°C in 0.05 M NaOH for 30 min, then neutralized with 1 M Tris pH 8.0. The *ptch2* locus was genotyped by one of two methods. A CAPS assay (Konieczny and Ausubel, 1993) with the following primers was used: ptch2_F: 5′-CCATGATAAGTACGACACCACTGGAGAG-3′, ptch2_R: 5′- CACTACACCAAATCCCTGATGGATGG-3′. The *ptch2^tc294z^* mutation creates an *Ava*II cut site; the wild-type allele is uncut. Alternatively, we developed an HRMA protocol (Parant et al., 2009) with the following primers: ptch2HRMA_F: 5′- CTGCACCTTCCTGGTGTGTG-3′, ptch2HRMA_R: 5′- GGTAGAAATGGATTAGAGTGAGAGGAA-3′. These primers generate a 96 bp amplicon; homozygous wild-type and mutant duplexes melt at 87.6°C and 88.5°C, respectively. The *gli1* locus was genotyped using a dCAPS assay (Neff et al., 1998) with the following primers: gli1_F: 5′-GTGCCAGCGATCCGGTGCGATCC-3′, gli1_R: 5′- CATTCCTGCACCCTGGTATTGCATCC-3′. The mutant allele combined with the dCAPS primer creates a cut site for *Hpy*CH4III and the wild-type allele is uncut.

### RNA synthesis and nucleic acid injections

Capped RNA was synthesized using pCS2 templates (pCS2-EGFP-CAAX, pCS2FA-H2A.F/Z-mCherry, pCS2FA-nls-Kaede, pCS2-rSmoM2-EGFP, pCS2FA-Kaede, pCS2-EGFP-Xcentrin, pCS2FA-mCherry-CAAX, pCS2FA-Tol2tranposase) and mMessage mMachine SP6 kit (Ambion). RNA was purified (Qiagen RNeasy Mini Kit) and ethanol precipitated. For fluorescent proteins, 300-500 pg RNA was injected into one-cell embryos. For activated Smoothened, 175-200 pg rSmoM2-EGFP RNA was injected into the one-cell embryos. To label centrosomes, 200 pg EGFP-Xcentrin RNA and 200 pg mCherry-CAAX RNA were injected into one-cell embryos. For mosaic labeling of cells (for cell morphology quantification), 25 pg of pCS2-mCherryCAAX DNA was injected into one-cell embryos.

### Transgenic constructs and establishment of stable lines

For the transgenic line with ubiquitous expression of membrane-bound GFP, Tg(*bactin2*:EGFPCAAX)^z200^, the bactin2 promoter (p5E-bactin2) was recombined with a middle entry clone of EGFP-CAAX (pME-EGFPCAAX) and a 3′ clone of the SV40 late polyA signal sequence (p3E-polyA) into the Tol2 transposon-flanked destination vector, pDestTol2pA2 (Kwan et al., 2007).

For the transgenic line marking Shh-producing cells, Tg(*SHH*:H2A-EGFP)^z205^, the −2.2 kb *Shh* promoter and the downstream enhancer elements (arABC) were a kind gift from Ferenc Müller, Yavor Hadziev and Uwe Strähle (Chang et al., 1997; Muller et al., 1999; Ertzer et al., 2007). The −2.2 kb *Shh* promoter was cloned into a multisite Gateway 5′ entry vector, and the arABC elements were cloned into a multisite Gateway 3′ entry vector. These two pieces were used with a Gateway middle entry clone of H2A-EGFP-polyA (pME-H2A-EGFP-pA) to generate the full transgene expression construct in the Tol2 transposon-flanked destination vector, pDestTol2pA2 (Kwan et al., 2007).

For the transgenic line marking Hh-responding cells, Tg(GBS-*ptch2*:H2A-mCherry)^z206^, the 900 bp *ptch2* promoter containing an additional Gli recognition sequence (in a 5′ Gateway entry vector) was a kind gift from Rolf Karlstrom (Shen et al., 2013). This was recombined with a middle entry clone of H2A-mCherry and a 3′ entry clone of the SV40 late polyA signal sequence to generate the full transgene expression construct in the Tol2 transposon flanked destination vector, pDestTol2pA2 (Kwan et al., 2007).

Assembled transgene expression constructs were co-injected with *Tol2* transposase (30 pg DNA+25 pg transposase RNA) into one-cell embryos. Injected embryos were screened for fluorescence reporter expression at 24 hpf and raised to adulthood.

### Coloboma scoring

Embryos were individually screened and scored for coloboma at 52-55 hpf using an Olympus SZX16 stereomicroscope. Eyes that exhibited abnormal morphology outside of the coloboma phenotype were excluded from final analysis. Viewing the back of the eye being scored, and focusing at the depth of the RPE, embryos that scored as positive for coloboma displayed an expanded region lacking pigmentation in the area of the optic nerve head; in a positive embryo, this area was distinctly wider and more open than the rest of the optic fissure that was undergoing fusion at the ventral side of the optic cup. Pigmentation and fissure fusion along the ventral surface of the optic cup can show variability with timing among both wild-type and *ptch2^tc294z^* mutant embryos; therefore, simple (often subtle) expansion of a hypopigmented region along the ventral surface of the optic cup (away from the optic nerve head) was not sufficient. Embryos that were not clearly positive for coloboma were counted as negative.

All genetic experiments and pharmacological treatments were blindly scored. Embryos were subsequently genotyped as described above.

### Antibody staining

Embryos were raised until the stage of interest and fixed in 4% paraformaldehyde (Electron Microscopy Science, 15710) overnight at 4°C. Embryos were permeabilized in PBST (PBS with 0.1% Triton X-100) and blocked in 2% bovine serum albumin in PBST at room temperature. Primary antibodies were diluted in blocking solution and incubated overnight at 4°C . Secondary antibodies were co-incubated with 1 μM TO-PRO-3 iodide (Life Technologies, T3605) for 4 h at room temperature or overnight at 4°C. Between incubations, embryos were washed three times in PBST, 20 minutes per wash. Primary antibodies were: anti-Pax2a (1:200; Genetex, GTX128127), anti-aPKC (1:100; Santa Cruz Biotechnology, sc-216), anti-phospho-histone H3 (1:500; Abcam, ab14955). Secondary antibodies were: Alexa Fluor 488-conjugated goat anti-rabbit (Life Technologies, A-11008), Alexa Fluor 568-conjugated goat anti-rabbit (Life Technologies, A-11011), or Alexa Fluor 568-conjugated goat anti-mouse (Life Technologies, A-11001). Embryos were cleared in 70% glycerol for imaging.

### Imaging

For time-lapse imaging, embryos were dechorionated at the relevant developmental stage, embedded in 1.6% low melting point agarose (in E2+gentamycin) in Delta T dishes (Bioptechs, 0420041500C). Images were acquired using a Zeiss LSM710 laser-scanning confocal microscope. E2+gentamycin was overlaid, and the dish covered to prevent evaporation. No stage heater was used. Based on the timing of lens separation from the overlying ectoderm, we estimate that sample temperature was very close to 28.5°C. All imaging was performed with a 40× water-immersion objective (1.2 NA).

For cell tracking, four-dimensional datasets were acquired with the following parameters: 60-63 *z*-sections, 2.1 µm *z*-step, 2.75 min between *z*-stacks. For imaging cell-transplantation experiments, four-dimensional datasets were acquired with the following parameters: 60-100 *z*-sections, 2.1 µm *z*-step, 6-12 min between *z*-stacks.

Kaede photoconversion was performed using Zeiss Zen software to select and expose a region of interest (ROI) to 405 nm light for 15-20 s. Photoconversion efficiency was assayed by loss of green and gain of red fluorescence in the ROI.

### Image analysis: four-dimensional cell tracking/visualization

Cell tracking was performed according to Kwan et al. (2012), using LongTracker. Briefly, four-dimensional data sets were pre-processed as previously described (Kwan et al., 2012), linearly adjusting brightness and contrast. LongTracker was used to select nuclei at specific time points and *z* positions, while simultaneously viewing *xz* and *yz* reslices and adjacent frames in *z* and *t*, stepping through the data set forward or backward in time. For accurate cell tracking, we required at least 50% nuclear pixel overlap between time points. Data were exported as a spreadsheet of nuclear centroid position, nuclear TIFF images, or three-dimensional trajectories. Trajectories were checked for discontinuities in four dimensions using FluoRender (Wan et al., 2012). Therefore, tracking was performed and checked in four dimensions. Figures show a dorsal view of the tracking data, and the pseudocolored signal is the actual nuclei that were tracked. Trajectories were generated by summing the nuclear masks over time.

### Image analysis: optic fissure opening angle

Three-dimensional data sets of live embryos labeled for membranes (EGFP-CAAX) were oriented in FluoRender (Wan et al., 2012) for a lateral view. In this lateral view, we focused in on the midpoint of the lens, using the lateral cutaway tool. TIFF images of this view were captured from FluoRender, and the opening of the optic fissure was measured using the angle tool in Fiji. The rays of the angle projected along the optic fissure margins, with the vertex positioned at the center of the lens.

### Image analysis: region/area quantification (Shh reporter, Pax2a)

#### Reporter extent measurement

Embryo orientation was adjusted using FluoRender to ensure a standardized dorsal view. Typically, a ‘trail’ of reporter expression was observed along the lateral edge of the optic vesicle. The medial and lateral anterior-posterior extent of reporter expression were measured; the anterior-posterior midpoint was then calculated and plotted as a ratio of the entire optic vesicle length.

#### Reporter fluorescence intensity measurement

Measurements were performed on a single slice at the dorsal-ventral midpoint of the optic vesicle. Images were rotated in ImageJ/Fiji, such that the optic vesicle was oriented vertically. A rectangular ROI was drawn (using the rectangle tool): the width of the rectangle was 44 pixels (30.44 µm), and the length of the rectangle spanned the anterior-posterior length of the optic vesicle. Using a vertical profile, the command ‘Plot Profile’ was used to quantify fluorescence intensity at each vertical pixel position along the entire rectangle. Average fluorescence intensity measurements were calculated for the anterior and posterior quarters of the rectangle; for each embryo, the relative fluorescence intensity measurement was normalized as the ratio of the anterior quarter to the posterior quarter.

#### Pax2a extent measurement

Embryo orientation was adjusted using FluoRender to ensure a standardized dorsal view. The anterior-posterior extent of Pax2a staining was measured as a ratio of entire optic vesicle length.

### Image analysis: cell morphology and orientation angle quantification (Kaede, mosaics and transplants)

#### Roundness measurement

Embryos were oriented dorsally in FluoRender, and TIFF images captured of dispersed cells in the stalk, for which morphology was easily visualized. An outline was drawn around the cell using the freehand tool in Fiji/ImageJ, and roundness was measured (as one of the shape descriptors in the Measurements).

#### Orientation angle

Similar to roundness, embryos were oriented dorsally in FluoRender, and TIFF images captured of dispersed cells in the stalk, for which morphology was easily visualized. The angle tool in Fiji/ImageJ was used: one ray extended along the anterior-posterior axis of the embryo, and the other ray extended along the long axis of the cell.

### Image analysis: three-dimensional cell counting (custom MATLAB script, ‘Abacus’)

Nuclear signal in three-dimensional data sets was used for cell counting.

#### Preprocessing

Nuclear signal was in 8-bit gray level resolution. A Hi-Lo lookup table (ImageJ/Fiji) was used to linearly adjust the signal such that the background noise level was set to zero, and the brightest pixels were just below 255.

#### Abacus algorithm (see also supplementary Materials and Methods for MATLAB code)

The image *z*-stack with pixel size=dx and *z*-step=dz) was low-pass filtered using a 3D Gaussian smoothing kernel with a standard deviation in the *z* direction (σ_z_) that is proportional to the standard deviation in the *xy*-plane (σ_xy_) such that σ_z_=σ_xy_(dx/dz). A threshold value, L, was set to eliminate background noise, and several linearly spaced isosurfaces were calculated to visually represent the outer edge of the fluorescent nuclear markers. Using the filtered *z*-stack, regional maxima with a minimum height h, were located and the centroids of those regions calculated. Centroid markers were overlaid with the isosurfaces to visually verify if the centroid locations correlate with the centers of the nuclear marker outlines. The parameters σ_xy_, σ_z_, L and h were adjusted until the centroid markers matched the center locations of the nuclear marker isosurfaces. Visual verification of the centroid and isosurface overlay was performed by stepping through narrow cross-sectional views of the data.

### Box and whisker plots

Box and whisker plots were generated using the ggplot2 package in R. The band inside the box is the median. The upper and lower ‘hinges’ correspond to the first and third quartiles. The upper whisker extends from the upper hinge to the highest value within (1.5 * IQR), where IQR is the inter-quartile range. The lower whisker extends from the lower hinge to the lowest value within (1.5 * IQR). Data points outside of the ends of the whiskers are outliers.

### Blastula-stage transplantation and analysis

Donors or hosts for the wild-type sibling condition were either embryos from incrosses of Tü wild types, or heterozygote siblings from crosses of *ptch2^tc294z^* heterozygotes and homozygotes (identified by genotyping after the experiment). Donor or host embryos for the mutant condition were from crosses of *ptch2^tc294z^* heterozygotes and homozygotes (identified by genotyping after the experiment). Host embryos were additionally Tg(*bactin2*:EGFPCAAX)^z200^; they were marked with ubiquitous expression of EGFP-CAAX.

Donor embryos were injected at the one-cell stage with 1 nl Alexa-568 dextran (Thermo Scientific, D22912). At the mid-blastula stage, donor and host embryos were dechorionated and transferred to agar-coated dishes in E3+1% pen/strep (Invitrogen, 15070063). Up to 50 cells were removed from donor embryos, and approximately 10-15 cells transferred to each of four host embryos. Donor embryos were immediately subjected to genomic DNA extraction and genotyping, and host embryos were transferred to agarose-coated wells of a 24-well culture dish. At ∼11 hpf, host embryos were screened for donor cells in the eye region and mounted for time-lapse imaging. Embryos were screened for a second time under the confocal microscope for donor cells residing within the midline region, the area from which prospective nasal optic fissure cells arise. After time-lapse imaging, embryos were de-embedded and subjected to genotyping.

Transplantation analysis was carried out specifically on cells that initially resided in the region of interest: the midline region, from which the nasal optic fissure and optic stalk are derived. Cells were scored for final position (optic fissure/cup or optic stalk), roundness, and orientation angle.

### Pharmacological experiments

Fertilized embryos were grown until 5.5 hpf, at which time they were dechorionated and placed into agarose-coated wells of a 6-well culture dish. Embryos were cultured in 7 ml of E3 with indicated concentrations of BMS-833923 (Cayman Chemical, 16240), PP2 (Calbiochem, 529573), SU6656 (Calbiochem, 572635), or DMSO (Sigma, D8418), based on published reports in zebrafish (Molina et al., 2007; Murphy et al., 2011; Gallardo et al., 2015; Armstrong et al., 2017), as well as our own dose-response experiments (data not shown). At 13 hpf (8 somite stage), embryos were removed from pharmacological treatment, washed once, and transferred to fresh agarose-coated dishes containing E3. Coloboma scoring was performed blind at 55 hpf, and embryos subsequently genotyped.

## Supplementary Material

Supplementary information
