# Supplementary material for: Hedgehog signaling regulates cell motility and optic fissure and stalk formation during vertebrate eye morphogenesis
Source: Development. 2018 Nov 19;145(22):dev165068. doi: 10.1242/dev.165068 (PMC6262791; doi:10.1242/dev.165068)
Supplement: Supplementary information [file develop-145-165068-s1.pdf]

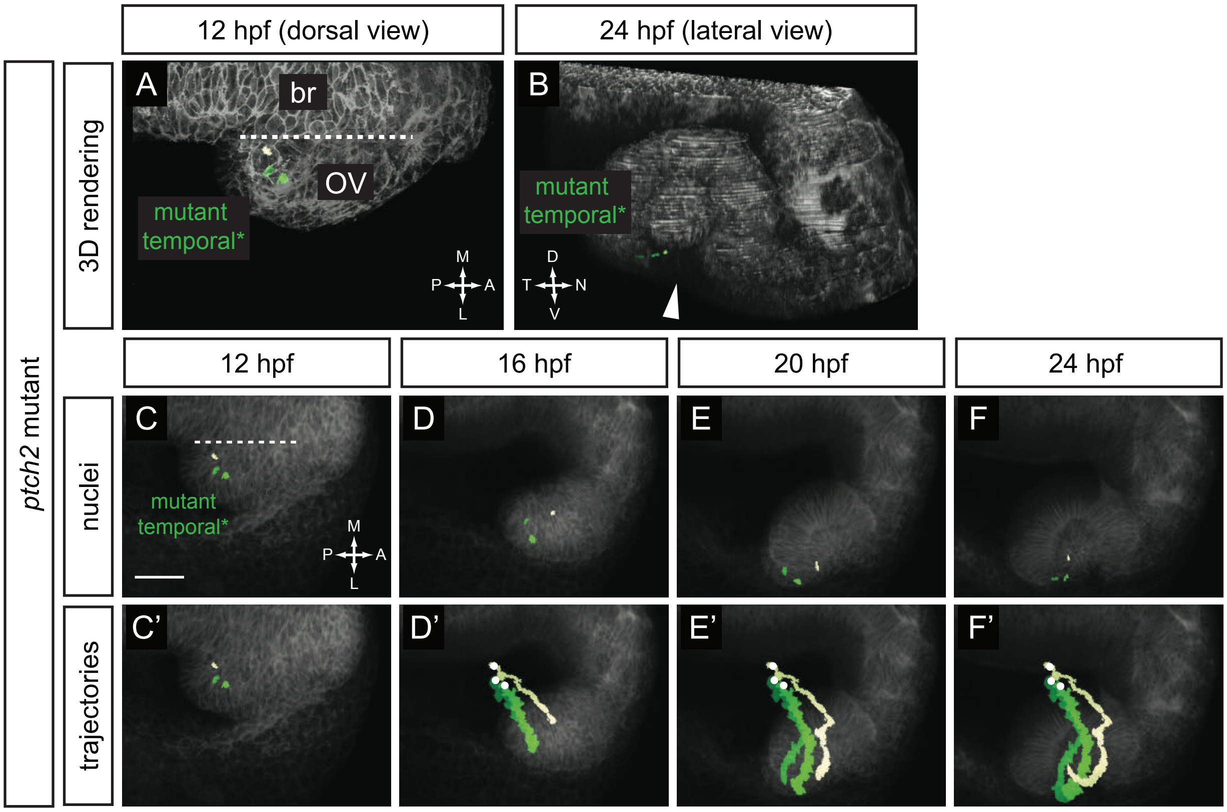

Figure S1. Origin and movement of cells contributing to the temporal optic fissure margin in the *ptch2*^{tc294z} mutant.
(A-F') *ptch2*^{tc294z} mutant temporal optic fissure cell movements (12-24 hpf). (A) Rendering of nuclei and membrane channel, 12 hpf, dorsal view. (B) Rendering of nuclei and membrane channel, 24 hpf, lateral view. (C-F) Nuclei over membrane channel average projection. (C'-F') Trajectories over membrane channel average projection. Cells execute a novel trajectory. Scale bar, 50 μm.

Figure S2. Single cell morphologies and cell polarity.

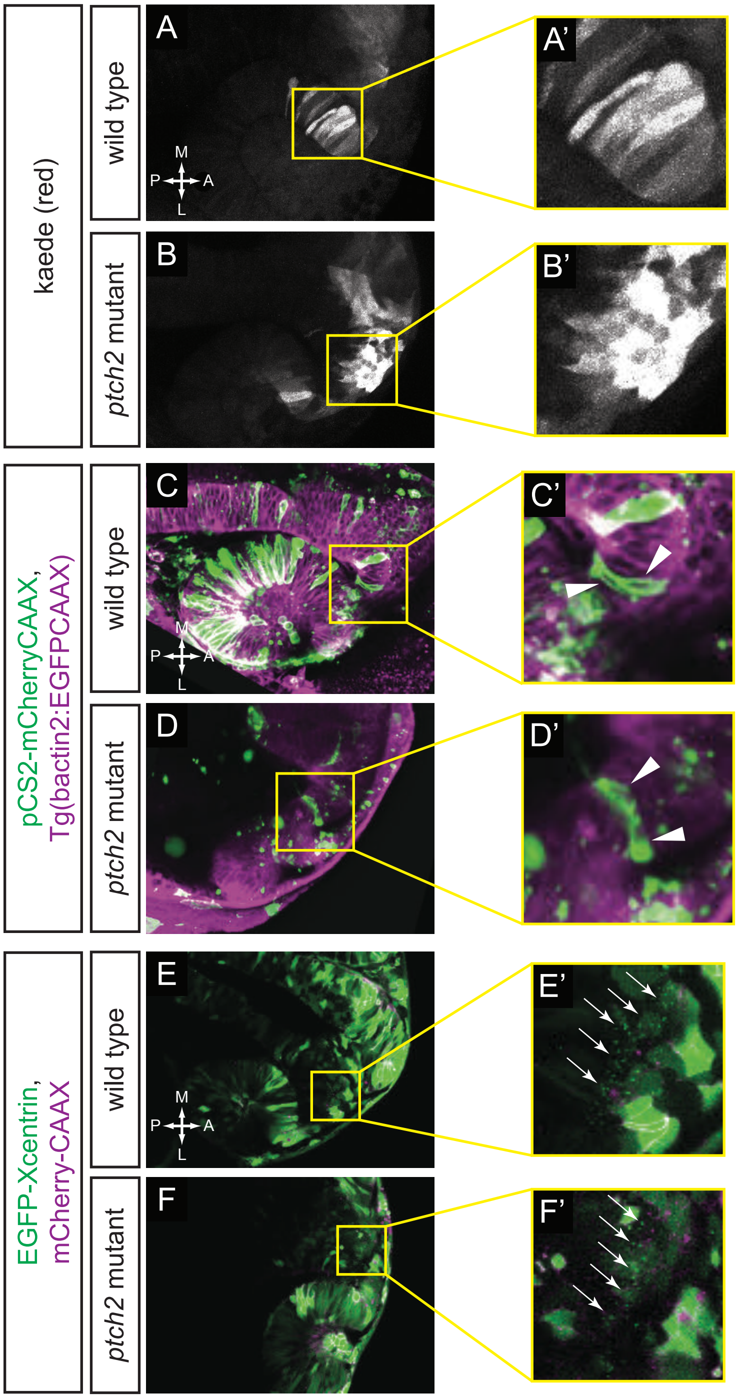

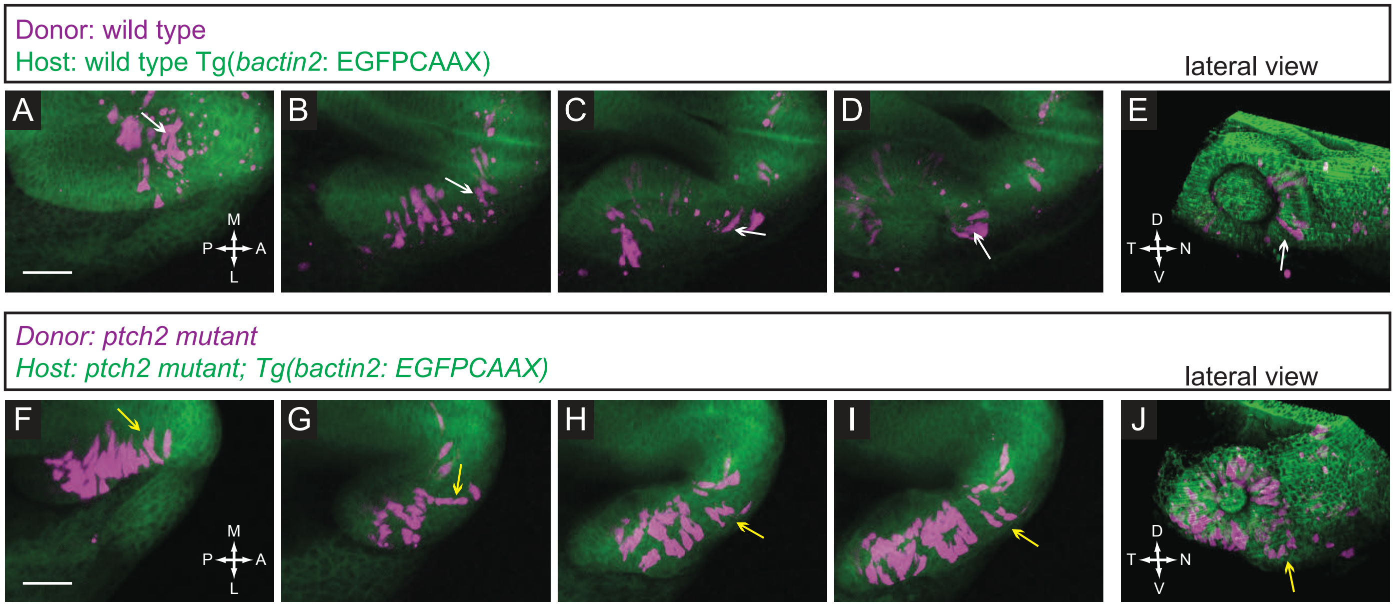

Figure S3. Localization and movement of transplanted cells in control conditions. (A-E) Transplantation of wild type donor cells into a wild type host. (A-D) Donor cells move out of the midline region, through the prospective optic stalk, and into the optic cup. Note the bipolar, elongated morphology of the cell contributing to the nasal optic fissure (*white arrows*). (E) Lateral view of 3D rendering of final timepoint.
(F-J) Transplantation of *ptch2*^{tc294z} mutant donor cells into a *ptch2*^{tc294z} mutant host. (F-I) Many donor cells in this movie start already within the optic vesicle. Following cells that emerge from the midline region, some cells exhibit and maintain this morphology through the optic stalk and into the optic cup. Other cells (*yellow arrows*) lose their bipolar morphology and contribute to the optic stalk. (J) Lateral view of 3D rendering of final timepoint.
Scale bar, 50 µm.

Development • Supplementary information

Table S1. Summary of Transplant Data

			MIDLINE STARTING POSITION	ENDING POSITION			
	experiment	D>H genotypes	total # cells in ROI	Nasal Optic Fissure	Cell Morphology	Optic Stalk	Cell Morphology
wt>wt	160601pos1	wt>wt	1	1	elongated	0	
	151104pos1	wt>het	7	1 group	most are elongated	1	most are elongated
	160601pos2	wt>wt	2	0		2	elongated
	160916pos2	wt>wt	3	3	2 elongated, 1 aberrant	0	
		SUBTOTALS	13	9		3	
mut>wt	160115pos1	mut>wt	2	0		2	elongated
	160115pos3	mut>wt	4	4	elongated	0	
	160212pos3	mut>wt	1	1	elongated	0	
	160308pos4	mut>wt	1	1	elongated	0	
	160308pos3	mut>wt	4	0		4	elongated
	160512pos3	mut>het	2	1	elongated	1	elongated
	160525pos2	mut>het	9	6	elongated	3	elongated
		SUBTOTALS	23	13		10	
wt>mut	170127pos2	wt>mut	6	2		4	
	170127pos3	wt>mut	12	5		7	
	170127pos4	wt>mut	8	0		8	
	170127pos5	wt>mut	5	1		4	
	160512pos4	wt>mut	7	0		3 indiv, 4 as a clump	aberrant
	161004pos3	wt>mut	2	1	larger cell, elongated	1	smaller cell, aberrant
	170126pos1	wt>mut	1	0		1	aberrant
		SUBTOTALS	41	9		32	
mut>mut	160421pos1	mut>mut	4	2		0	
	170209pos2	mut>mut	11	5	elongated	clump of 2 near a clump of 4	aberrant
	170216pos1	mut>mut	2	0		2	aberrant
	170216pos3	mut>mut	2	0		2	aberrant
	170216pos4	mut>mut	4	0		4	1 elongated, 3 aberrant
		SUBTOTALS	23	7		14	

Development • Supplementary information

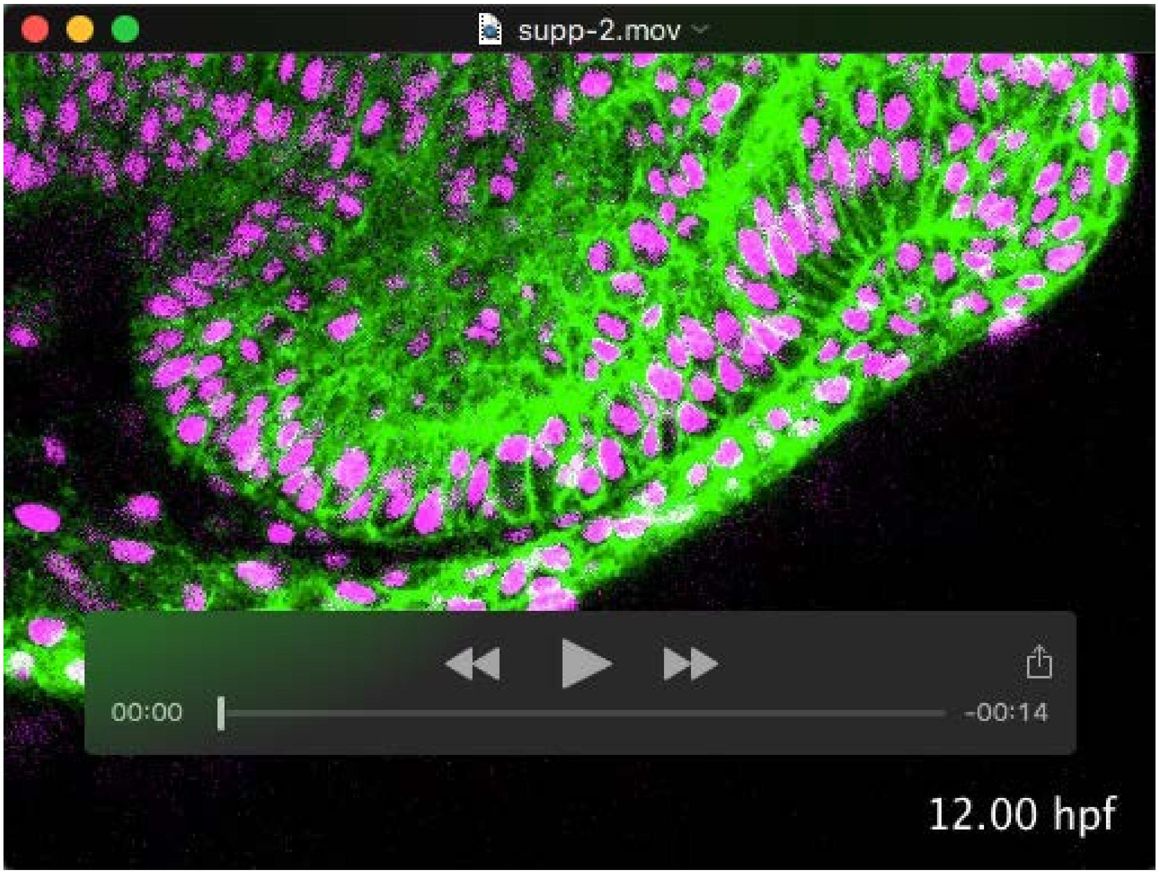

Movie 1. Timelapse of wild type optic cup morphogenesis. Single confocal section from a 4D data set showing right optic vesicle, ~12-24 hpf. Time interval between z-stacks, 2.75 minutes. Dorsal view. Embryo labeled with EGFP-CAAX (membranes, *green*) and H2A.F/Z-mCherry (nuclei, *magenta*).

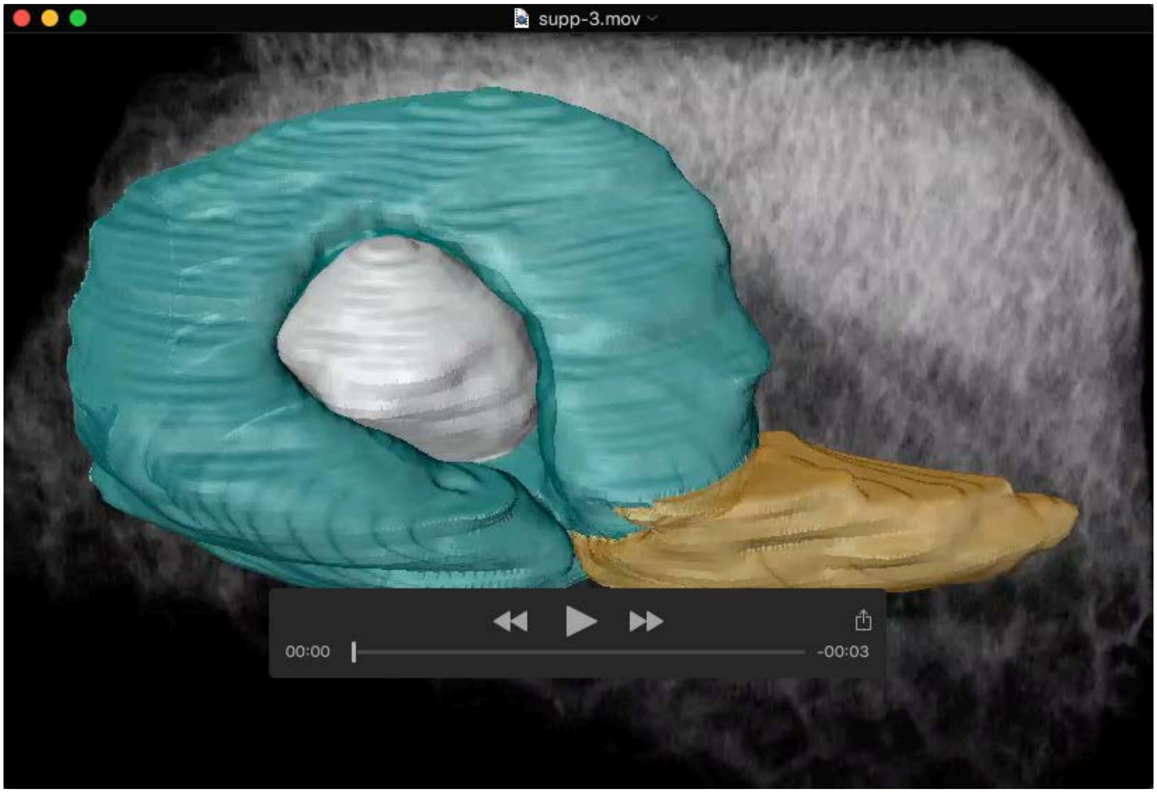

Movie 2. Segmentation of wild type optic cup to visualize normal optic fissure morphology. Lateral view of 3-dimensional volume rendering of manually segmented optic cup (*teal*), lens (*gray*), and optic stalk (*gold*), 24 hpf. The optic fissure margins are closely apposed at the ventral side of the optic cup.

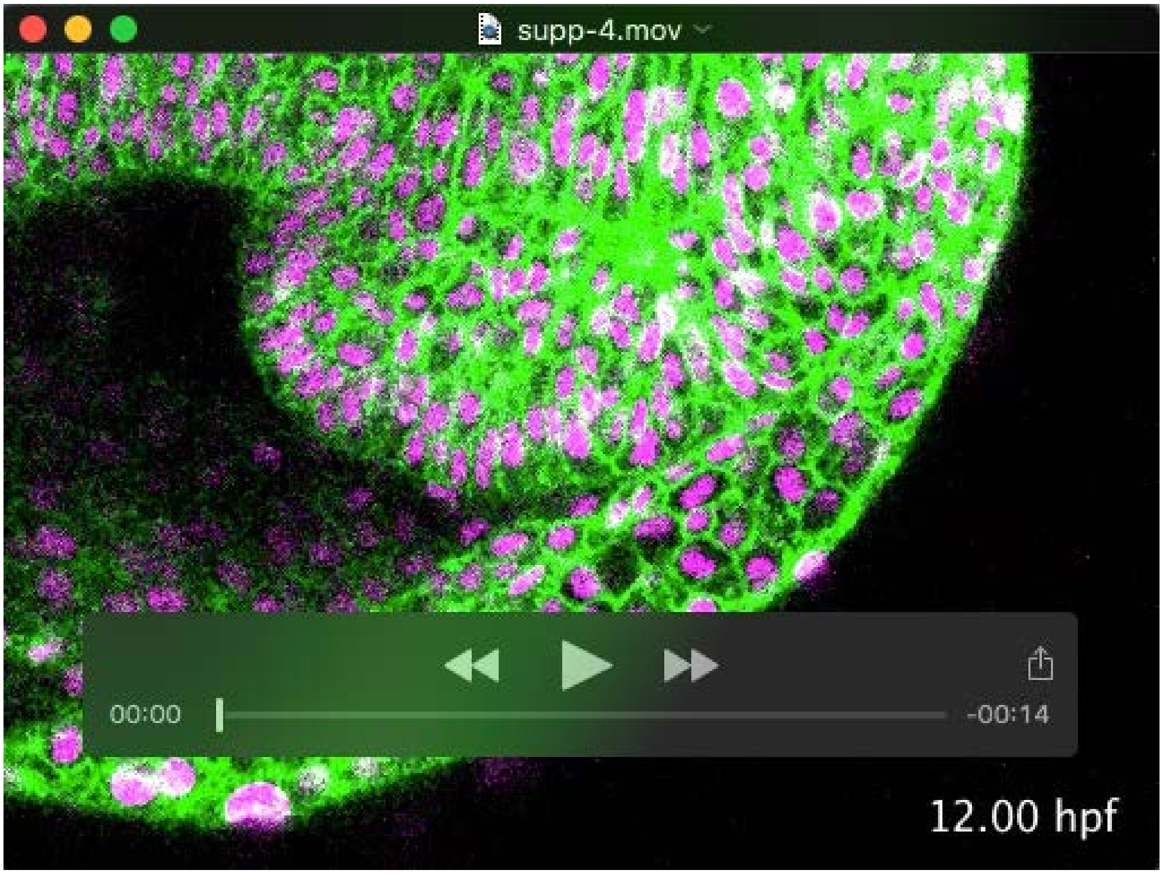

Movie 3. Timelapse of *ptch2*^{tc294z} mutant optic cup morphogenesis. Single confocal section from a 4D data set showing right optic vesicle, ~12-24 hpf. Time interval between z-stacks, 2.75 minutes. Dorsal view. Embryo labeled with EGFP-CAAX (membranes, *green*) and H2A.F/Z-mCherry (nuclei, *magenta*).

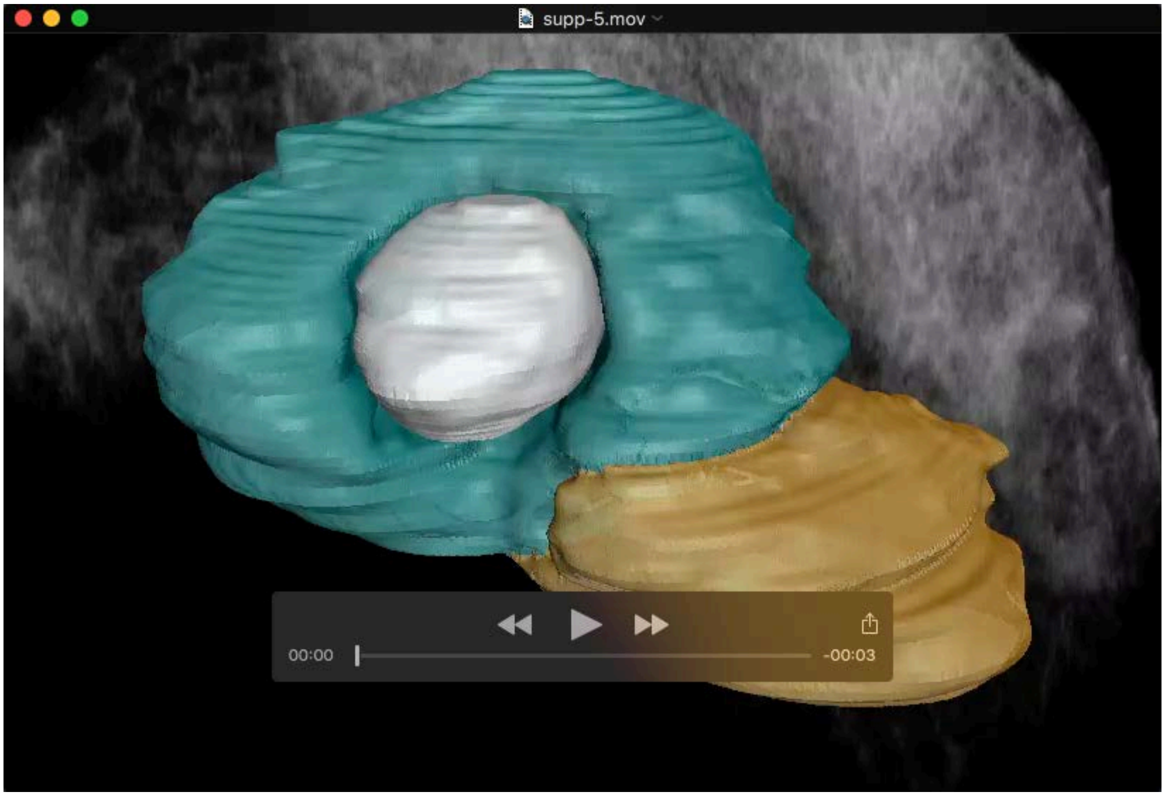

Movie 4. Segmentation of *ptch2*^{tc294z} mutant optic cup to visualize disrupted optic fissure morphology. Lateral view of 3-dimensional volume rendering of manually segmented optic cup (*teal*), lens (*gray*), and optic stalk (*gold*), 24 hpf. The optic fissure has not formed correctly; there are no obvious, closely apposed margins in the ventral optic cup.

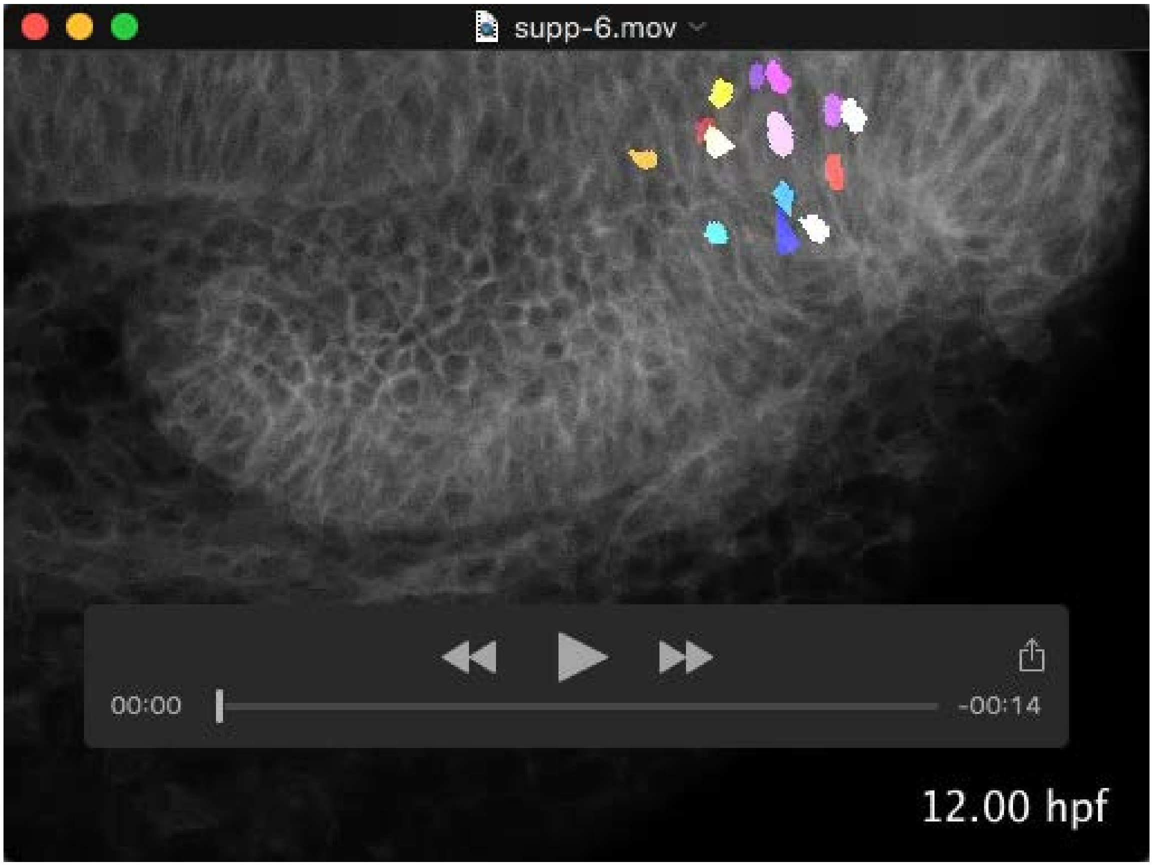

Movie 5. Wild type trajectories (~12-24 hpf): cells moving to optic fissure margins and optic stalk. Trajectories superimposed on average projection of membrane channel (*grayscale*), with temporal nuclei (*blue shades*), nasal nuclei (*red/yellow shades*), and stalk nuclei (*purple shades*). Time interval between z-stacks, 2.75 minutes. Dorsal view.

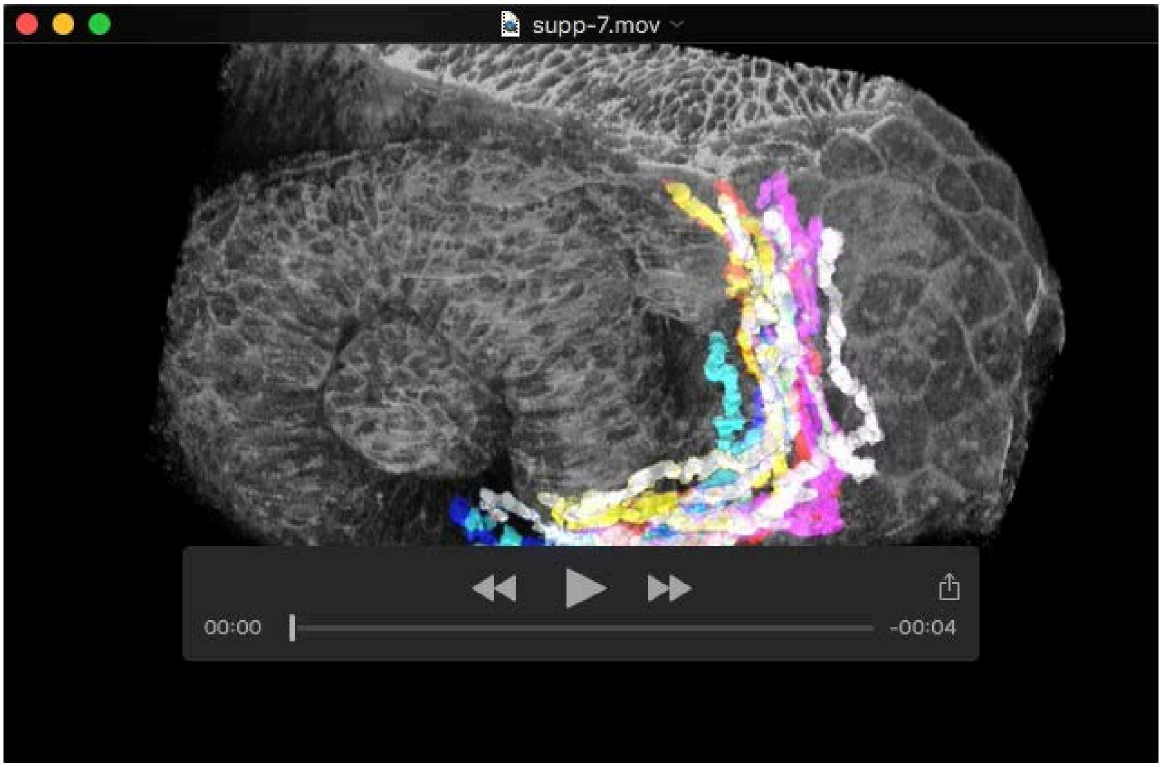

Movie 6. Rotation of wild type optic fissure and stalk trajectories. Trajectories display 3-dimensional movement.

Development • Supplementary information

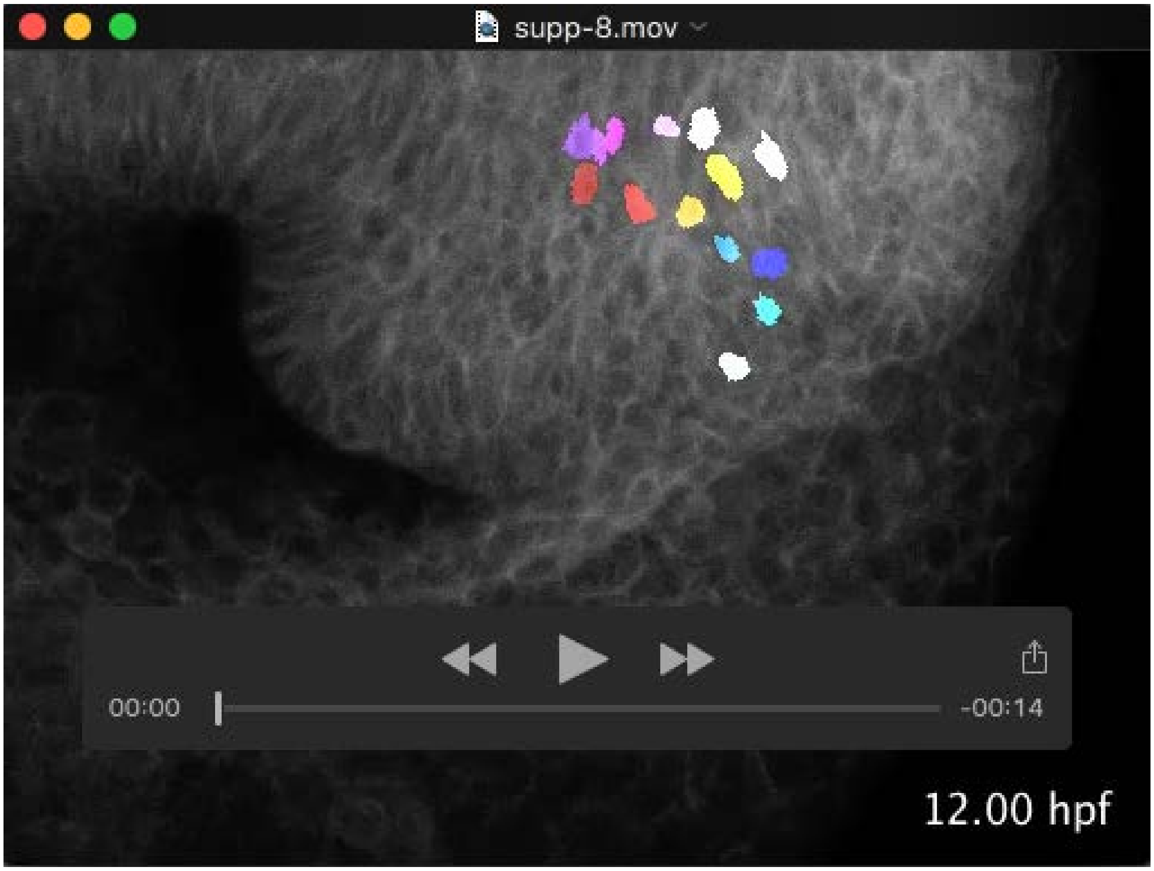

Movie 7. *ptch2^{tc294z}* mutant trajectories (~12-24 hpf): cells moving to disrupted optic fissure and optic stalk. Trajectories superimposed on average projection of membrane channel (*grayscale*), with temporal nuclei (*blue shades*), nasal nuclei (*red/yellow shades*), and stalk nuclei (*purple shades*). Time interval between z-stacks, 2.75 minutes. Dorsal view.

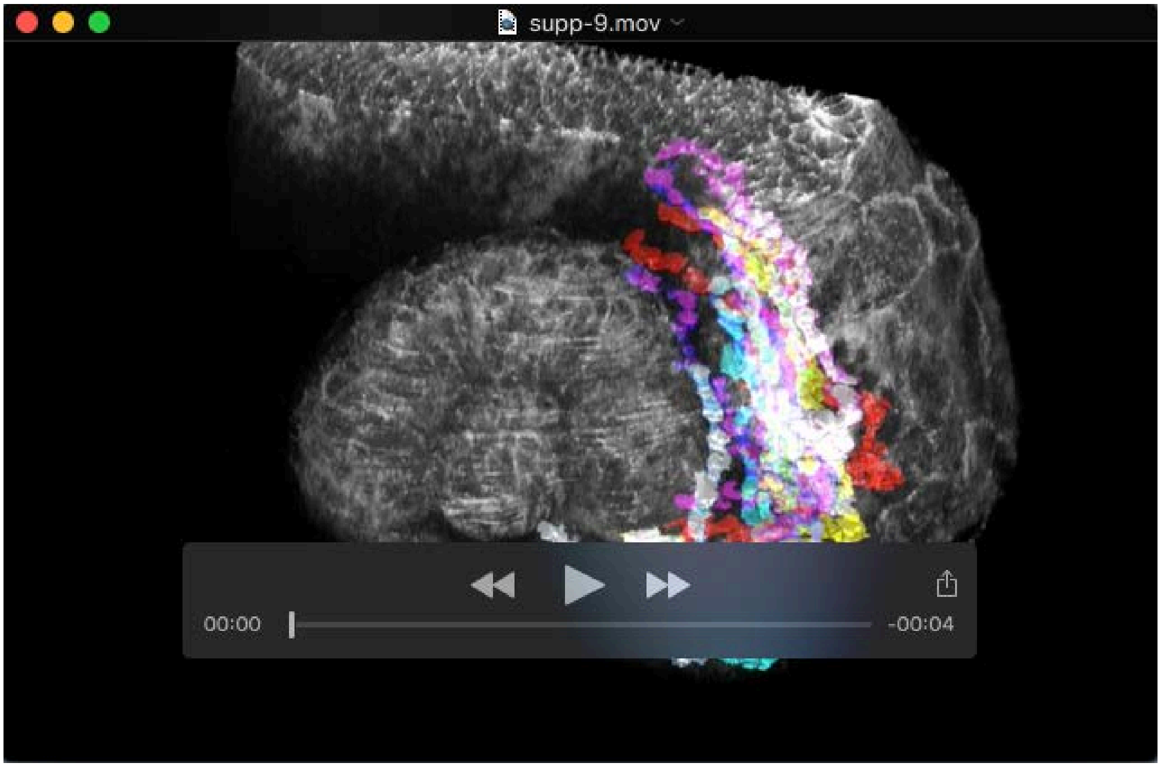

Movie 8. Rotation of *ptch2*^{tc294z} mutant trajectories from prospective optic fissure and stalk origin. Trajectories display 3-dimensional movement.

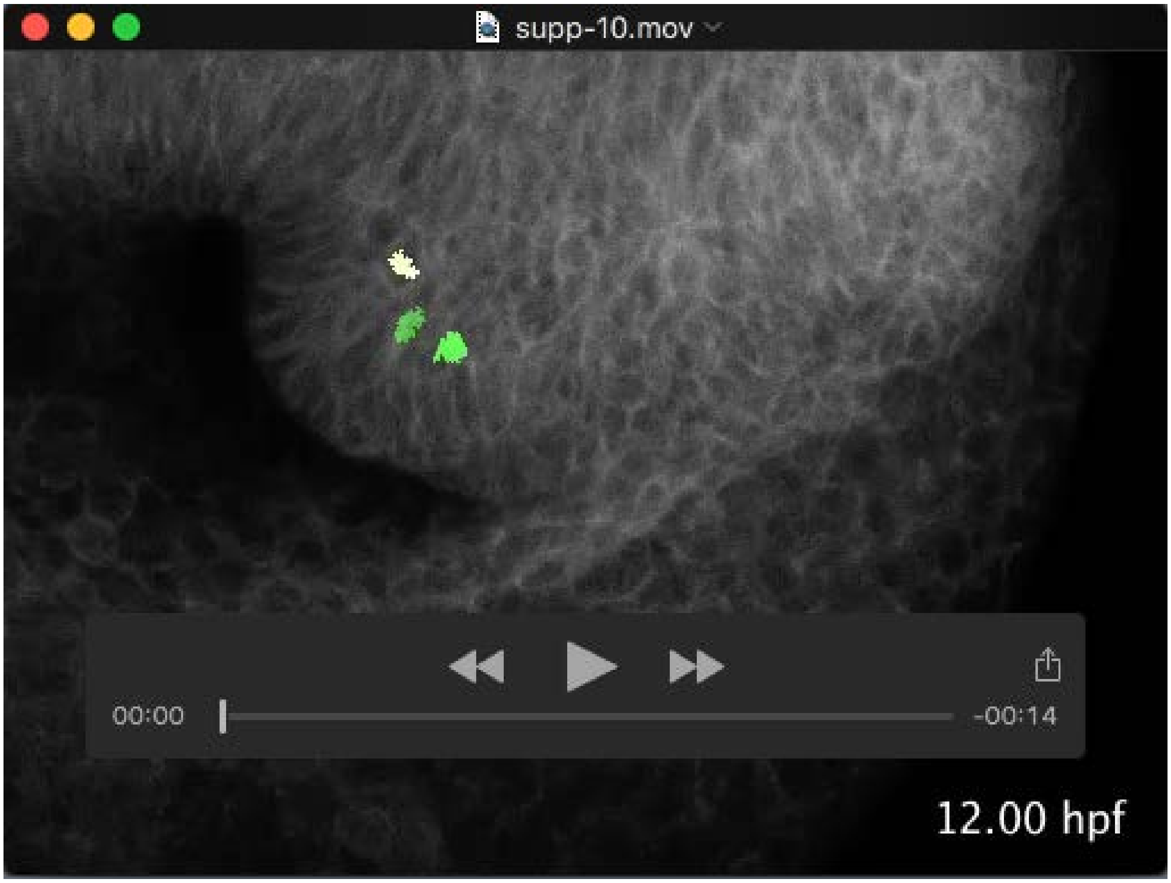

Movie 9. ***ptch2*^{tc294z} mutant trajectories (~12-24 hpf): cells moving to the aberrant temporal margin.** Trajectories superimposed on average projection of membrane channel (*grayscale*), with actual mutant temporal nuclei (*green shades*). Time interval between z-stacks, 2.75 minutes. Dorsal view.

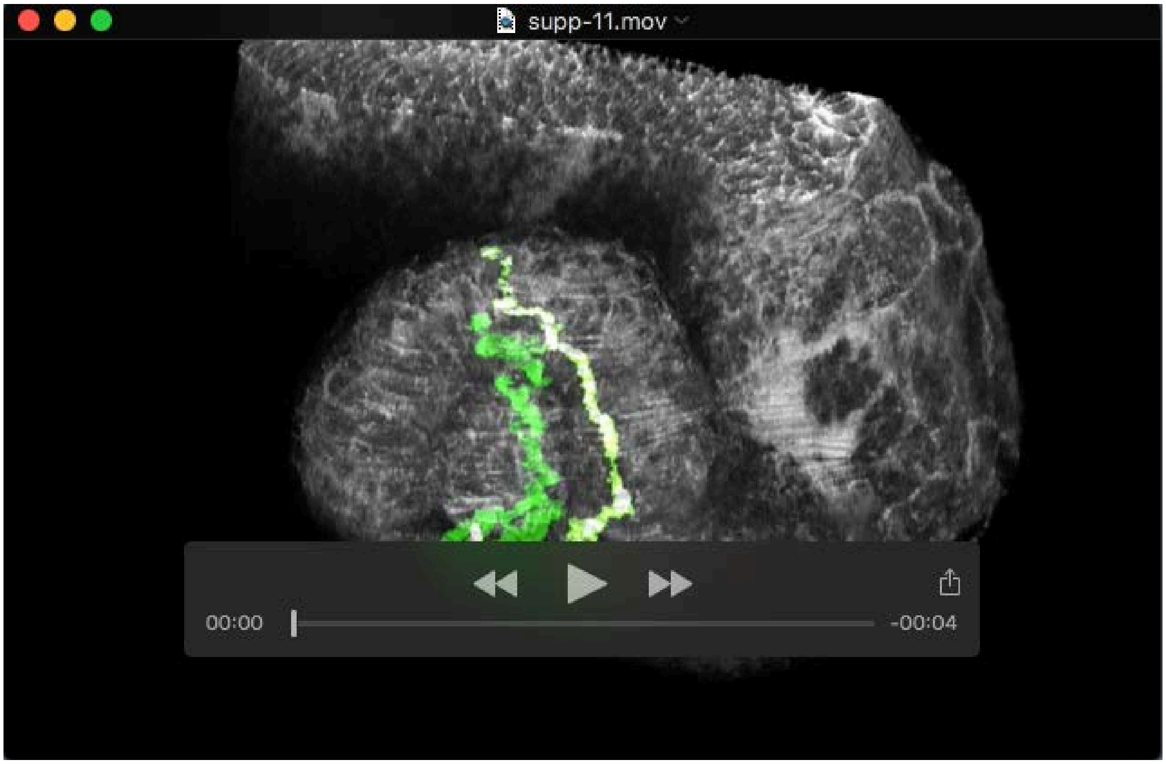

Movie 10. Rotation of *ptch2*^{tc294z} mutant trajectories of cells contributing to disrupted temporal margin. Trajectories display 3-dimensional movement.

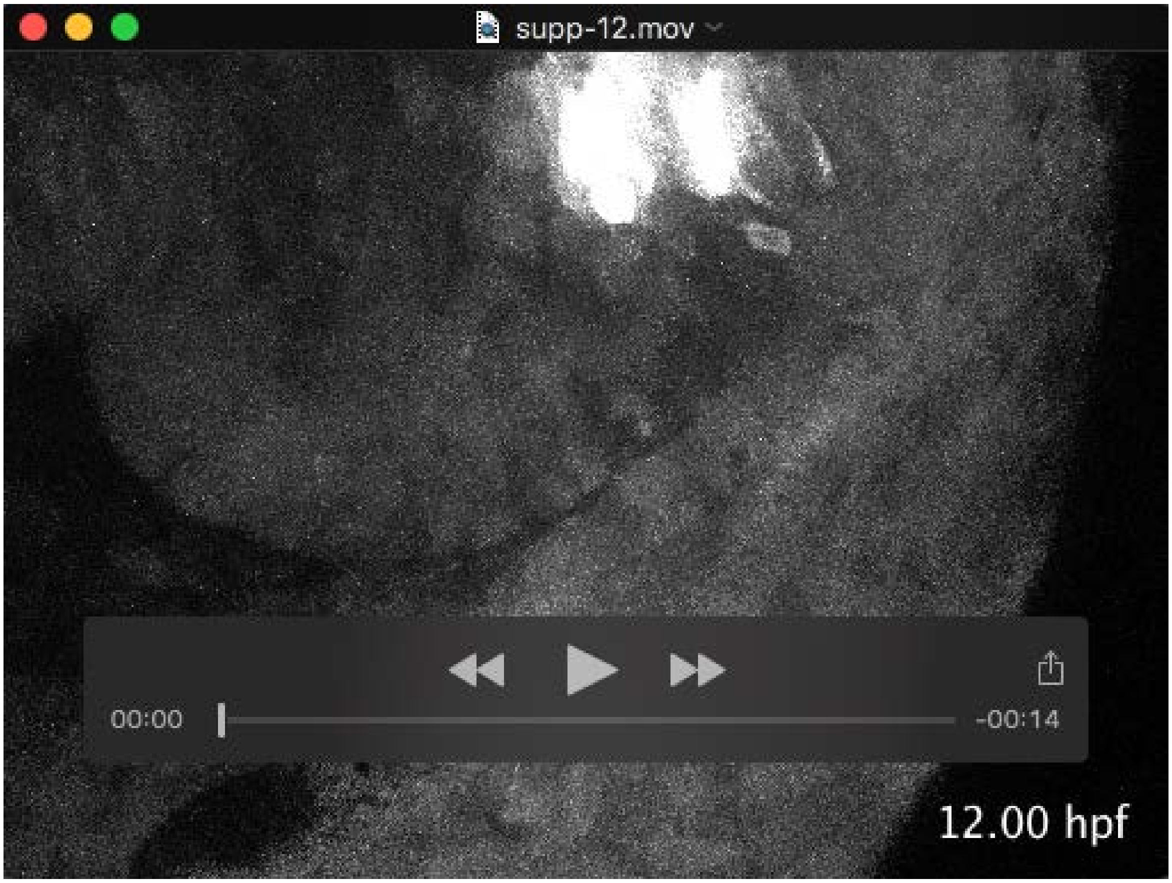

Movie 11. Timelapse of prospective nasal optic fissure cell behaviors during wild type optic cup morphogenesis (12-24 hpf). Marked cells exit the midline region, move through the prospective optic stalk domain, and into the ventronasal optic cup and nasal margin of the optic fissure. Cells maintain an elongated morphology throughout the process. Maximum intensity projection of photoconverted Kaede (grayscale) to visualize entire cell morphologies. Time interval between z-stacks, 2.75 minutes. Dorsal view.

Development • Supplementary information

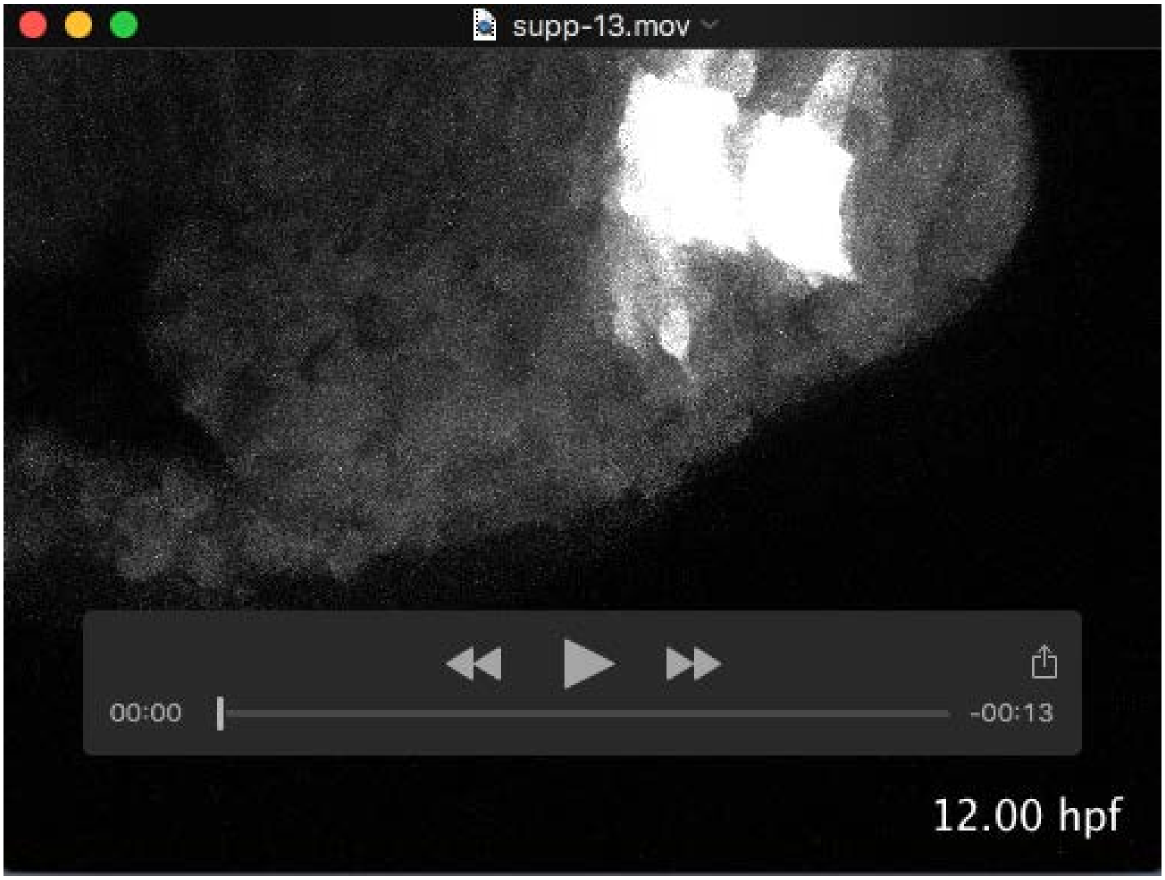

Movie 12. Timelapse of cell movements in the *ptch2^{tc294z}* mutant (12-24 hpf). Marked cells originate from a region that should contribute to the ventronasal optic cup and nasal margin of the optic fissure. Cells exit the midline region, initially exhibit an elongated morphology, but then take on an aberrant morphology and contribute to the optic stalk region. Maximum intensity projection of photoconverted Kaede (*grayscale*) to visualize entire cell morphologies. Time interval between z-stacks, 2.75 minutes. Dorsal view.

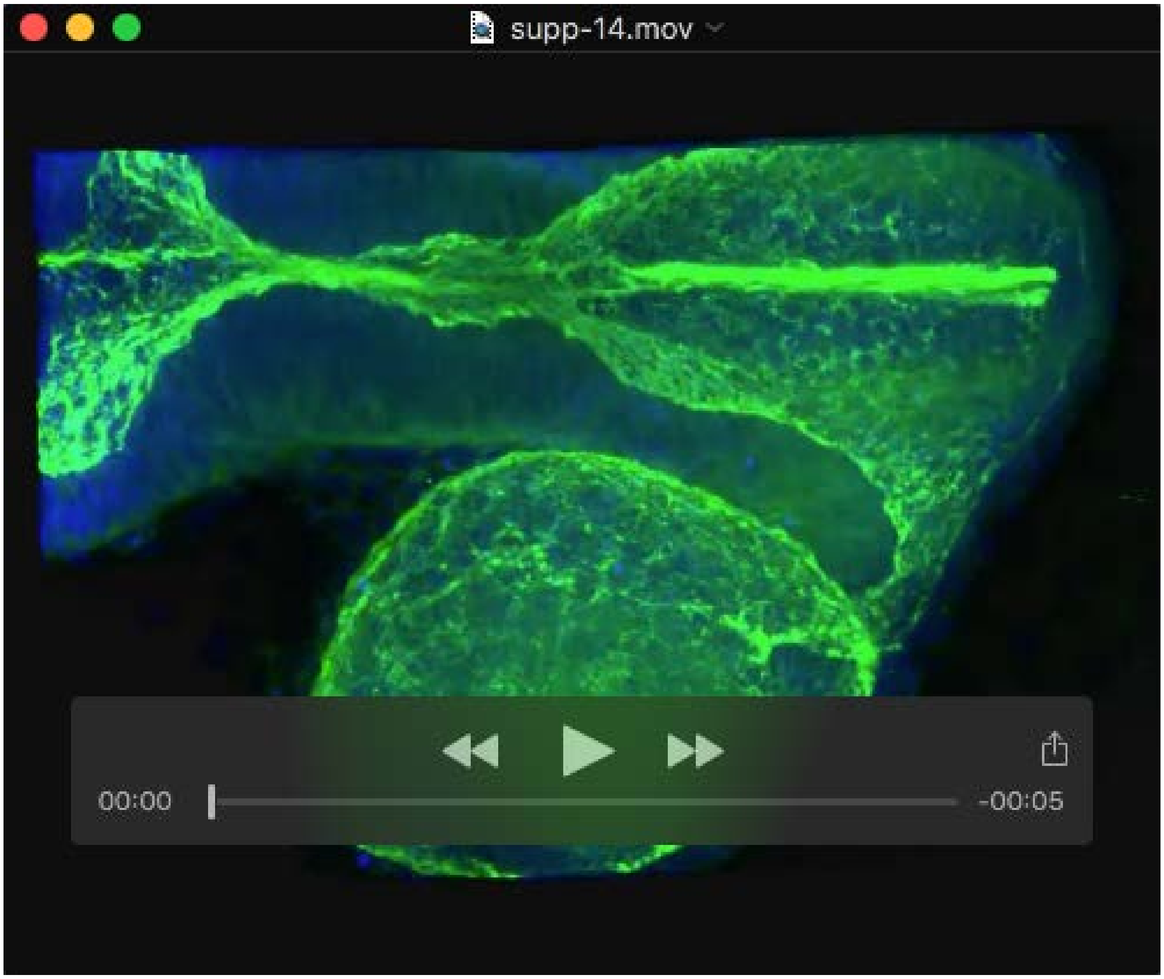

Movie 13. Rotation of wild type embryo stained for aPKC (24 hpf). A coherent apical surface is apparent in the optic cup, optic stalk, and prospective brain.

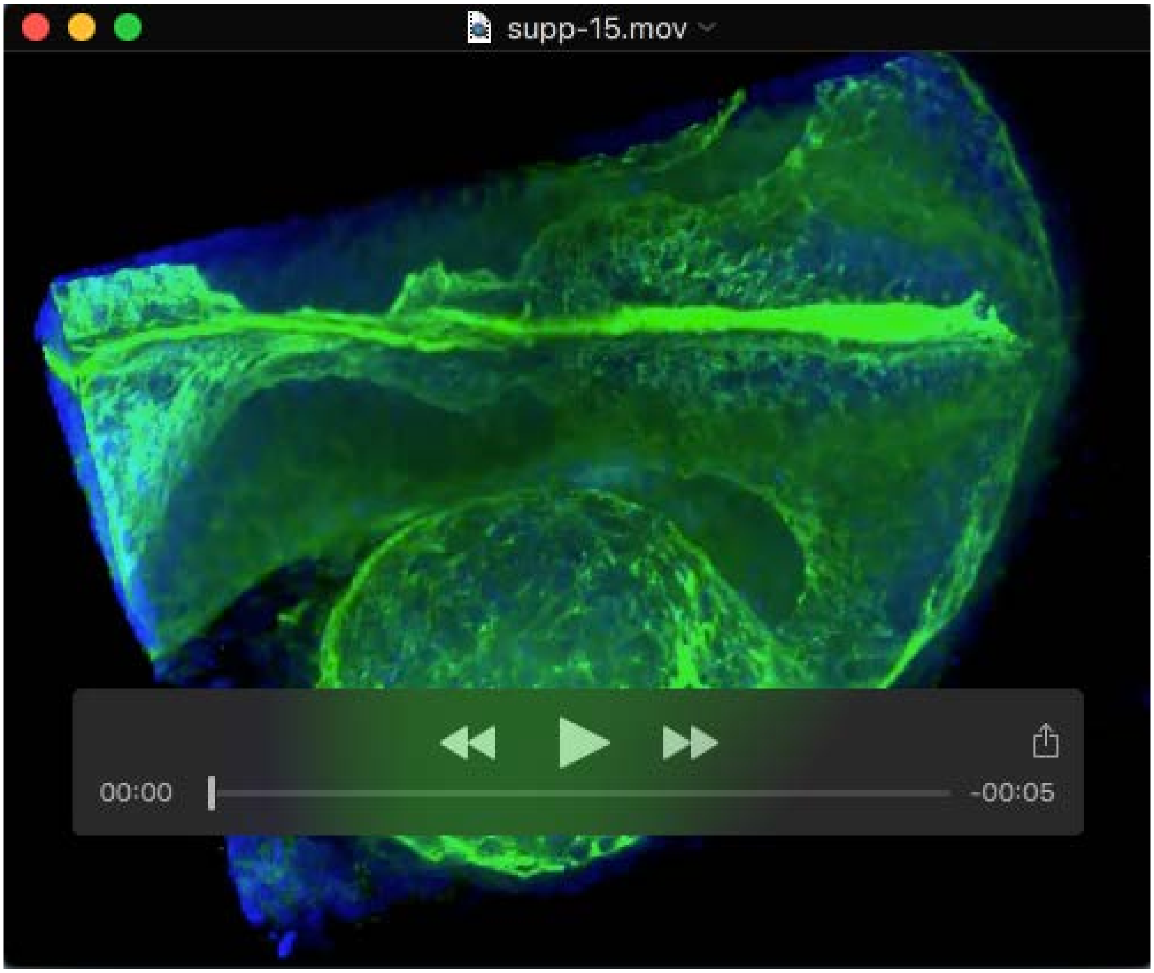

Movie 14. Rotation of *ptch2*^{tc294z} mutant embryo stained for aPKC (24 hpf). A coherent apical surface is still apparent in the optic cup, larger optic stalk, and prospective brain.

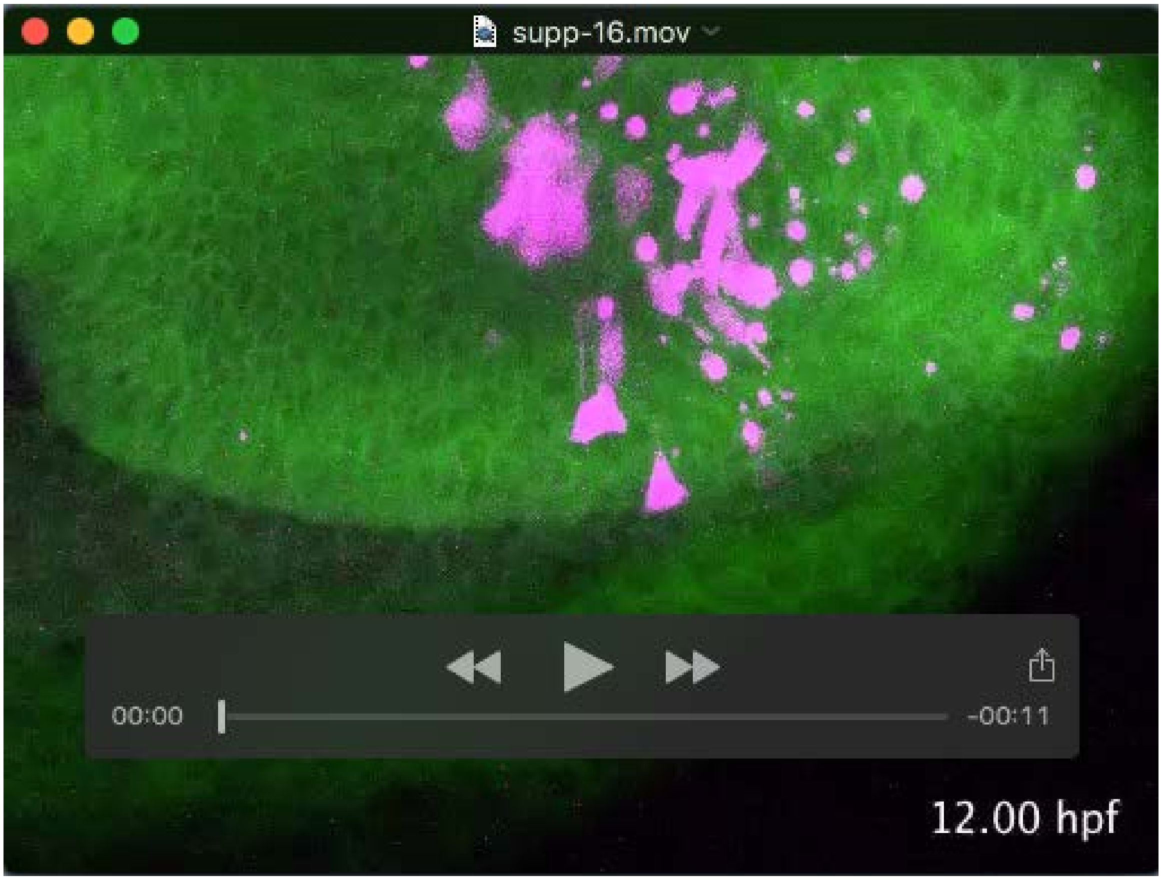

Movie 15. Timelapse of wild type donor cells in a wild type host. Donor cells (*magenta*) originating in the midline region move through the prospective optic stalk and into the optic cup. Cells maintain a bipolar morphology, and one cell contributes to the nasal optic fissure margin. Maximum intensity projection of donor cells (*magenta*, fluorescent dextran lineage tracer) superimposed on average projection of host membrane label (*green*, Tg(*bactin2*:EGFPCAAX)). Time interval between stacks, 6.25 minutes. Dorsal view.

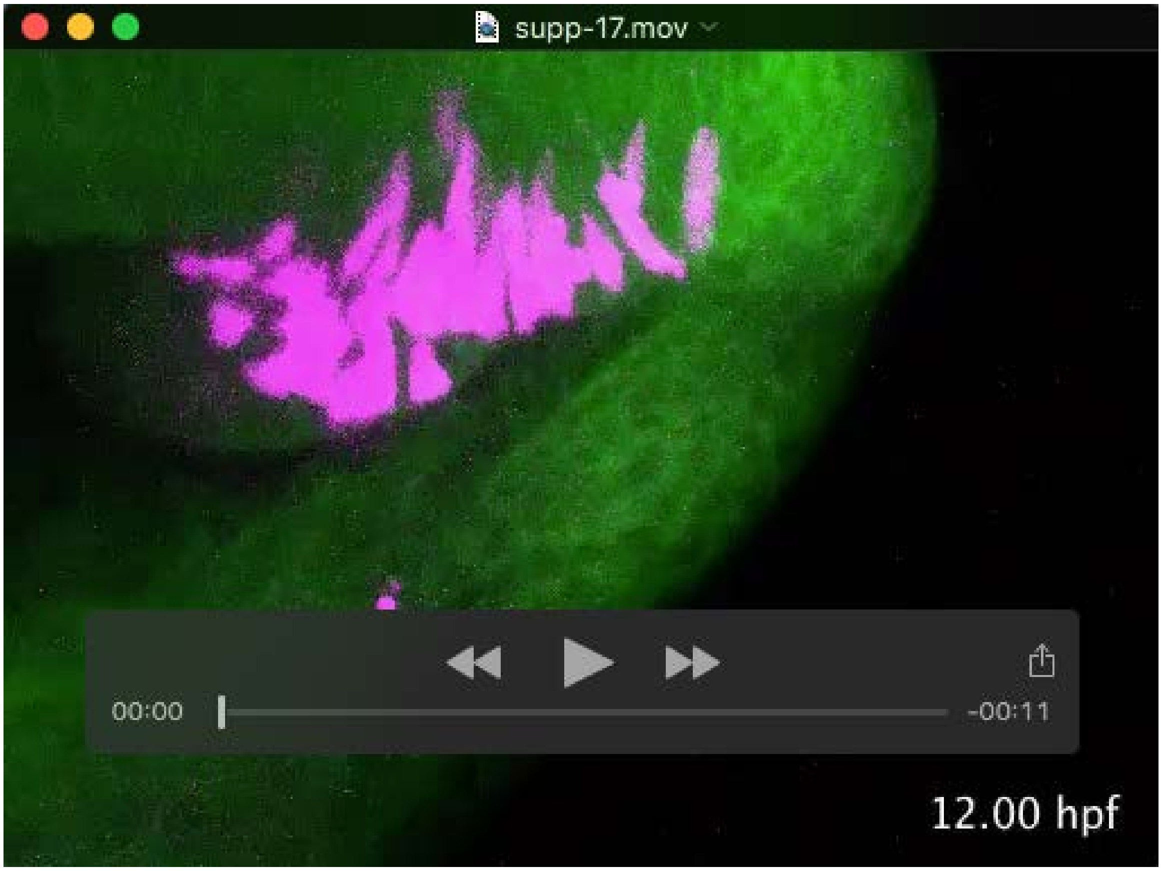

Movie 16. Timelapse of *ptch2^{tc294z}* mutant donor cells in a *ptch2^{tc294z}* mutant host. At the start of the movie, donor cells (*magenta*) are found within the optic vesicle and the midline region; we specifically follow those originating from the midline region. These cells exit the midline, initially exhibiting a bipolar morphology, however, they lose their bipolar morphology and exhibit a variety of morphologies within the optic stalk, none of which are observed in the wild type optic stalk. Maximum intensity projection of donor cells (*magenta*, fluorescent dextran lineage tracer) superimposed on average projection of host membrane label (*green*, Tg(*bactin2*:EGFPCAAX)). Time interval between stacks, 9.22 minutes. Dorsal view.

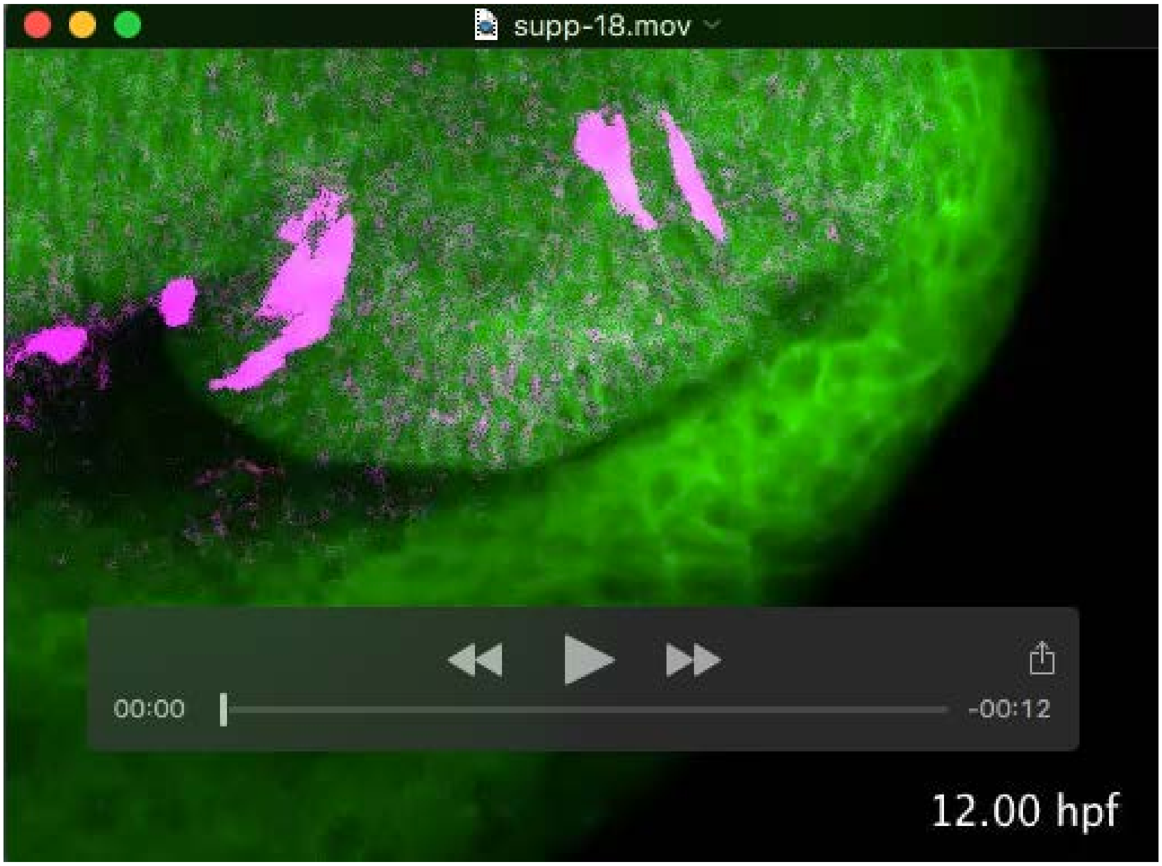

Movie 17. Timelapse of *ptch2*^{tc294z} mutant donor cells in a wild type host. Donor cells (*magenta*) originating in the midline region move through the prospective optic stalk and into the optic cup. Cells exhibit a bipolar morphology and one cell contributes to the optic fissure margin. Maximum intensity projection of donor cells (*magenta*, fluorescent dextran lineage tracer) superimposed on average projection of host membrane label (*green*, Tg(*bactin2*:EGFPCAAX)). Time interval between stacks, 8.8 minutes. Dorsal view.

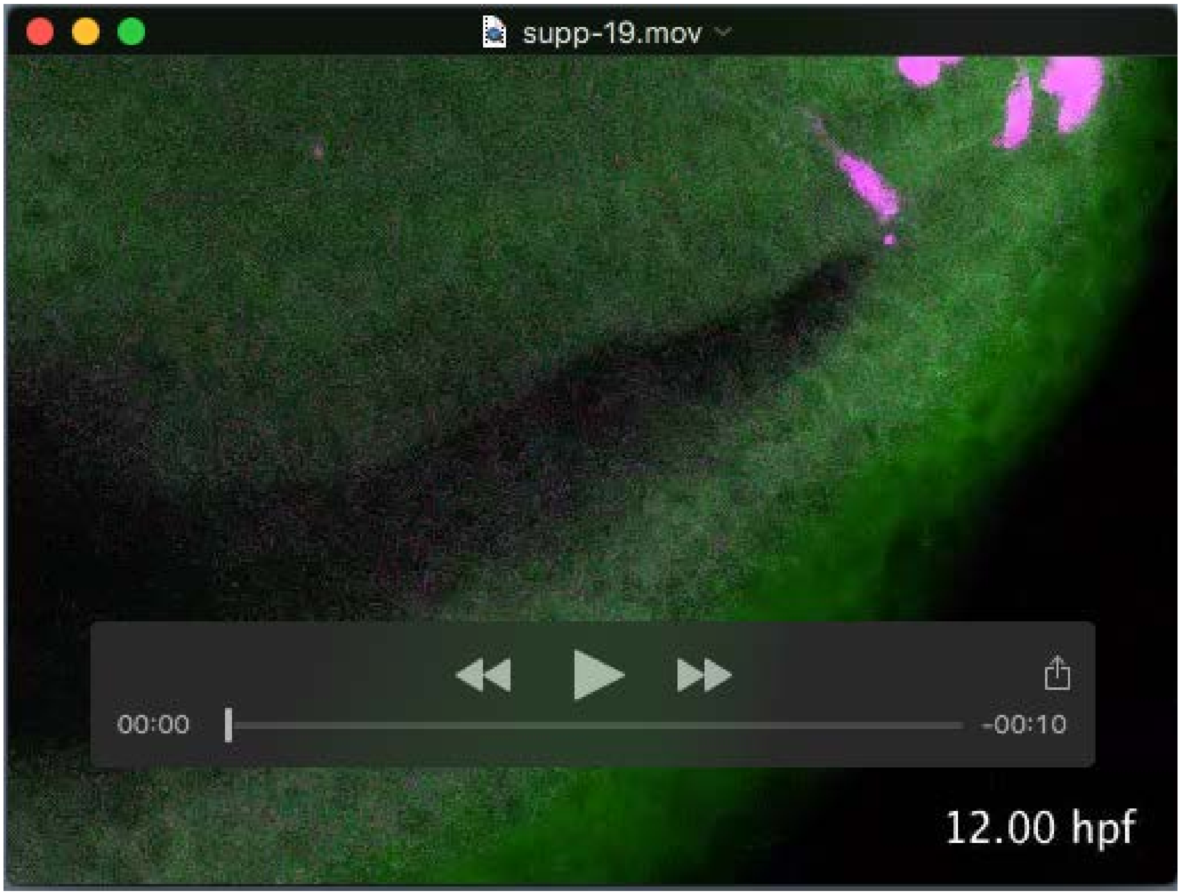

Movie 18. Timelapse of wild type donor cells in a *ptch2*^{tc294z} mutant host. Donor cells (*magenta*) originating in the midline region contribute in a large group to the central retina, or lose bipolar morphology and contribute to the optic stalk. Maximum intensity projection of donor cells (*magenta*, fluorescent dextran lineage tracer) superimposed on average projection of host membrane label (*green*, Tg(*bactin2*:EGFPCAAX)). Time interval between stacks, 7.35 minutes. Dorsal view.

Supplementary Materials and Methods: Abacus custom MATLAB code for 3D cell counting

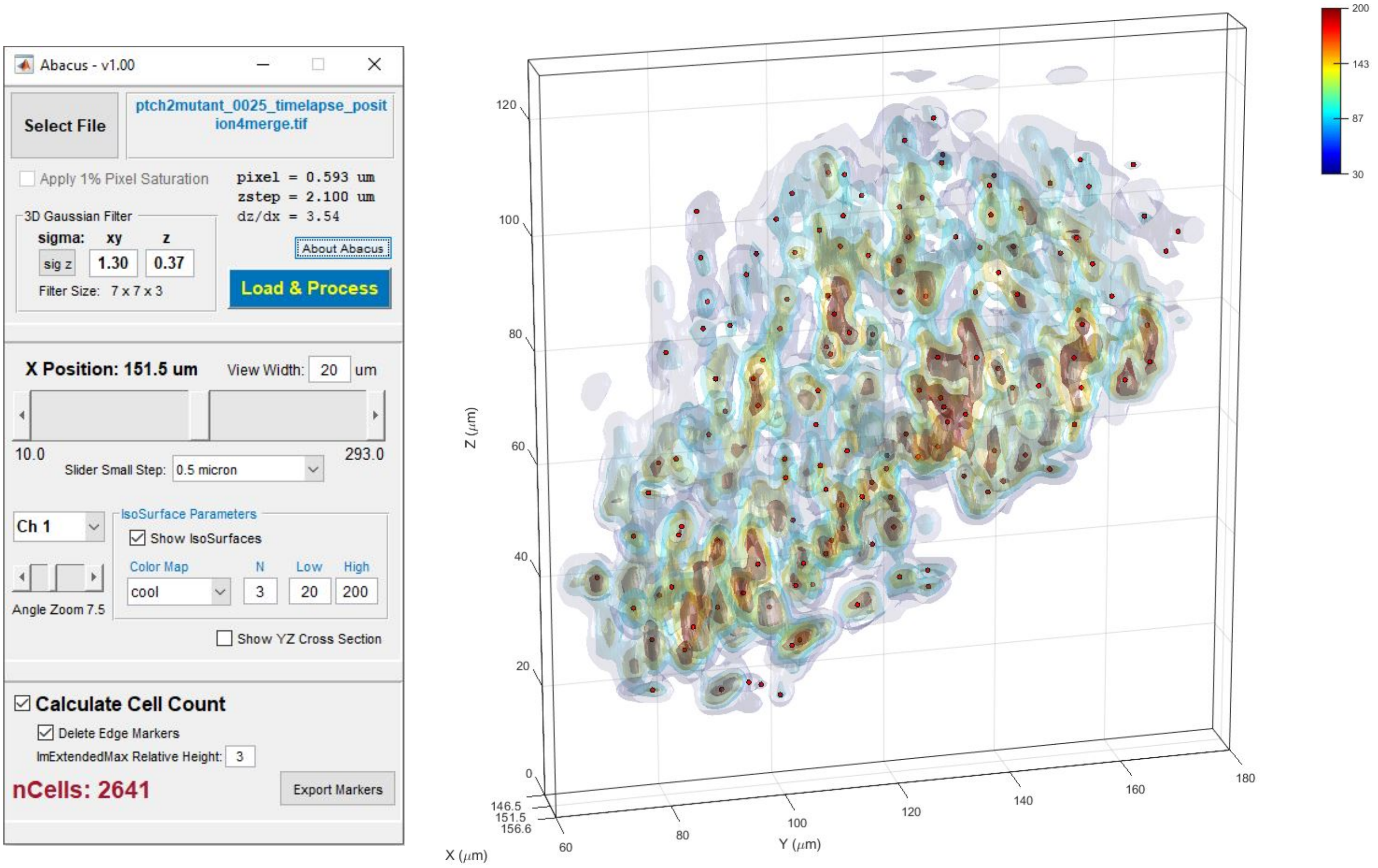

<<<< *Abacus MATLAB code below* >>>>

```
function varargout = abacus(varargin)
% ABACUS MATLAB code for abacus.fig
%      ABACUS, by itself, creates a new ABACUS or raises the existing
%      singleton*.
%
%      H = ABACUS returns the handle to a new ABACUS or the handle to
%      the existing singleton*.
%
%      ABACUS('CALLBACK',hObject,eventData,handles,...) calls the local
%      function named CALLBACK in ABACUS.M with the given input arguments.
%
%      ABACUS('Property','Value',...) creates a new ABACUS or raises the
%      existing singleton*.  Starting from the left, property value pairs are
%      applied to the GUI before abacus_OpeningFcn gets called.  An
%      unrecognized property name or invalid value makes property application
%      stop.  All inputs are passed to abacus_OpeningFcn via varargin.
%
%      *See GUI Options on GUIDE's Tools menu.  Choose "GUI allows only one
%      instance to run (singleton)".
%
% See also: GUIDE, GUIDATA, GUIHANDLES

% Edit the above text to modify the response to help abacus

% Last Modified by GUIDE v2.5 03-Aug-2018 11:39:42

% Begin initialization code - DO NOT EDIT
gui_Singleton = 1;
gui_State = struct('gui_Name',       mfilename, ...
                   'gui_Singleton',  gui_Singleton, ...
                   'gui_OpeningFcn', @abacus_OpeningFcn, ...
                   'gui_OutputFcn',  @abacus_OutputFcn, ...
                   'gui_LayoutFcn',  @abacus_LayoutFcn, ...
                   'gui_Callback',   []);
if nargin && ischar(varargin{1})
    gui_State.gui_Callback = str2func(varargin{1});
end
```

```
if nargout
    [varargout{1:nargout}] = gui_mainfcn(gui_State, varargin{:});
else
    gui_mainfcn(gui_State, varargin{:});
end
% End initialization code - DO NOT EDIT


% --- Executes just before abacus is made visible.
function abacus_OpeningFcn(hObject, eventdata, handles, varargin)

handles.output = hObject;

handles.figure1.Name = 'Abacus - v1.00';

handles.Apply_Saturation_CheckBox.Value = false;
handles.Apply_Saturation_CheckBox.Enable = 'off';
handles.Voxel_Dimension_Display.String = [{'pixel = 0.000 um'}; {'zstep = 0.000 um'}];
handles.Voxel_Dimension_Display.Enable = 'off';

set(handles.XYSigmaGaussFilt_Input,'Enable','off','String','0.00')
set(handles.ZSigmaGaussFilt_Input, 'Enable','off','String','0.00')

handles.text4.Enable = 'off';
handles.text5.Enable = 'off';
handles.text6.Enable = 'off';
handles.LoadAndProcessData_Button.Enable = 'off';

handles.Panel2.Visible = 'off';
handles.Panel3.Visible = 'off';

handles.Width_Input.String = '20';
handles.ViewWidth = 20;

handles.Channel_Selector.Value = 1;
handles.IsoSurfaces = [];
handles.fh = -1;
handles.ah = -1;

handles.ShowIsoSurfaces_Check.Value = 1;
handles.nIsoValues_Input.String = '3';
handles.nIso = 3;
handles.IsoValueLow_Input.String = '20';
handles.IsoLow = 20;
handles.IsoValueHigh_Input.String = '200';
handles.IsoHigh = 200;
handles.isoValues = round(linspace(handles.IsoHigh, handles.IsoLow, handles.nIso)');

ColorMaps = [{'parula'},{'jet'},{'cool'},...
             {'hot'},{'winter'},{'spring'},...
             {'summer'},{'bone'},{'gray'},...
             {'copper'},{'pink'},{'autumn'}];



handles.ColorMap = cool(100);
set(handles.ColorMaps_ListBox,'String',ColorMaps,'Value',3)

handles.RelativeHeight_Input.String = '3';
handles.dZdXRatio = 1;
set(handles.FilterSize_Display,'String','Filter Size:')

handles.DeleteEdgeMarkers_Check.Value = 1;

handles.SmallStepSize_ListBox.String =  [{'0.5 micron'},{'1 micron'},{'1/10 of View Width'}];
handles.SmallStepSize_ListBox.Value = 1;

guidata(hObject, handles);

% UIWAIT makes abacus wait for user response (see UIRESUME)
% uiwait(handles.figure1);


% --- Outputs from this function are returned to the command line.
function varargout = abacus_OutputFcn(hObject, eventdata, handles)

    varargout{1} = handles.output;


% --- Executes on button press in Select_File_Button.
function Select_File_Button_Callback(hObject, eventdata, handles)

    [FileName,PathName,FilterIndex] = uigetfile('*.tif');

    % Popup window to enter pixel size and z-step, but for now hard-code it.

    if ~isequal(0,FileName)

            [PixelSize, ZStep] = uiGetDataDimensions;
```

```
        if ~isequal(0,PixelSize) && ~isequal(0,ZStep)

                handles.dx = PixelSize; % microns (pixel size - same as dy)
                handles.dz = ZStep;    % microns
                handles.dZdXRatio = handles.dz/handles.dx;

                handles.ImageFileName = fullfile(PathName,FileName);
                info = imfinfo(handles.ImageFileName);
                handles.nZ = size(info,1);
                handles.ImageStackSize = [info(1).Height, info(1).Width, handles.nZ];
                set(handles.ImageName_Display,'String',FileName)

                handles.Voxel_Dimension_Display.String = [{['pixel = ' ,sprintf('%#1.3f',PixelSize) ,' um']};...
                                                          {['zstep = ' ,sprintf('%#1.3f',ZStep) ,' um']}];

                handles.Voxel_Dimension_Display.Enable = 'on';
                handles.XYSigmaGaussFilt_Input.Enable  = 'on';
                handles.ZSigmaGaussFilt_Input.Enable   = 'on';

                handles.XYSigmaGaussFilt_Input.String  = '1.20';
                %--------------------------------------------------------------------------------
                xySigma = str2double(handles.XYSigmaGaussFilt_Input.String);
                zSigma  = xySigma/handles.dZdXRatio;
                handles.ZSigmaGaussFilt_Input.String = sprintf('%0.2f', zSigma);
                    nP = num2str(2*ceil(2*xySigma)+1);
                    nZ = num2str(2*ceil(2*zSigma )+1);
                set(handles.FilterSize_Display,'String',['Filter Size:   ',nP,' x ',nP,' x ',nZ])
                %---------------------------------------------------------------------------------

                handles.text4.Enable = 'on';
                handles.text5.Enable = 'on';
                handles.text6.Enable = 'on';
                handles.LoadAndProcessData_Button.Enable = 'on';

                guidata(hObject, handles);
        end
    end


%-----------------------------------------------------------------------------------------------------
function handles = AdjustAxisTickLabels(handles)

    XLim = handles.ah.XLim;

    handles.ah.XTick = [XLim(1),...
                        handles.X_Position_Slider.Value,...
                        XLim(end)];

    handles.ah.XTickLabel = [{sprintf('%0.1f',XLim(1))};...
                             {sprintf('%0.1f',handles.X_Position_Slider.Value)};...
                             {sprintf('%0.1f',XLim(end))}];

%-----------------------------------------------------------------------------------------------------
function handles = Update_IsoSurfaces(handles)

        handles.isoValues = round(linspace(handles.IsoHigh, handles.IsoLow, handles.nIso)');

        if ~isgraphics(handles.fh)
            % Create Figure if none exists ----------------------------------------------------------
                handles.fh = figure;
                ScreenSize = get(0,'ScreenSize');  W = 1000; H = 700;
                set(handles.fh,'Color',[1 1 1],'NumberTitle','off','ToolBar','none','MenuBar','none',…
                                  'Name','Abacus Rendering Window',...
                                  'Position',[(ScreenSize(3)/2 - W/2) (ScreenSize(4)/2 - H/2) W H])

            % Create Axes in Figure, set axis limits and various params -----------------------------
                handles.ah = axes(handles.fh);
                handles.ah.Position = [0.10, 0.110, 0.7750, 0.8150];

                set(handles.ah,'CameraViewAngleMode','manual','CameraViewAngle',7.5,'BoxStyle','full',...
                               'DataAspectRatio', [1 1 1],'DataAspectRatioMode','manual',...
                               'XLim',handles.AxisSubLimits(1:2),...
                               'YLim',handles.AxisSubLimits(3:4),...
                               'ZLim',handles.AxisSubLimits(5:6));

                xlabel(handles.ah,'X (\mum)');
                ylabel(handles.ah,'Y (\mum)');
                zlabel(handles.ah,'Z (\mum)');

                view(handles.ah,60,15)
                rotate3d(handles.ah,'on')
                box(handles.ah,'on')
                grid(handles.ah,'on')
                camproj(handles.ah,'perspective')

                set(handles.ViewAngleZoom_Slider,'Min',5,'Max',12,'Value',7.5,'Enable','on','SliderStep',[0.1/7 ,1/7])
                set(handles.ViewZoom_Display,'String',['Angle Zoom ',sprintf('%0.1f',handles.ViewAngleZoom_Slider.Value)])

            % Create Colorbar -------------------------------------------------------------------------
```

```
        handles.HC = colorbar(handles.ah);
        set(handles.HC,'Position',[0.93 0.780 0.020 0.18],'LineWidth',1,...
                       'TickLength',0.04,'FontSize',8,'TickDirection','both');
        colormap(handles.ah, handles.ColorMap)
        caxis(handles.ah, [handles.IsoLow, handles.IsoHigh])
        handles.HC.Ticks = flipud(handles.isoValues)'; %linspace(handles.MagThresh, handles.MagHigh, 12);
        handles.HC.TickLabels = num2cell(flipud(handles.isoValues)); %num2cell(round(handles.HC.Ticks));

        obj = findall(handles.ah,'Type','Scatter');
        delete(obj)
        handles.ShowCellLocations_Check.Value = 0;
        handles.CellCount_Display.String = 'nCells: 0';

    end

    Alpha    = [linspace(0.3,0.1,handles.nIso)'];
    Cmap     = flipud(handles.ColorMap);
    Cind     = round(linspace(1,size(Cmap,1),handles.nIso))';
    %-----------------------------------------------------------------------------
        OldIsoSurfaces  = findall(handles.ah,'Type','Patch'); % Grab old IsoSurfaces and delete them
        OldCrossSection = findall(handles.ah,'Type','Surface');
        OldLines        = findall(handles.ah,'Type','Line');
    %-----------------------------------------------------------------------------
        handles.ah.XColor = [0.8 0.8 0.8];
        handles.ah.YColor = [0.8 0.8 0.8];
        handles.ah.ZColor = [0.8 0.8 0.8];
        drawnow
     %-----------------------------------------------------------------------------
        handles.IsoSurfaces = cell(handles.nIso,1);
        Ch = handles.Channel_Selector.Value;
     % -------------------------------------------------------------------------------
        handles.ImageSubVolume = single(squeeze(handles.RGBsmooth(:,handles.XsubIndices,:,Ch)));
     %-------------------------------------------------------------------------------

    % Create New IsoSurfaces ----------------------------------------------------
    if logical(handles.ShowIsoSurfaces_Check.Value)
        hold(handles.ah,'on')
        for n = 1:handles.nIso
            s1 = isosurface(handles.XsubMesh,...
                            handles.YsubMesh,...
                            handles.ZsubMesh,...
                            handles.ImageSubVolume,...
                            handles.isoValues(n,1));

            IsoSurfaces{n,1} = patch(handles.ah, s1);
            IsoSurfaces{n,1}.EdgeColor = 'none';
            IsoSurfaces{n,1}.FaceColor = Cmap(Cind(n,1),:);
            IsoSurfaces{n,1}.FaceAlpha = Alpha(n,1);
        end
        hold(handles.ah,'off')
    end
    % Create New YZ Cross-Section -----------------------------------------------
        handles = Update_YZ_Cross_Section(handles);
    %-----------------------------------------------------------------------------

    LightObjs = findall(handles.ah,'Type','Light');
    if length(LightObjs) < 2
        camlight(handles.ah, 90,0,'local');
        camlight(handles.ah,-90,0,'local');
    elseif length(LightObjs) > 4
        delete(LightObjs)
    end
    % Delete Old Graphics Objects ----------------------------
        delete(OldIsoSurfaces)
    %---------------------------------------------------------

    set(handles.ah,'XLim',handles.AxisSubLimits(1:2),...
                   'YLim',handles.AxisSubLimits(3:4),...
                   'ZLim',handles.AxisSubLimits(5:6));

    handles = AdjustAxisTickLabels(handles);
        handles.ah.XColor = [0.1500 0.1500 0.1500];
        handles.ah.YColor = [0.1500 0.1500 0.1500];
        handles.ah.ZColor = [0.1500 0.1500 0.1500];

    drawnow
%-------------------------------------------------------------------------------------------------------
function handles = Update_YZ_Cross_Section(handles)

        OldCrossSection = findall(handles.ah,'Type','Surface');
        OldLines        = findall(handles.ah,'Type','Line');
        delete(OldCrossSection)
        delete(OldLines)

        if logical(handles.ShowYZCrossSection_Button.Value)
            hold(handles.ah,'on')
                Xpos = handles.X_Position_Slider.Value;
                handles.YZSlice = slice(handles.ah,handles.XsubMesh,...
                                                   handles.YsubMesh,...
                                                   handles.ZsubMesh,...
```

```
                                                        handles.ImageSubVolume, Xpos, [], []);

                handles.YZSlice.FaceAlpha = 0.85;
                handles.YZSlice.EdgeColor = 'none';
                handles.YZSlice.AmbientStrength  = 1;
                handles.YZSlice.DiffuseStrength  = 0;
                handles.YZSlice.SpecularStrength = 0;
                %handles.YZSlice.CData(handles.YZSlice.CData < handles.IsoLow) = NaN;
        end

        caxis(handles.ah, [handles.IsoLow, handles.IsoHigh])
        hold(handles.ah,'off')


%----------------------------------------------------------------------------------------------------
function handles = UpdateCellCount(handles)

        Ch = handles.Channel_Selector.Value;
        Volume = squeeze(handles.RGBsmooth(:,:,:,Ch));
        H = str2double(handles.RelativeHeight_Input.String);
        M3D = imextendedmax(Volume,H);
        [L, NUM] = bwlabeln(M3D);
        stats = regionprops(L,'Centroid'); % Grab centers of 3d-objects
        C = struct2cell(stats);
        C = C';
        A = cell2mat(C);
        % Filter Cells by thresholding by brightness ----------------------------------------
            Ind = floor(A);
            Magnitude = zeros(length(A),1);
            for k = 1:length(A)
                Magnitude(k,1) = Volume(Ind(k,2),Ind(k,1),Ind(k,3));
            end
            A = A(Magnitude >= handles.IsoLow,:);  % Positions are in units of pixel indices
        %------------------------------------------------------------------------------------
        if logical(handles.DeleteEdgeMarkers_Check.Value)
            w = 1;
            [ii,jj,kk] = size(L);
            ind1 = find( A(:,1) <= (min(A(:,1)) + w)  |  A(:,1) >= (max(A(:,1)) - w) );
            ind2 = find( A(:,2) <= (min(A(:,2)) + w)  |  A(:,2) >= (max(A(:,2)) - w) );
            ind3 = find( A(:,3) <= (min(A(:,3)) + w)  |  A(:,3) >= (max(A(:,3)) - w) );
            idx = [ind1; ind2; ind3];
            A(idx,:) = [];
        end
        %------------------------------------------------------------------------------------
            handles.CellCountLocationsVoxels = [A(:,1), A(:,2), A(:,3)];
            X = handles.dx*A(:,1) - handles.dx;
            Y = handles.dx*A(:,2) - handles.dx;
            Z = handles.dz*A(:,3) - handles.dz;
            handles.CellCountLocationsMicrons = [X, Y, Z];
        %------------------------------------------------------------------------------------
            obj = findall(handles.ah,'Type','Scatter');
            delete(obj)
        %------------------------------------------------------------------------------------
            handles.CellCount = size(A,1);
            set(handles.CellCount_Display,'String',['nCells: ' num2str(handles.CellCount)])
            hold(handles.ah,'on')
                scatter3(handles.ah, X, Y, Z, 14,...
                         'MarkerEdgeColor', 'k',...
                         'MarkerFaceColor', [1, 0, 0]);
            hold(handles.ah,'off')

%----------------------------------------------------------------------------------------------------
function FP = WaitToProcessDialogue

        ScreenSize = get(0,'ScreenSize');
        FP = figure;
        W = 260;
        H = 75;
        set(FP,'ToolBar','none','MenuBar','none',...
              'NumberTitle','off','Name','Abacus - wait',...
              'Position',[(ScreenSize(3)/2 - W/2) (ScreenSize(4)/2 - H/2) W H])
        a = gca;
        set(a,'Visible','off','XLim',[0 1],'YLim',[0 1])
        text(a,0,0.5,[{'Counting Cells'}],'Color',[0.4 0.4 0.4],...
            'FontSize',16,'FontName','Monospaced','FontWeight','bold')
        drawnow

%----------------------------------------------------------------------------------------------------
function Apply_Saturation_CheckBox_Callback(hObject, eventdata, handles)



%----------------------------------------------------------------------------------------------------
function XYSigmaGaussFilt_Input_Callback(hObject, eventdata, handles)

        xySigma = str2double(handles.XYSigmaGaussFilt_Input.String);

        if ~isnan(xySigma)
            if xySigma < 0.01 | xySigma > 10
                xySigma = 0.5;
```

Development • Supplementary information

```
            end
            set(handles.XYSigmaGaussFilt_Input,'String',sprintf('%0.2f',xySigma))
            zSigma  = str2double(handles.ZSigmaGaussFilt_Input.String);
            nP = num2str(2*ceil(2*xySigma)+1);
            nZ = num2str(2*ceil(2*zSigma )+1);
            set(handles.FilterSize_Display,'String',['Filter Size:   ',nP,' x ',nP,' x ',nZ])
        end

        guidata(hObject, handles);

%----------------------------------------------------------------------------------------------------
function XYSigmaGaussFilt_Input_CreateFcn(hObject, eventdata, handles)

    if ispc && isequal(get(hObject,'BackgroundColor'), get(0,'defaultUicontrolBackgroundColor'))
        set(hObject,'BackgroundColor','white');
    end

%----------------------------------------------------------------------------------------------------
function Xsize_BoxFilt_Input_Callback(hObject, eventdata, handles)



%----------------------------------------------------------------------------------------------------
function Xsize_BoxFilt_Input_CreateFcn(hObject, eventdata, handles)

    if ispc && isequal(get(hObject,'BackgroundColor'), get(0,'defaultUicontrolBackgroundColor'))
        set(hObject,'BackgroundColor','white');
    end


%----------------------------------------------------------------------------------------------------
function ZSigmaGaussFilt_Input_Callback(hObject, eventdata, handles)

        zSigma = str2double(handles.ZSigmaGaussFilt_Input.String);
        xySigma  = str2double(handles.XYSigmaGaussFilt_Input.String);

        if ~isnan(zSigma)
            if zSigma < 0.01 | zSigma > 10
                zSigma = xySigma/handles.dZdXRatio;;
            end
            set(handles.ZSigmaGaussFilt_Input,'String',sprintf('%0.2f',zSigma))
            nP = num2str( 2*ceil(2*xySigma) + 1 );
            nZ = num2str( 2*ceil(2*zSigma ) + 1 );
            set(handles.FilterSize_Display,'String',['Filter Size:   ',nP,' x ',nP,' x ',nZ])
        end

        guidata(hObject, handles);


%----------------------------------------------------------------------------------------------------
function ZSigmaGaussFilt_Input_CreateFcn(hObject, eventdata, handles)

        if ispc && isequal(get(hObject,'BackgroundColor'), get(0,'defaultUicontrolBackgroundColor'))
            set(hObject,'BackgroundColor','white');
        end


%----------------------------------------------------------------------------------------------------
function LoadAndProcessData_Button_Callback(hObject, eventdata, handles)

            ImageSaturate = @(Im,percent) imadjust(Im,stretchlim(Im,[percent/100, (100-percent)/100]));

            % Read-in and Process images ---------------------------------------------------------
                handles.RGBraw = zeros([handles.ImageStackSize,3], 'uint8');

                h = waitbar(0,'Loading Data');

                for z = 1:handles.nZ
                    waitbar(z/handles.nZ,h);
                    drawnow
                    IM = imread(handles.ImageFileName,z);
                    if isequal(size(IM,3),3); % If Image is RGB------------------------------
                        if logical(handles.Apply_Saturation_CheckBox.Value)
                            handles.RGBraw(:,:,z,1) = ImageSaturate(squeeze(IM(:,:,1)), 1);
                            handles.RGBraw(:,:,z,2) = ImageSaturate(squeeze(IM(:,:,2)), 1);
                            handles.RGBraw(:,:,z,3) = ImageSaturate(squeeze(IM(:,:,3)), 1);
                        else
                            handles.RGBraw(:,:,z,1) = squeeze(IM(:,:,1));
                            handles.RGBraw(:,:,z,2) = squeeze(IM(:,:,2));
                            handles.RGBraw(:,:,z,3) = squeeze(IM(:,:,3));
                        end
                        handles.Channel_Selector.Enable = 'on';
                        handles.Channel_Selector.Value = 1;
                    else % If image is grayscale --------------------------------------------
                        if logical(handles.Apply_Saturation_CheckBox.Value)
                            handles.RGBraw(:,:,z,1) = ImageSaturate(IM, 1);
                        else
                            handles.RGBraw(:,:,z,1) = squeeze(IM);
                        end
                        handles.Channel_Selector.Enable = 'off';
```

```
                %handles.Channel_Selector.Value = 1;
                %--------------------------------------------------------------------
            end
        end

    % Apply Gaussian smoothing ------------------------------------------------------------------------------------

        zSigma = str2double(handles.ZSigmaGaussFilt_Input.String);
        xySigma  = str2double(handles.XYSigmaGaussFilt_Input.String);

        waitbar(1,h,['Applying 3D Gaussian Smoothing'])

        handles.RGBsmooth = zeros([handles.ImageStackSize,3], 'uint8');

        if isequal(size(IM,3),3)
            handles.RGBsmooth(:,:,:,1) = imgaussfilt3(handles.RGBraw(:,:,:,1),...
                                             [xySigma,xySigma,zSigma], 'padding', 'circular');
            handles.RGBsmooth(:,:,:,2) = imgaussfilt3(handles.RGBraw(:,:,:,2),...
                                             [xySigma,xySigma,zSigma], 'padding', 'circular');
            handles.RGBsmooth(:,:,:,3) = imgaussfilt3(handles.RGBraw(:,:,:,3),...
                                             [xySigma,xySigma,zSigma], 'padding', 'circular');
        else
            handles.RGBsmooth(:,:,:,1) = imgaussfilt3(handles.RGBraw(:,:,:,1),...
                                             [xySigma,xySigma,zSigma], 'padding', 'circular');
        end

        pause(0.25)
        try
            delete(h);
        end
    %--------------------------------------------------------------------------------------------------------------

        handles.X_Axis_Vector = (handles.dx*[0:handles.ImageStackSize(2)-1])';
        handles.Y_Axis_Vector = (handles.dx*[0:handles.ImageStackSize(1)-1])';
        handles.Z_Axis_Vector = (handles.dz*[0:handles.nZ-1])';

    % Setup Slider and Value Display-----------------------------------------------------------------------------

        handles.PositionSliderMinValue_Display.String = sprintf('%#0.1f',handles.ViewWidth/2);
        handles.PositionSliderMaxValue_Display.String = ...
                                     sprintf('%#0.1f',handles.X_Axis_Vector(end)-handles.ViewWidth/2);

        Range = handles.X_Axis_Vector(end)-handles.ViewWidth;
        %SmallStep = 0.1*handles.ViewWidth/Range;
        SmallStep = 0.5/Range;
        BigStep = handles.ViewWidth/Range;

        set(handles.X_Position_Slider,'Value',handles.X_Axis_Vector(end)/2,'SliderStep',[SmallStep,BigStep],...
                               'Min',handles.ViewWidth/2,'Max',handles.X_Axis_Vector(end)-handles.ViewWidth/2)

        set(handles.SliderBarPosition_Display,'String',['X Position: ',...
                                            sprintf('%0.1f',handles.X_Axis_Vector(end)/2),' um'])

    % Create MESH grid -------------------------------------------------------------------------------

        [handles.Xmesh, handles.Ymesh, handles.Zmesh] = meshgrid(handles.X_Axis_Vector,...
                                                        handles.Y_Axis_Vector,...
                                                        handles.Z_Axis_Vector);

    % Calculate Sub-Mesh, Sub-Image, and Sub-Indices --------------------------------------------------
        handles = Update_SubVolume_And_AxisLimits(handles);
        handles = Update_IsoSurfaces(handles);

        obj = findall(handles.ah,'Type','Scatter');    % Find old cell markers if they exist and delete them
        delete(obj)
        handles.CellCount_Display.String = 'nCells: 0';
    %-----------------------------------------------------------------------------------------------
        handles.Panel2.Visible = 'on';
        handles.Panel3.Visible = 'on';
        % If Box is checked, recalculate cell count --------------
        if logical(handles.ShowCellLocations_Check.Value)
            FP = WaitToProcessDialogue; drawnow;
            handles = UpdateCellCount(handles);
            delete(FP)
        end
    %----------------------------------------------------------

        guidata(hObject, handles);

%-----------------------------------------------------------------------------------------------------
function handles = Update_SubVolume_And_AxisLimits(handles)

         Xpos = handles.X_Position_Slider.Value;
         Width = handles.ViewWidth;
         X_Axis_Vector = handles.X_Axis_Vector;

         handles.XsubIndices = find( abs(X_Axis_Vector - Xpos) <= (Width/2 + handles.dx/2) );

         handles.XsubMesh = handles.Xmesh( :, handles.XsubIndices, : );
         handles.YsubMesh = handles.Ymesh( :, handles.XsubIndices, : );
```

```
         handles.ZsubMesh = handles.Zmesh( :, handles.XsubIndices, : );

         handles.AxisSubLimits = [handles.XsubMesh(1,1,1),handles.XsubMesh(1,end,1),...
                                  handles.YsubMesh(1,1,1),handles.YsubMesh(end,1,1),...
                                  handles.ZsubMesh(1,1,1),handles.ZsubMesh(1,1,end)];

%---------------------------------------------------------------------------------------------------------
function X_Position_Slider_Callback(hObject, eventdata, handles)

        set(handles.SliderBarPosition_Display, 'String', ['X Position: ',...
                    sprintf('%0.2f',handles.X_Position_Slider.Value),' um'])

        handles = Update_SubVolume_And_AxisLimits(handles);
        handles = Update_IsoSurfaces(handles);

        guidata(hObject, handles);

%---------------------------------------------------------------------------------------------------------
function X_Position_Slider_CreateFcn(hObject, eventdata, handles)

        if isequal(get(hObject,'BackgroundColor'),...
                   get(0,'defaultUicontrolBackgroundColor'))
                   set(hObject,'BackgroundColor',[.9 .9 .9]);
        end

%---------------------------------------------------------------------------------------------------------
function Width_Input_Callback(hObject, eventdata, handles)

        val = round(str2num(handles.Width_Input.String));

        if isempty(val)
            handles.Width_Input.String = handles.ViewWidth;
        else
            handles.ViewWidth = val;
            handles.Width_Input.String = num2str(val);

            Range = handles.X_Axis_Vector(end) - handles.ViewWidth;

            V = handles.SmallStepSize_ListBox.Value;
            Range = handles.X_Axis_Vector(end)-handles.ViewWidth;
            BigStep = handles.ViewWidth/Range;

            switch V
                case 1
                        SmallStep = 0.5/Range;
                case 2
                        SmallStep = 1/Range;
                case 3
                        SmallStep = 0.1*BigStep;
            end

            set(handles.X_Position_Slider,'SliderStep',[SmallStep,BigStep])

            handles.PositionSliderMinValue_Display.String = sprintf('%#0.1f', handles.ViewWidth/2);
            handles.PositionSliderMaxValue_Display.String = ...
                                                    sprintf('%#0.1f', handles.X_Axis_Vector(end) - handles.ViewWidth/2);

            set(handles.X_Position_Slider, 'Value', handles.X_Axis_Vector(end)/2,...
                                           'SliderStep', [SmallStep,BigStep],...
                                           'Min', handles.ViewWidth/2,...
                                           'Max', handles.X_Axis_Vector(end) - handles.ViewWidth/2)

            set(handles.SliderBarPosition_Display, 'String',...
                                                  ['X Position: ', sprintf('%0.1f', handles.X_Axis_Vector(end)/2),' um'])
            %---------------------------------------------------------------------------------------------------------
            handles = Update_SubVolume_And_AxisLimits(handles);
            handles = Update_IsoSurfaces(handles);
        end

        guidata(hObject, handles);

%---------------------------------------------------------------------------------------------------------
function Width_Input_CreateFcn(hObject, eventdata, handles)

        if ispc && isequal(get(hObject,'BackgroundColor'), get(0,'defaultUicontrolBackgroundColor'))
            set(hObject,'BackgroundColor','white');
        end

%---------------------------------------------------------------------------------------------------------
function [PixelSize, ZStep] = uiGetDataDimensions

        global PixelSize ZStep

        R = groot;
        SS = R.ScreenSize(1,3:4);

        PixelSize = 0;
        ZStep = 0;

        d = dialog('NumberTitle','off','Position',[SS(1)/2-160, SS(2)/2-100, 320, 250],'Name','Abacus - Dimensions Input');
```

```
        txt1  = uicontrol('Parent',d,'Style','text','Position',[27 200 185 21],'Units','pixels',...
                          'String','Enter Data Dimensions:','FontSize',12,'FontWeight','bold','HorizontalAlignment','left');

        txt2  = uicontrol('Parent',d,'Style','text','Position',[49 153 127 18],'Units','pixels',...
                          'String','Pixel Width (um):','FontSize',11,'FontWeight','normal','HorizontalAlignment','right');

        txt3  = uicontrol('Parent',d,'Style','text','Position',[49 112 127 18],'Units','pixels',...
                          'String','Z-Step (um):','FontSize',11,'FontWeight','normal','HorizontalAlignment','right');

        inpt1 = uicontrol('Parent',d,'Style','edit','Position',[183 146 68 29],'Units','pixels',...
                          'String','0.593','FontSize',11,'FontWeight','normal','HorizontalAlignment','center');

        inpt2 = uicontrol('Parent',d,'Style','edit','Position',[183 105 68 29],'Units','pixels',...
                          'String','2.100','FontSize',11,'FontWeight','normal','HorizontalAlignment','center');

        btn1  = uicontrol('Parent',d,'Style','pushbutton','Position',[96 36 136 38],'Units','pixels',...
                          'String','OK','FontSize',12,'FontWeight','normal','HorizontalAlignment','center',...
                          'Callback',{@GrabValues,inpt1,inpt2});

        uiwait(d);

        %-------------------------------------------
        function GrabValues(btn1,event,inpt1,inpt2)

            global PixelSize ZStep

            PixelSize = str2num(inpt1.String);
                if isempty(PixelSize); PixelSize = 0; end
            ZStep = str2num(inpt2.String);
                if isempty(ZStep); ZStep = 0; end

            delete(gcf)

%-----------------------------------------------------------------------------------------------
function Channel_Selector_Callback(hObject, eventdata, handles)

        handles = Update_IsoSurfaces(handles);

        obj = findall(handles.ah,'Type','Scatter');  % Find old cell markers if they exist and delete them
        delete(obj)
        handles.CellCount_Display.String = 'nCells: 0';

        % If Box is checked, recalculate cell count --------------------------
        if logical(handles.ShowCellLocations_Check.Value)
            FP = WaitToProcessDialogue; drawnow;
            handles = UpdateCellCount(handles);
            delete(FP)
        end
        %---------------------------------------------------------------------

        guidata(hObject, handles);

%-----------------------------------------------------------------------------------------------
function Channel_Selector_CreateFcn(hObject, eventdata, handles)

        if ispc && isequal(get(hObject,'BackgroundColor'), get(0,'defaultUicontrolBackgroundColor'))
            set(hObject,'BackgroundColor','white');
        end


%-----------------------------------------------------------------------------------------------
function IsoValueLow_Input_Callback(hObject, eventdata, handles)

        val = str2double(handles.IsoValueLow_Input.String);

        if ~isnan(val)
            handles.IsoLow = val;
            colormap(handles.ah, handles.ColorMap)
            caxis(handles.ah, [handles.IsoLow, handles.IsoHigh])
            handles.isoValues = round(linspace(handles.IsoHigh, handles.IsoLow, handles.nIso)');
            handles.HC.Ticks = flipud(handles.isoValues)';
            handles.HC.TickLabels = num2cell(flipud(handles.isoValues));
            handles = Update_IsoSurfaces(handles);

            obj = findall(handles.ah,'Type','Scatter');
            delete(obj)
            handles.CellCount_Display.String = 'nCells: 0';

            % If Box is checked, recalculate cell count --------------
            if logical(handles.ShowCellLocations_Check.Value)
                FP = WaitToProcessDialogue; drawnow;
                handles = UpdateCellCount(handles);
                delete(FP)
            end
            %---------------------------------------------------------
            guidata(hObject, handles);

        end
```

```
%-----------------------------------------------------------------------------------------------
function IsoValueLow_Input_CreateFcn(hObject, eventdata, handles)

        if ispc && isequal(get(hObject,'BackgroundColor'), get(0,'defaultUicontrolBackgroundColor'))
            set(hObject,'BackgroundColor','white');
        end

%-----------------------------------------------------------------------------------------------
function IsoValueHigh_Input_Callback(hObject, eventdata, handles)

        val = str2double(handles.IsoValueHigh_Input.String);

        if ~isnan(val)
            handles.IsoHigh = val;
            colormap(handles.ah, handles.ColorMap)
            caxis(handles.ah, [handles.IsoLow, handles.IsoHigh])
            handles.isoValues = round(linspace(handles.IsoHigh, handles.IsoLow, handles.nIso)');
            handles.HC.Ticks = flipud(handles.isoValues)';
            handles.HC.TickLabels = num2cell(flipud(handles.isoValues));

            handles = Update_IsoSurfaces(handles);

            guidata(hObject, handles);
        end

%---------------------------------------------------------------------------------------------------
function IsoValueHigh_Input_CreateFcn(hObject, eventdata, handles)

        if ispc && isequal(get(hObject,'BackgroundColor'), get(0,'defaultUicontrolBackgroundColor'))
            set(hObject,'BackgroundColor','white');
        end

%---------------------------------------------------------------------------------------------------
function nIsoValues_Input_Callback(hObject, eventdata, handles)

        val = str2double(handles.nIsoValues_Input.String);

        if ~isnan(val)
            handles.nIso = val;
            colormap(handles.ah, handles.ColorMap)
            caxis(handles.ah, [handles.IsoLow, handles.IsoHigh])
            handles.isoValues = round(linspace(handles.IsoHigh, handles.IsoLow, handles.nIso)');
            handles.HC.Ticks = flipud(handles.isoValues)';
            handles.HC.TickLabels = num2cell(flipud(handles.isoValues));

            handles = Update_IsoSurfaces(handles);

            guidata(hObject, handles);
        end

%----------------------------------------------------------------------------------------------------
function nIsoValues_Input_CreateFcn(hObject, eventdata, handles)

        if ispc && isequal(get(hObject,'BackgroundColor'), get(0,'defaultUicontrolBackgroundColor'))
            set(hObject,'BackgroundColor','white');
        end

%----------------------------------------------------------------------------------------------------
function ColorMaps_ListBox_Callback(hObject, eventdata, handles)

        handles.ColorMap = eval([handles.ColorMaps_ListBox.String{handles.ColorMaps_ListBox.Value},'(100)']);
        colormap(handles.ah, handles.ColorMap)
        caxis(handles.ah, [handles.IsoLow, handles.IsoHigh])
        handles.isoValues = round(linspace(handles.IsoHigh, handles.IsoLow, handles.nIso)');
        handles.HC.Ticks = flipud(handles.isoValues)';
        handles.HC.TickLabels = num2cell(flipud(handles.isoValues));

        handles = Update_IsoSurfaces(handles);

        guidata(hObject, handles);

%-----------------------------------------------------------------------------------------------------
function ColorMaps_ListBox_CreateFcn(hObject, eventdata, handles)

        if ispc && isequal(get(hObject,'BackgroundColor'), ...
                get(0,'defaultUicontrolBackgroundColor'))
            set(hObject,'BackgroundColor','white');
        end

%-----------------------------------------------------------------------------------------------------
function ShowCellLocations_Check_Callback(hObject, eventdata, handles)

        % If Box is checked, recalculate cell count --------------
        if logical(handles.ShowCellLocations_Check.Value)
            FP = WaitToProcessDialogue; drawnow;
            handles = UpdateCellCount(handles);
            delete(FP)
        else
            delete(findall(handles.ah,'Type','Scatter'))
            set(handles.CellCount_Display,'String',['nCells: 0'])
```

Development • Supplementary information

```
        end
        %---------------------------------------------------------
        guidata(hObject, handles);

%-----------------------------------------------------------------------------------------------
function ViewAngleZoom_Slider_Callback(hObject, eventdata, handles)

        val = round( get(handles.ViewAngleZoom_Slider,'Value')*10)/10;
        set(handles.ah,'CameraViewAngle',val)
        set(handles.ViewAngleZoom_Slider,'Value',val)
        set(handles.ViewZoom_Display,'String',['Angle Zoom ',sprintf('%0.1f',val)])

        guidata(hObject, handles);

%-----------------------------------------------------------------------------------------------
function ViewAngleZoom_Slider_CreateFcn(hObject, eventdata, handles)

    if isequal(get(hObject,'BackgroundColor'), get(0,'defaultUicontrolBackgroundColor'))
        set(hObject,'BackgroundColor',[.9 .9 .9]);
    end

%-----------------------------------------------------------------------------------------------
function RelativeHeight_Input_Callback(hObject, eventdata, handles)

        val = str2double(handles.RelativeHeight_Input.String);

        if ~isnan(val)
            val = min([val;200]);
            handles.RelativeHeight_Input.String = num2str(val);
        else
            handles.RelativeHeight_Input.String = '3';
        end

        % If Box is checked, recalculate cell count --------------
        if logical(handles.ShowCellLocations_Check.Value)
            FP = WaitToProcessDialogue; drawnow;
            handles = UpdateCellCount(handles);
            delete(FP)
        end
        %---------------------------------------------------------
        guidata(hObject, handles);

%-----------------------------------------------------------------------------------------------
function RelativeHeight_Input_CreateFcn(hObject, eventdata, handles)

    if ispc && isequal(get(hObject,'BackgroundColor'), get(0,'defaultUicontrolBackgroundColor'))
        set(hObject,'BackgroundColor','white');
    end

%-----------------------------------------------------------------------------------------------
function DeleteEdgeMarkers_Check_Callback(hObject, eventdata, handles)


%-----------------------------------------------------------------------------------------------
function Suggested_Z_Sigma_Button_Callback(hObject, eventdata, handles)

    xySigma = str2double(handles.XYSigmaGaussFilt_Input.String);
    zSigma  = xySigma/handles.dZdXRatio;
    handles.ZSigmaGaussFilt_Input.String = sprintf('%0.2f', zSigma);
    nP = num2str(2*ceil(2*xySigma)+1);
    nZ = num2str(2*ceil(2*zSigma )+1);
    set(handles.FilterSize_Display,'String',['Filter Size:   ',nP,' x ',nP,' x ',nZ])

    guidata(hObject, handles);

%-----------------------------------------------------------------------------------------------
function ShowYZCrossSection_Button_Callback(hObject, eventdata, handles)

     % Create New YZ Cross-Section ----------------------------------------------
         handles = Update_YZ_Cross_Section(handles);
     %--------------------------------------------------------------------------

     guidata(hObject, handles)

%-----------------------------------------------------------------------------------------------
function ExportMarkers_Button_Callback(hObject, eventdata, handles)

    [FileName,PathName] = uiputfile('*.tif');

    if ~isequal(FileName,0)
        FilePath = fullfile(PathName,FileName);
        XYZ = round(handles.CellCountLocationsVoxels);
        XYZ(XYZ==0) = 1;
        XYZ = sortrows(XYZ,3);
        se = strel('disk',1);

        for z = 1:handles.ImageStackSize(1,3)
            idx = find(XYZ(:,3) == z)';
            ZSlice = zeros(handles.ImageStackSize(1,1:2),'uint8');
            for p = idx
```

Development • Supplementary information

```
                R = XYZ(p,2);
                C = XYZ(p,1);
                ZSlice(R,C) = 255;
            end
            ZSlice = imdilate(ZSlice,se);
            if z == 1
                imwrite(ZSlice,FilePath,'tif','WriteMode','overwrite','Compression','none')
            else
                imwrite(ZSlice,FilePath,'tif','WriteMode','append','Compression','none')
            end
        end
    end

%-------------------------------------------------------------------------------------------
function ShowIsoSurfaces_Check_Callback(hObject, eventdata, handles)

    handles = Update_IsoSurfaces(handles);

    guidata(hObject, handles)

%-------------------------------------------------------------------------------------------
function SmallStepSize_ListBox_Callback(hObject, eventdata, handles)

    V = handles.SmallStepSize_ListBox.Value;
    Range = handles.X_Axis_Vector(end)-handles.ViewWidth;
    BigStep = handles.ViewWidth/Range;

    switch V
        case 1
                SmallStep = 0.5/Range;
        case 2
                SmallStep = 1/Range;
        case 3
                SmallStep = 0.1*BigStep;
    end

    set(handles.X_Position_Slider,'SliderStep',[SmallStep,BigStep])

    guidata(hObject, handles)

%-------------------------------------------------------------------------------------------
function SmallStepSize_ListBox_CreateFcn(hObject, eventdata, handles)

    if ispc && isequal(get(hObject,'BackgroundColor'), get(0,'defaultUicontrolBackgroundColor'))
        set(hObject,'BackgroundColor','white');
    end


% --- Executes on button press in About_Button.
function About_Button_Callback(hObject, eventdata, handles)

   message = [{' Abacus written and designed by:  Keith R. Carney'};...
               {'      '};...
               {'          email: keith.carney@path.utah.edu '};...
               {'             or:      ny2ak2ut@hotmail.com '};...
               {'      '};...
               {'      '};...
               {'     University of Utah '};...
               {'     Department of Human Genetics '};...
               {'     Kristen M. Kwan Lab, 2018 '};...
               {'      '};
               {'      '}];

    f = figure('NumberTitle','off',...
               'Position',[200 200 440 200],...
               'Name','About Abacus',...
               'Resize','off',...
               'Toolbar','none',...
               'WindowStyle','normal',...
               'MenuBar','none');

    uicontrol('Parent',f,'Style','text','FontName','FixedWidth','FontWeight','bold',...
         'FontSize',10,'String',message,'Position',[15 5 440 170],...
         'HorizontalAlignment','Left');





% --- Creates and returns a handle to the GUI figure.
function h1 = abacus_LayoutFcn(policy)
% policy - create a new figure or use a singleton. 'new' or 'reuse'.

persistent hsingleton;
if strcmpi(policy, 'reuse') & ishandle(hsingleton)
    h1 = hsingleton;
    return;
```

```
end

appdata = [];
appdata.GUIDEOptions = struct(...
    'active_h', [], ...
    'taginfo', struct(...
    'figure', 2, ...
    'text', 26, ...
    'pushbutton', 7, ...
    'uipanel', 8, ...
    'checkbox', 5, ...
    'edit', 9, ...
    'slider', 3, ...
    'popupmenu', 4), ...
    'override', 0, ...
    'release', [], ...
    'resize', 'none', ...
    'accessibility', 'callback', ...
    'mfile', [], ...
    'callbacks', [], ...
    'singleton', [], ...
    'syscolorfig', [], ...
    'blocking', 0, ...
    'lastSavedFile', 'C:\Matlab Files\Abacus\Main Program Files\test\abacus.m', ...
    'lastFilename', 'C:\Matlab Files\Abacus\Main Program Files\abacus.fig');
appdata.lastValidTag = 'figure1';
appdata.GUIDELayoutEditor = [];
appdata.initTags = struct(...
    'handle', [], ...
    'tag', 'figure1');

h1 = figure(...
'Units',get(0,'defaultfigureUnits'),...
'Position',[680 548 325 620],...
'Visible',get(0,'defaultfigureVisible'),...
'Color',get(0,'defaultfigureColor'),...
'IntegerHandle','off',...
'MenuBar','none',...
'Name','abacus',...
'NumberTitle','off',...
'Tag','figure1',...
'Resize','off',...
'PaperPosition',get(0,'defaultfigurePaperPosition'),...
'ScreenPixelsPerInchMode','manual',...
'HandleVisibility','callback',...
'CreateFcn', {@local_CreateFcn, blanks(0), appdata} );

appdata = [];
appdata.lastValidTag = 'Panel1';

h2 = uipanel(...
'Parent',h1,...
'FontUnits',get(0,'defaultuipanelFontUnits'),...
'Units','pixels',...
'BorderType','beveledin',...
'BorderWidth',2,...
'Title',blanks(0),...
'Tag','Panel1',...
'Position',[-1 424 330 196],...
'CreateFcn', {@local_CreateFcn, blanks(0), appdata} );

appdata = [];
appdata.lastValidTag = 'Select_File_Button';

h3 = uicontrol(...
'Parent',h2,...
'FontUnits',get(0,'defaultuicontrolFontUnits'),...
'Units',get(0,'defaultuicontrolUnits'),...
'String','Select File',...
'Style',get(0,'defaultuicontrolStyle'),...
'Position',[5 134 90 56],...
'Callback',@(hObject,eventdata)abacus('Select_File_Button_Callback',hObject,eventdata,guidata(hObject)),...
'Children',[],...
'Tag','Select_File_Button',...
'FontSize',10,...
'FontWeight','bold',...
'CreateFcn', {@local_CreateFcn, blanks(0), appdata} );

appdata = [];
appdata.lastValidTag = 'uipanel1';

h4 = uipanel(...
'Parent',h2,...
'FontUnits',get(0,'defaultuipanelFontUnits'),...
'Units','pixels',...
'Title',blanks(0),...
'Tag','uipanel1',...
'Position',[100 134 220 55],...
'CreateFcn', {@local_CreateFcn, blanks(0), appdata} );
```

```
appdata = [];
appdata.lastValidTag = 'ImageName_Display';

h5 = uicontrol(...
'Parent',h4,...
'FontUnits',get(0,'defaultuicontrolFontUnits'),...
'Units',get(0,'defaultuicontrolUnits'),...
'String',blanks(0),...
'Style','text',...
'Position',[5 5 209 46],...
'Children',[],...
'ForegroundColor',[0 0.450980392156863 0.741176470588235],...
'Tag','ImageName_Display',...
'FontSize',9,...
'FontWeight','bold',...
'CreateFcn', {@local_CreateFcn, blanks(0), appdata} );

appdata = [];
appdata.lastValidTag = 'Apply_Saturation_CheckBox';

h6 = uicontrol(...
'Parent',h2,...
'FontUnits',get(0,'defaultuicontrolFontUnits'),...
'Units',get(0,'defaultuicontrolUnits'),...
'HorizontalAlignment',get(0,'defaultuicontrolHorizontalAlignment'),...
'ListboxTop',get(0,'defaultuicontrolListboxTop'),...
'Max',get(0,'defaultuicontrolMax'),...
'Min',get(0,'defaultuicontrolMin'),...
'SliderStep',get(0,'defaultuicontrolSliderStep'),...
'String','Apply 1% Pixel Saturation',...
'Style','checkbox',...
'Value',get(0,'defaultuicontrolValue'),...
'Position',[12 109 165 20],...
'BackgroundColor',get(0,'defaultuicontrolBackgroundColor'),...
'Callback',@(hObject,eventdata)abacus('Apply_Saturation_CheckBox_Callback',hObject,eventdata,guidata(hObject)),...
'Children',[],...
'ForegroundColor',get(0,'defaultuicontrolForegroundColor'),...
'Enable',get(0,'defaultuicontrolEnable'),...
'TooltipString',blanks(0),...
'Visible',get(0,'defaultuicontrolVisible'),...
'ButtonDownFcn',blanks(0),...
'CreateFcn', {@local_CreateFcn, blanks(0), appdata} ,...
'DeleteFcn',blanks(0),...
'Tag','Apply_Saturation_CheckBox',...
'UserData',[],...
'KeyPressFcn',blanks(0),...
'KeyReleaseFcn',blanks(0),...
'HandleVisibility',get(0,'defaultuicontrolHandleVisibility'),...
'FontSize',9,...
'FontName',get(0,'defaultuicontrolFontName'),...
'FontAngle',get(0,'defaultuicontrolFontAngle'),...
'FontWeight',get(0,'defaultuicontrolFontWeight'));

appdata = [];
appdata.lastValidTag = 'Voxel_Dimension_Display';

h7 = uicontrol(...
'Parent',h2,...
'FontUnits',get(0,'defaultuicontrolFontUnits'),...
'Units',get(0,'defaultuicontrolUnits'),...
'HorizontalAlignment','left',...
'String',{  'pixel = 0.593 um'; 'zstep = 2.100 um' },...
'Style','text',...
'Position',[190 98 120 30],...
'Children',[],...
'Tag','Voxel_Dimension_Display',...
'FontSize',9,...
'FontName','Monospaced',...
'FontWeight','bold',...
'CreateFcn', {@local_CreateFcn, blanks(0), appdata} );

appdata = [];
appdata.lastValidTag = 'LoadAndProcessData_Button';

h8 = uicontrol(...
'Parent',h2,...
'FontUnits',get(0,'defaultuicontrolFontUnits'),...
'Units',get(0,'defaultuicontrolUnits'),...
'HorizontalAlignment',get(0,'defaultuicontrolHorizontalAlignment'),...
'ListboxTop',get(0,'defaultuicontrolListboxTop'),...
'Max',get(0,'defaultuicontrolMax'),...
'Min',get(0,'defaultuicontrolMin'),...
'SliderStep',get(0,'defaultuicontrolSliderStep'),...
'String','Load & Process',...
'Style',get(0,'defaultuicontrolStyle'),...
'Value',get(0,'defaultuicontrolValue'),...
'Position',[183 12 134 34],...
'BackgroundColor',[0 0.450980392156863 0.741176470588235],...
'Callback',@(hObject,eventdata)abacus('LoadAndProcessData_Button_Callback',hObject,eventdata,guidata(hObject)),...
'Children',[],...
```

```
'ForegroundColor',[1 1 0],...
'Enable',get(0,'defaultuicontrolEnable'),...
'TooltipString',blanks(0),...
'Visible',get(0,'defaultuicontrolVisible'),...
'ButtonDownFcn',blanks(0),...
'BusyAction','cancel',...
'CreateFcn', {@local_CreateFcn, blanks(0), appdata} ,...
'DeleteFcn',blanks(0),...
'Tag','LoadAndProcessData_Button',...
'UserData',[],...
'KeyPressFcn',blanks(0),...
'KeyReleaseFcn',blanks(0),...
'HandleVisibility',get(0,'defaultuicontrolHandleVisibility'),...
'FontSize',11,...
'FontName',get(0,'defaultuicontrolFontName'),...
'FontAngle',get(0,'defaultuicontrolFontAngle'),...
'FontWeight','bold');

appdata = [];
appdata.lastValidTag = 'text23';

h9 = uicontrol(...
'Parent',h2,...
'FontUnits',get(0,'defaultuicontrolFontUnits'),...
'Units',get(0,'defaultuicontrolUnits'),...
'HorizontalAlignment','left',...
'String','dz/dx = 3.54',...
'Style','text',...
'Position',[190 82 101 14],...
'Children',[],...
'ButtonDownFcn',blanks(0),...
'CreateFcn', {@local_CreateFcn, blanks(0), appdata} ,...
'DeleteFcn',blanks(0),...
'Tag','text23',...
'FontSize',9,...
'FontName','Monospaced',...
'FontWeight',get(0,'defaultuicontrolFontWeight'));

appdata = [];
appdata.lastValidTag = 'uipanel7';

h10 = uipanel(...
'Parent',h2,...
'FontUnits',get(0,'defaultuipanelFontUnits'),...
'Units','pixels',...
'Title','3D Gaussian Filter',...
'Tag','uipanel7',...
'Position',[10 9 165 87],...
'CreateFcn', {@local_CreateFcn, blanks(0), appdata} );

appdata = [];
appdata.lastValidTag = 'XYSigmaGaussFilt_Input';

h11 = uicontrol(...
'Parent',h10,...
'FontUnits',get(0,'defaultuicontrolFontUnits'),...
'Units',get(0,'defaultuicontrolUnits'),...
'HorizontalAlignment',get(0,'defaultuicontrolHorizontalAlignment'),...
'ListboxTop',get(0,'defaultuicontrolListboxTop'),...
'Max',get(0,'defaultuicontrolMax'),...
'Min',get(0,'defaultuicontrolMin'),...
'SliderStep',get(0,'defaultuicontrolSliderStep'),...
'String','0.5',...
'Style','edit',...
'Value',[],...
'Position',[59 28 40 23],...
'Callback',@(hObject,eventdata)abacus('XYSigmaGaussFilt_Input_Callback',hObject,eventdata,guidata(hObject)),...
'Children',[],...
'ForegroundColor',get(0,'defaultuicontrolForegroundColor'),...
'Enable',get(0,'defaultuicontrolEnable'),...
'TooltipString',blanks(0),...
'Visible',get(0,'defaultuicontrolVisible'),...
'ButtonDownFcn',blanks(0),...
'CreateFcn', {@local_CreateFcn, @(hObject,eventdata)abacus('XYSigmaGaussFilt_Input_CreateFcn',...
                                                    hObject,eventdata,guidata(hObject)), appdata} ,...
'DeleteFcn',blanks(0),...
'Tag','XYSigmaGaussFilt_Input',...
'UserData',[],...
'KeyPressFcn',blanks(0),...
'KeyReleaseFcn',blanks(0),...
'HandleVisibility',get(0,'defaultuicontrolHandleVisibility'),...
'FontSize',10,...
'FontName',get(0,'defaultuicontrolFontName'),...
'FontAngle',get(0,'defaultuicontrolFontAngle'),...
'FontWeight','bold');

appdata = [];
appdata.lastValidTag = 'ZSigmaGaussFilt_Input';

h12 = uicontrol(...
```

```
'Parent',h10,...
'FontUnits',get(0,'defaultuicontrolFontUnits'),...
'Units',get(0,'defaultuicontrolUnits'),...
'HorizontalAlignment',get(0,'defaultuicontrolHorizontalAlignment'),...
'ListboxTop',get(0,'defaultuicontrolListboxTop'),...
'Max',get(0,'defaultuicontrolMax'),...
'Min',get(0,'defaultuicontrolMin'),...
'SliderStep',get(0,'defaultuicontrolSliderStep'),...
'String','0.522',...
'Style','edit',...
'Value',[],...
'Position',[105 28 40 23],...
'Callback',@(hObject,eventdata)abacus('ZSigmaGaussFilt_Input_Callback',hObject,eventdata,guidata(hObject)),...
'Children',[],...
'ForegroundColor',get(0,'defaultuicontrolForegroundColor'),...
'Enable',get(0,'defaultuicontrolEnable'),...
'TooltipString',blanks(0),...
'Visible',get(0,'defaultuicontrolVisible'),...
'ButtonDownFcn',blanks(0),...
'CreateFcn', {@local_CreateFcn, @(hObject,eventdata)abacus('ZSigmaGaussFilt_Input_CreateFcn',...
                hObject,eventdata,guidata(hObject)), appdata} ,...
'DeleteFcn',blanks(0),...
'Tag','ZSigmaGaussFilt_Input',...
'UserData',[],...
'KeyPressFcn',blanks(0),...
'KeyReleaseFcn',blanks(0),...
'HandleVisibility',get(0,'defaultuicontrolHandleVisibility'),...
'FontSize',10,...
'FontName',get(0,'defaultuicontrolFontName'),...
'FontAngle',get(0,'defaultuicontrolFontAngle'),...
'FontWeight','bold');

appdata = [];
appdata.lastValidTag = 'text22';

h13 = uicontrol(...
'Parent',h10,...
'FontUnits',get(0,'defaultuicontrolFontUnits'),...
'Units',get(0,'defaultuicontrolUnits'),...
'HorizontalAlignment','left',...
'String','sigma:      xy           z',...
'Style','text',...
'Position',[16 54 120 14],...
'Children',[],...
'ButtonDownFcn',blanks(0),...
'CreateFcn', {@local_CreateFcn, blanks(0), appdata} ,...
'DeleteFcn',blanks(0),...
'Tag','text22',...
'FontSize',9,...
'FontWeight','bold');

appdata = [];
appdata.lastValidTag = 'Suggested_Z_Sigma_Button';

h14 = uicontrol(...
'Parent',h10,...
'FontUnits',get(0,'defaultuicontrolFontUnits'),...
'Units',get(0,'defaultuicontrolUnits'),...
'String','sig z',...
'Style',get(0,'defaultuicontrolStyle'),...
'Position',[17 28 32 24],...
'Callback',@(hObject,eventdata)abacus('Suggested_Z_Sigma_Button_Callback',hObject,eventdata,guidata(hObject)),...
'Children',[],...
'Tag','Suggested_Z_Sigma_Button',...
'CreateFcn', {@local_CreateFcn, blanks(0), appdata} );

appdata = [];
appdata.lastValidTag = 'FilterSize_Display';

h15 = uicontrol(...
'Parent',h10,...
'FontUnits',get(0,'defaultuicontrolFontUnits'),...
'Units',get(0,'defaultuicontrolUnits'),...
'HorizontalAlignment','left',...
'ListboxTop',get(0,'defaultuicontrolListboxTop'),...
'Max',get(0,'defaultuicontrolMax'),...
'Min',get(0,'defaultuicontrolMin'),...
'SliderStep',get(0,'defaultuicontrolSliderStep'),...
'String','Filter Size:   3 x 3 x 3',...
'Style','text',...
'Value',get(0,'defaultuicontrolValue'),...
'Position',[17 10 126 14],...
'BackgroundColor',get(0,'defaultuicontrolBackgroundColor'),...
'Callback',blanks(0),...
'Children',[],...
'ForegroundColor',get(0,'defaultuicontrolForegroundColor'),...
'Enable',get(0,'defaultuicontrolEnable'),...
'TooltipString',blanks(0),...
'Visible',get(0,'defaultuicontrolVisible'),...
'ButtonDownFcn',blanks(0),...
```

```
'CreateFcn', {@local_CreateFcn, blanks(0), appdata} ,...
'DeleteFcn',blanks(0),...
'Tag','FilterSize_Display',...
'UserData',[],...
'KeyPressFcn',blanks(0),...
'KeyReleaseFcn',blanks(0),...
'HandleVisibility',get(0,'defaultuicontrolHandleVisibility'),...
'FontSize',get(0,'defaultuicontrolFontSize'),...
'FontName',get(0,'defaultuicontrolFontName'),...
'FontAngle',get(0,'defaultuicontrolFontAngle'),...
'FontWeight',get(0,'defaultuicontrolFontWeight'));

appdata = [];
appdata.lastValidTag = 'About_Button';

h16 = uicontrol(...
'Parent',h2,...
'FontUnits',get(0,'defaultuicontrolFontUnits'),...
'Units',get(0,'defaultuicontrolUnits'),...
'String','About Abacus',...
'Style',get(0,'defaultuicontrolStyle'),...
'Position',[237 52 81 20],...
'Callback',@(hObject,eventdata)abacus('About_Button_Callback',hObject,eventdata,guidata(hObject)),...
'Children',[],...
'Tag','About_Button',...
'FontSize',7.5,...
'CreateFcn', {@local_CreateFcn, blanks(0), appdata} );

appdata = [];
appdata.lastValidTag = 'Panel2';

h17 = uipanel(...
'Parent',h1,...
'FontUnits',get(0,'defaultuipanelFontUnits'),...
'Units','pixels',...
'BorderType','beveledin',...
'BorderWidth',2,...
'Title',blanks(0),...
'Tag','Panel2',...
'Position',[-4 149 330 262],...
'CreateFcn', {@local_CreateFcn, blanks(0), appdata} );

appdata = [];
appdata.lastValidTag = 'X_Position_Slider';

h18 = uicontrol(...
'Parent',h17,...
'FontUnits',get(0,'defaultuicontrolFontUnits'),...
'Units',get(0,'defaultuicontrolUnits'),...
'String',blanks(0),...
'Style','slider',...
'Position',[10 179 305 42],...
'BackgroundColor',[0.9 0.9 0.9],...
'Callback',@(hObject,eventdata)abacus('X_Position_Slider_Callback',hObject,eventdata,guidata(hObject)),...
'Children',[],...
'BusyAction','cancel',...
'CreateFcn', {@local_CreateFcn, @(hObject,eventdata)abacus('X_Position_Slider_CreateFcn',...
                                hObject,eventdata,guidata(hObject)), appdata} ,...
'Tag','X_Position_Slider');

appdata = [];
appdata.lastValidTag = 'Width_Input';

h19 = uicontrol(...
'Parent',h17,...
'FontUnits',get(0,'defaultuicontrolFontUnits'),...
'Units',get(0,'defaultuicontrolUnits'),...
'String','40',...
'Style','edit',...
'Position',[252 227 35 22],...
'Callback',@(hObject,eventdata)abacus('Width_Input_Callback',hObject,eventdata,guidata(hObject)),...
'Children',[],...
'CreateFcn', {@local_CreateFcn, @(hObject,eventdata)abacus('Width_Input_CreateFcn',...
                                   hObject,eventdata,guidata(hObject)), appdata} ,...
'Tag','Width_Input',...
'FontSize',9);

appdata = [];
appdata.lastValidTag = 'text7';

h20 = uicontrol(...
'Parent',h17,...
'FontUnits',get(0,'defaultuicontrolFontUnits'),...
'Units',get(0,'defaultuicontrolUnits'),...
'HorizontalAlignment','left',...
'String','um',...
'Style','text',...
'Position',[289 230 25 15],...
'Children',[],...
'CreateFcn', {@local_CreateFcn, blanks(0), appdata} ,...
```

Development • Supplementary information

```
'Tag','text7',...
'FontSize',9);

appdata = [];
appdata.lastValidTag = 'text8';

h21 = uicontrol(...
'Parent',h17,...
'FontUnits',get(0,'defaultuicontrolFontUnits'),...
'Units',get(0,'defaultuicontrolUnits'),...
'HorizontalAlignment','right',...
'String','View Width:',...
'Style','text',...
'Position',[177 230 73 15],...
'Children',[],...
'ButtonDownFcn',blanks(0),...
'CreateFcn', {@local_CreateFcn, blanks(0), appdata} ,...
'DeleteFcn',blanks(0),...
'Tag','text8',...
'FontSize',9);

appdata = [];
appdata.lastValidTag = 'SliderBarPosition_Display';

h22 = uicontrol(...
'Parent',h17,...
'FontUnits',get(0,'defaultuicontrolFontUnits'),...
'Units',get(0,'defaultuicontrolUnits'),...
'HorizontalAlignment','left',...
'String','X Position: 150 um',...
'Style','text',...
'Position',[20 233 151 15],...
'Children',[],...
'ButtonDownFcn',blanks(0),...
'CreateFcn', {@local_CreateFcn, blanks(0), appdata} ,...
'DeleteFcn',blanks(0),...
'Tag','SliderBarPosition_Display',...
'FontSize',11,...
'FontWeight','bold');

appdata = [];
appdata.lastValidTag = 'PositionSliderMinValue_Display';

h23 = uicontrol(...
'Parent',h17,...
'FontUnits',get(0,'defaultuicontrolFontUnits'),...
'Units',get(0,'defaultuicontrolUnits'),...
'HorizontalAlignment','left',...
'String','20',...
'Style','text',...
'Position',[11 159 42 18],...
'Children',[],...
'CreateFcn', {@local_CreateFcn, blanks(0), appdata} ,...
'Tag','PositionSliderMinValue_Display',...
'FontSize',10);

appdata = [];
appdata.lastValidTag = 'PositionSliderMaxValue_Display';

h24 = uicontrol(...
'Parent',h17,...
'FontUnits',get(0,'defaultuicontrolFontUnits'),...
'Units',get(0,'defaultuicontrolUnits'),...
'HorizontalAlignment','right',...
'String','0',...
'Style','text',...
'Position',[260 159 55 18],...
'Children',[],...
'ButtonDownFcn',blanks(0),...
'CreateFcn', {@local_CreateFcn, blanks(0), appdata} ,...
'DeleteFcn',blanks(0),...
'Tag','PositionSliderMaxValue_Display',...
'FontSize',10);

appdata = [];
appdata.lastValidTag = 'Channel_Selector';

h25 = uicontrol(...
'Parent',h17,...
'FontUnits',get(0,'defaultuicontrolFontUnits'),...
'Units',get(0,'defaultuicontrolUnits'),...
'String',{  'Ch 1'; 'Ch 2'; 'Ch 3' },...
'Style','popupmenu',...
'Value',[],...
'ValueMode',get(0,'defaultuicontrolValueMode'),...
'Position',[11 98 75 24],...
'Callback',@(hObject,eventdata)abacus('Channel_Selector_Callback',hObject,eventdata,guidata(hObject)),...
'Children',[],...
'BusyAction','cancel',...
'CreateFcn', {@local_CreateFcn, @(hObject,eventdata)abacus('Channel_Selector_CreateFcn',...
```

```
                                    hObject,eventdata,guidata(hObject)), appdata} ,...
'Tag','Channel_Selector',...
'FontSize',10,...
'FontWeight','bold');

appdata = [];
appdata.lastValidTag = 'uipanel5';

h26 = uipanel(...
'Parent',h17,...
'FontUnits',get(0,'defaultuipanelFontUnits'),...
'Units','pixels',...
'ForegroundColor',[0 0.450980392156863 0.741176470588235],...
'Title','IsoSurface Parameters',...
'Tag','uipanel5',...
'Position',[92 33 226 95],...
'CreateFcn', {@local_CreateFcn, blanks(0), appdata} );

appdata = [];
appdata.lastValidTag = 'IsoValueLow_Input';

h27 = uicontrol(...
'Parent',h26,...
'FontUnits',get(0,'defaultuicontrolFontUnits'),...
'Units',get(0,'defaultuicontrolUnits'),...
'String','90',...
'Style','edit',...
'Position',[143 12 35 22],...
'Callback',@(hObject,eventdata)abacus('IsoValueLow_Input_Callback',hObject,eventdata,guidata(hObject)),...
'Children',[],...
'ButtonDownFcn',blanks(0),...
'BusyAction','cancel',...
'CreateFcn', {@local_CreateFcn, @(hObject,eventdata)abacus('IsoValueLow_Input_CreateFcn',...
                                         hObject,eventdata,guidata(hObject)), appdata} ,...
'DeleteFcn',blanks(0),...
'Tag','IsoValueLow_Input',...
'KeyPressFcn',blanks(0),...
'FontSize',9);

appdata = [];
appdata.lastValidTag = 'text14';

h28 = uicontrol(...
'Parent',h26,...
'FontUnits',get(0,'defaultuicontrolFontUnits'),...
'Units',get(0,'defaultuicontrolUnits'),...
'HorizontalAlignment',get(0,'defaultuicontrolHorizontalAlignment'),...
'String','Low',...
'Style','text',...
'Position',[144 35 33 15],...
'Children',[],...
'ForegroundColor',[0 0.450980392156863 0.741176470588235],...
'ButtonDownFcn',blanks(0),...
'CreateFcn', {@local_CreateFcn, blanks(0), appdata} ,...
'DeleteFcn',blanks(0),...
'Tag','text14',...
'FontSize',get(0,'defaultuicontrolFontSize'));

appdata = [];
appdata.lastValidTag = 'IsoValueHigh_Input';

h29 = uicontrol(...
'Parent',h26,...
'FontUnits',get(0,'defaultuicontrolFontUnits'),...
'Units',get(0,'defaultuicontrolUnits'),...
'String','200',...
'Style','edit',...
'Position',[181 12 35 22],...
'Callback',@(hObject,eventdata)abacus('IsoValueHigh_Input_Callback',hObject,eventdata,guidata(hObject)),...
'Children',[],...
'ButtonDownFcn',blanks(0),...
'BusyAction','cancel',...
'CreateFcn', {@local_CreateFcn, @(hObject,eventdata)abacus('IsoValueHigh_Input_CreateFcn',...
                                         hObject,eventdata,guidata(hObject)), appdata} ,...
'DeleteFcn',blanks(0),...
'Tag','IsoValueHigh_Input',...
'KeyPressFcn',blanks(0),...
'FontSize',9);

appdata = [];
appdata.lastValidTag = 'text15';

h30 = uicontrol(...
'Parent',h26,...
'FontUnits',get(0,'defaultuicontrolFontUnits'),...
'Units',get(0,'defaultuicontrolUnits'),...
'HorizontalAlignment',get(0,'defaultuicontrolHorizontalAlignment'),...
'String','High',...
'Style','text',...
'Position',[182 35 33 15],...
```

```
'Children',[],...
'ForegroundColor',[0 0.450980392156863 0.741176470588235],...
'ButtonDownFcn',blanks(0),...
'CreateFcn', {@local_CreateFcn, blanks(0), appdata} ,...
'DeleteFcn',blanks(0),...
'Tag','text15',...
'FontSize',get(0,'defaultuicontrolFontSize'));

appdata = [];
appdata.lastValidTag = 'text16';

h31 = uicontrol(...
'Parent',h26,...
'FontUnits',get(0,'defaultuicontrolFontUnits'),...
'Units',get(0,'defaultuicontrolUnits'),...
'HorizontalAlignment',get(0,'defaultuicontrolHorizontalAlignment'),...
'String','N',...
'Style','text',...
'Position',[109 35 21 15],...
'Children',[],...
'ForegroundColor',[0 0.450980392156863 0.741176470588235],...
'ButtonDownFcn',blanks(0),...
'CreateFcn', {@local_CreateFcn, blanks(0), appdata} ,...
'DeleteFcn',blanks(0),...
'Tag','text16',...
'FontSize',get(0,'defaultuicontrolFontSize'));

appdata = [];
appdata.lastValidTag = 'nIsoValues_Input';

h32 = uicontrol(...
'Parent',h26,...
'FontUnits',get(0,'defaultuicontrolFontUnits'),...
'Units',get(0,'defaultuicontrolUnits'),...
'String','3',...
'Style','edit',...
'Position',[106 12 28 22],...
'Callback',@(hObject,eventdata)abacus('nIsoValues_Input_Callback',hObject,eventdata,guidata(hObject)),...
'Children',[],...
'ButtonDownFcn',blanks(0),...
'BusyAction','cancel',...
'CreateFcn', {@local_CreateFcn, @(hObject,eventdata)abacus('nIsoValues_Input_CreateFcn',...
                                            hObject,eventdata,guidata(hObject)), appdata} ,...
'DeleteFcn',blanks(0),...
'Tag','nIsoValues_Input',...
'KeyPressFcn',blanks(0),...
'FontSize',9);

appdata = [];
appdata.lastValidTag = 'ColorMaps_ListBox';

h33 = uicontrol(...
'Parent',h26,...
'FontUnits',get(0,'defaultuicontrolFontUnits'),...
'Units',get(0,'defaultuicontrolUnits'),...
'String',blanks(0),...
'Style','popupmenu',...
'Value',[],...
'ValueMode',get(0,'defaultuicontrolValueMode'),...
'Position',[11 12 85 22],...
'Callback',@(hObject,eventdata)abacus('ColorMaps_ListBox_Callback',hObject,eventdata,guidata(hObject)),...
'Children',[],...
'BusyAction','cancel',...
'CreateFcn', {@local_CreateFcn, @(hObject,eventdata)abacus('ColorMaps_ListBox_CreateFcn',...
                                           hObject,eventdata,guidata(hObject)), appdata} ,...
'Tag','ColorMaps_ListBox',...
'FontSize',9);

appdata = [];
appdata.lastValidTag = 'text18';

h34 = uicontrol(...
'Parent',h26,...
'FontUnits',get(0,'defaultuicontrolFontUnits'),...
'Units',get(0,'defaultuicontrolUnits'),...
'HorizontalAlignment',get(0,'defaultuicontrolHorizontalAlignment'),...
'String','Color Map',...
'Style','text',...
'Position',[8 35 58 15],...
'Children',[],...
'ForegroundColor',[0 0.450980392156863 0.741176470588235],...
'ButtonDownFcn',blanks(0),...
'CreateFcn', {@local_CreateFcn, blanks(0), appdata} ,...
'DeleteFcn',blanks(0),...
'Tag','text18',...
'FontSize',get(0,'defaultuicontrolFontSize'));

appdata = [];
appdata.lastValidTag = 'ShowIsoSurfaces_Check';
```

```
h35 = uicontrol(...
'Parent',h26,...
'FontUnits',get(0,'defaultuicontrolFontUnits'),...
'Units',get(0,'defaultuicontrolUnits'),...
'String','Show IsoSurfaces',...
'Style','checkbox',...
'Position',[12 57 115 20],...
'Callback',@(hObject,eventdata)abacus('ShowIsoSurfaces_Check_Callback',hObject,eventdata,guidata(hObject)),...
'Children',[],...
'ButtonDownFcn',blanks(0),...
'CreateFcn', {@local_CreateFcn, blanks(0), appdata} ,...
'DeleteFcn',blanks(0),...
'Tag','ShowIsoSurfaces_Check',...
'KeyPressFcn',blanks(0));

appdata = [];
appdata.lastValidTag = 'ViewAngleZoom_Slider';

h36 = uicontrol(...
'Parent',h17,...
'FontUnits',get(0,'defaultuicontrolFontUnits'),...
'Units',get(0,'defaultuicontrolUnits'),...
'String',{  'Slider' },...
'Style','slider',...
'Position',[10 56 75 23],...
'BackgroundColor',[0.9 0.9 0.9],...
'Callback',@(hObject,eventdata)abacus('ViewAngleZoom_Slider_Callback',hObject,eventdata,guidata(hObject)),...
'Children',[],...
'BusyAction','cancel',...
'CreateFcn', {@local_CreateFcn, @(hObject,eventdata)abacus('ViewAngleZoom_Slider_CreateFcn',...
                                           hObject,eventdata,guidata(hObject)), appdata} ,...
'Tag','ViewAngleZoom_Slider');

appdata = [];
appdata.lastValidTag = 'ViewZoom_Display';

h37 = uicontrol(...
'Parent',h17,...
'FontUnits',get(0,'defaultuicontrolFontUnits'),...
'Units',get(0,'defaultuicontrolUnits'),...
'HorizontalAlignment','left',...
'ListboxTop',get(0,'defaultuicontrolListboxTop'),...
'Max',get(0,'defaultuicontrolMax'),...
'Min',get(0,'defaultuicontrolMin'),...
'SliderStep',get(0,'defaultuicontrolSliderStep'),...
'String','Angle Zoom: 10',...
'Style','text',...
'Value',get(0,'defaultuicontrolValue'),...
'Position',[9 35 80 14],...
'BackgroundColor',get(0,'defaultuicontrolBackgroundColor'),...
'Callback',blanks(0),...
'Children',[],...
'ForegroundColor',get(0,'defaultuicontrolForegroundColor'),...
'Enable',get(0,'defaultuicontrolEnable'),...
'TooltipString',blanks(0),...
'Visible',get(0,'defaultuicontrolVisible'),...
'ButtonDownFcn',blanks(0),...
'CreateFcn', {@local_CreateFcn, blanks(0), appdata} ,...
'DeleteFcn',blanks(0),...
'Tag','ViewZoom_Display',...
'UserData',[],...
'KeyPressFcn',blanks(0),...
'KeyReleaseFcn',blanks(0),...
'HandleVisibility',get(0,'defaultuicontrolHandleVisibility'),...
'FontSize',get(0,'defaultuicontrolFontSize'),...
'FontName',get(0,'defaultuicontrolFontName'),...
'FontAngle',get(0,'defaultuicontrolFontAngle'),...
'FontWeight',get(0,'defaultuicontrolFontWeight'));

appdata = [];
appdata.lastValidTag = 'ShowYZCrossSection_Button';

h38 = uicontrol(...
'Parent',h17,...
'FontUnits',get(0,'defaultuicontrolFontUnits'),...
'Units',get(0,'defaultuicontrolUnits'),...
'String','Show YZ Cross Section',...
'Style','checkbox',...
'Position',[176 9 140 20],...
'Callback',@(hObject,eventdata)abacus('ShowYZCrossSection_Button_Callback',hObject,eventdata,guidata(hObject)),...
'Children',[],...
'CreateFcn', {@local_CreateFcn, blanks(0), appdata} ,...
'Tag','ShowYZCrossSection_Button');

appdata = [];
appdata.lastValidTag = 'SmallStepSize_ListBox';

h39 = uicontrol(...
'Parent',h17,...
'FontUnits',get(0,'defaultuicontrolFontUnits'),...
```

```
'Units',get(0,'defaultuicontrolUnits'),...
'String','1/10 of View Width',...
'Style','popupmenu',...
'Value',[],...
'ValueMode',get(0,'defaultuicontrolValueMode'),...
'Position',[141 146 124 22],...
'Callback',@(hObject,eventdata)abacus('SmallStepSize_ListBox_Callback',hObject,eventdata,guidata(hObject)),...
'Children',[],...
'CreateFcn', {@local_CreateFcn, @(hObject,eventdata)abacus('SmallStepSize_ListBox_CreateFcn',...
                                       hObject,eventdata,guidata(hObject)), appdata} ,...
'Tag','SmallStepSize_ListBox');

appdata = [];
appdata.lastValidTag = 'text25';

h40 = uicontrol(...
'Parent',h17,...
'FontUnits',get(0,'defaultuicontrolFontUnits'),...
'Units',get(0,'defaultuicontrolUnits'),...
'String','Slider Small Step:',...
'Style','text',...
'Position',[51 149 87 14],...
'Children',[],...
'Tag','text25',...
'CreateFcn', {@local_CreateFcn, blanks(0), appdata} );

appdata = [];
appdata.lastValidTag = 'Panel3';

h41 = uipanel(...
'Parent',h1,...
'FontUnits',get(0,'defaultuipanelFontUnits'),...
'Units','pixels',...
'BorderType','beveledin',...
'BorderWidth',2,...
'Title',blanks(0),...
'Tag','Panel3',...
'Position',[-4 18 330 116],...
'CreateFcn', {@local_CreateFcn, blanks(0), appdata} );

appdata = [];
appdata.lastValidTag = 'ShowCellLocations_Check';

h42 = uicontrol(...
'Parent',h41,...
'FontUnits',get(0,'defaultuicontrolFontUnits'),...
'Units',get(0,'defaultuicontrolUnits'),...
'String','Calculate Cell Count',...
'Style','checkbox',...
'Position',[11 85 191 23],...
'Callback',@(hObject,eventdata)abacus('ShowCellLocations_Check_Callback',hObject,eventdata,guidata(hObject)),...
'Children',[],...
'CreateFcn', {@local_CreateFcn, blanks(0), appdata} ,...
'Tag','ShowCellLocations_Check',...
'FontSize',12,...
'FontWeight','bold');

appdata = [];
appdata.lastValidTag = 'CellCount_Display';

h43 = uicontrol(...
'Parent',h41,...
'FontUnits',get(0,'defaultuicontrolFontUnits'),...
'Units',get(0,'defaultuicontrolUnits'),...
'HorizontalAlignment','left',...
'String','nCells: 0',...
'Style','text',...
'Position',[9 16 142 22],...
'Children',[],...
'ForegroundColor',[0.63921568627451 0.0784313725490196 0.180392156862745],...
'Tag','CellCount_Display',...
'FontSize',14,...
'FontWeight','bold',...
'CreateFcn', {@local_CreateFcn, blanks(0), appdata} );

appdata = [];
appdata.lastValidTag = 'text21';

h44 = uicontrol(...
'Parent',h41,...
'FontUnits',get(0,'defaultuicontrolFontUnits'),...
'Units',get(0,'defaultuicontrolUnits'),...
'HorizontalAlignment','right',...
'String','ImExtendedMax Relative Height:',...
'Style','text',...
'Position',[23 46 160 14],...
'Children',[],...
'Tag','text21',...
'FontSize',get(0,'defaultuicontrolFontSize'),...
'CreateFcn', {@local_CreateFcn, blanks(0), appdata} );
```

```
appdata = [];
appdata.lastValidTag = 'RelativeHeight_Input';

h45 = uicontrol(...
'Parent',h41,...
'FontUnits',get(0,'defaultuicontrolFontUnits'),...
'Units',get(0,'defaultuicontrolUnits'),...
'String','10',...
'Style','edit',...
'Position',[184 44 25 18],...
'Callback',@(hObject,eventdata)abacus('RelativeHeight_Input_Callback',hObject,eventdata,guidata(hObject)),...
'Children',[],...
'CreateFcn', {@local_CreateFcn, @(hObject,eventdata)abacus('RelativeHeight_Input_CreateFcn',...
                                         hObject,eventdata,guidata(hObject)), appdata} ,...
'Tag','RelativeHeight_Input',...
'FontSize',get(0,'defaultuicontrolFontSize'));

appdata = [];
appdata.lastValidTag = 'DeleteEdgeMarkers_Check';

h46 = uicontrol(...
'Parent',h41,...
'FontUnits',get(0,'defaultuicontrolFontUnits'),...
'Units',get(0,'defaultuicontrolUnits'),...
'String','Delete Edge Markers',...
'Style','checkbox',...
'Position',[30 61 131 23],...
'Callback',@(hObject,eventdata)abacus('DeleteEdgeMarkers_Check_Callback',hObject,eventdata,guidata(hObject)),...
'Children',[],...
'ButtonDownFcn',blanks(0),...
'CreateFcn', {@local_CreateFcn, blanks(0), appdata} ,...
'DeleteFcn',blanks(0),...
'Tag','DeleteEdgeMarkers_Check',...
'KeyPressFcn',blanks(0),...
'FontSize',get(0,'defaultuicontrolFontSize'));

appdata = [];
appdata.lastValidTag = 'ExportMarkers_Button';

h47 = uicontrol(...
'Parent',h41,...
'FontUnits',get(0,'defaultuicontrolFontUnits'),...
'Units',get(0,'defaultuicontrolUnits'),...
'String','Export Markers',...
'Style',get(0,'defaultuicontrolStyle'),...
'Position',[228 12 96 30],...
'Callback',@(hObject,eventdata)abacus('ExportMarkers_Button_Callback',hObject,eventdata,guidata(hObject)),...
'Children',[],...
'Tag','ExportMarkers_Button',...
'CreateFcn', {@local_CreateFcn, blanks(0), appdata} );


hsingleton = h1;


% --- Set application data first then calling the CreateFcn.
function local_CreateFcn(hObject, eventdata, createfcn, appdata)

if ~isempty(appdata)
   names = fieldnames(appdata);
   for i=1:length(names)
       name = char(names(i));
       setappdata(hObject, name, getfield(appdata,name));
   end
end

if ~isempty(createfcn)
   if isa(createfcn,'function_handle')
       createfcn(hObject, eventdata);
   else
       eval(createfcn);
   end
end


% --- Handles default GUIDE GUI creation and callback dispatch
function varargout = gui_mainfcn(gui_State, varargin)

gui_StateFields =  {'gui_Name'
    'gui_Singleton'
    'gui_OpeningFcn'
    'gui_OutputFcn'
    'gui_LayoutFcn'
    'gui_Callback'};
gui_Mfile = '';
for i=1:length(gui_StateFields)
    if ~isfield(gui_State, gui_StateFields{i})
        error(message('MATLAB:guide:StateFieldNotFound', gui_StateFields{ i }, gui_Mfile));
    elseif isequal(gui_StateFields{i}, 'gui_Name')
```

```
        gui_Mfile = [gui_State.(gui_StateFields{i}), '.m'];
    end
end

numargin = length(varargin);

if numargin == 0
    % ABACUS
    % create the GUI only if we are not in the process of loading it
    % already
    gui_Create = true;
elseif local_isInvokeActiveXCallback(gui_State, varargin{:})
    % ABACUS(ACTIVEX,...)
    vin{1} = gui_State.gui_Name;
    vin{2} = [get(varargin{1}.Peer, 'Tag'), '_', varargin{end}];
    vin{3} = varargin{1};
    vin{4} = varargin{end-1};
    vin{5} = guidata(varargin{1}.Peer);
    feval(vin{:});
    return;
elseif local_isInvokeHGCallback(gui_State, varargin{:})
    % ABACUS('CALLBACK',hObject,eventData,handles,...)
    gui_Create = false;
else
    % ABACUS(...)
    % create the GUI and hand varargin to the openingfcn
    gui_Create = true;
end

if ~gui_Create
    % In design time, we need to mark all components possibly created in
    % the coming callback evaluation as non-serializable. This way, they
    % will not be brought into GUIDE and not be saved in the figure file
    % when running/saving the GUI from GUIDE.
    designEval = false;
    if (numargin>1 && ishghandle(varargin{2}))
        fig = varargin{2};
        while ~isempty(fig) && ~ishghandle(fig,'figure')
            fig = get(fig,'parent');
        end

        designEval = isappdata(0,'CreatingGUIDEFigure') || (isscalar(fig)&&isprop(fig,'GUIDEFigure'));
    end

    if designEval
        beforeChildren = findall(fig);
    end

    % evaluate the callback now
    varargin{1} = gui_State.gui_Callback;
    if nargout
        [varargout{1:nargout}] = feval(varargin{:});
    else
        feval(varargin{:});
    end

    % Set serializable of objects created in the above callback to off in
    % design time. Need to check whether figure handle is still valid in
    % case the figure is deleted during the callback dispatching.
    if designEval && ishghandle(fig)
        set(setdiff(findall(fig),beforeChildren), 'Serializable','off');
    end
else
    if gui_State.gui_Singleton
        gui_SingletonOpt = 'reuse';
    else
        gui_SingletonOpt = 'new';
    end

    % Check user passing 'visible' P/V pair first so that its value can be
    % used by oepnfig to prevent flickering
    gui_Visible = 'auto';
    gui_VisibleInput = '';
    for index=1:2:length(varargin)
        if length(varargin) == index || ~ischar(varargin{index})
            break;
        end

        % Recognize 'visible' P/V pair
        len1 = min(length('visible'),length(varargin{index}));
        len2 = min(length('off'),length(varargin{index+1}));
        if ischar(varargin{index+1}) && strncmpi(varargin{index},'visible',len1) && len2 > 1
            if strncmpi(varargin{index+1},'off',len2)
                gui_Visible = 'invisible';
                gui_VisibleInput = 'off';
            elseif strncmpi(varargin{index+1},'on',len2)
                gui_Visible = 'visible';
                gui_VisibleInput = 'on';
            end
        end
```

Development • Supplementary information

```
end

% Open fig file with stored settings.  Note: This executes all component
% specific CreateFunctions with an empty HANDLES structure.


% Do feval on layout code in m-file if it exists
gui_Exported = ~isempty(gui_State.gui_LayoutFcn);
% this application data is used to indicate the running mode of a GUIDE
% GUI to distinguish it from the design mode of the GUI in GUIDE. it is
% only used by actxproxy at this time.
setappdata(0,genvarname(['OpenGuiWhenRunning_', gui_State.gui_Name]),1);
if gui_Exported
    gui_hFigure = feval(gui_State.gui_LayoutFcn, gui_SingletonOpt);

    % make figure invisible here so that the visibility of figure is
    % consistent in OpeningFcn in the exported GUI case
    if isempty(gui_VisibleInput)
        gui_VisibleInput = get(gui_hFigure,'Visible');
    end
    set(gui_hFigure,'Visible','off')

    % openfig (called by local_openfig below) does this for guis without
    % the LayoutFcn. Be sure to do it here so guis show up on screen.
    movegui(gui_hFigure,'onscreen');
else
    gui_hFigure = local_openfig(gui_State.gui_Name, gui_SingletonOpt, gui_Visible);
    % If the figure has InGUIInitialization it was not completely created
    % on the last pass.  Delete this handle and try again.
    if isappdata(gui_hFigure, 'InGUIInitialization')
        delete(gui_hFigure);
        gui_hFigure = local_openfig(gui_State.gui_Name, gui_SingletonOpt, gui_Visible);
    end
end
if isappdata(0, genvarname(['OpenGuiWhenRunning_', gui_State.gui_Name]))
    rmappdata(0,genvarname(['OpenGuiWhenRunning_', gui_State.gui_Name]));
end

% Set flag to indicate starting GUI initialization
setappdata(gui_hFigure,'InGUIInitialization',1);

% Fetch GUIDE Application options
gui_Options = getappdata(gui_hFigure,'GUIDEOptions');
% Singleton setting in the GUI MATLAB code file takes priority if different
gui_Options.singleton = gui_State.gui_Singleton;

if ~isappdata(gui_hFigure,'GUIOnScreen')
    % Adjust background color
    if gui_Options.syscolorfig
        set(gui_hFigure,'Color', get(0,'DefaultUicontrolBackgroundColor'));
    end

    % Generate HANDLES structure and store with GUIDATA. If there is
    % user set GUI data already, keep that also.
    data = guidata(gui_hFigure);
    handles = guihandles(gui_hFigure);
    if ~isempty(handles)
        if isempty(data)
            data = handles;
        else
            names = fieldnames(handles);
            for k=1:length(names)
                data.(char(names(k)))=handles.(char(names(k)));
            end
        end
    end
    guidata(gui_hFigure, data);
end

% Apply input P/V pairs other than 'visible'
for index=1:2:length(varargin)
    if length(varargin) == index || ~ischar(varargin{index})
        break;
    end

    len1 = min(length('visible'),length(varargin{index}));
    if ~strncmpi(varargin{index},'visible',len1)
        try set(gui_hFigure, varargin{index}, varargin{index+1}), catch break, end
    end
end

% If handle visibility is set to 'callback', turn it on until finished
% with OpeningFcn
gui_HandleVisibility = get(gui_hFigure,'HandleVisibility');
if strcmp(gui_HandleVisibility, 'callback')
    set(gui_hFigure,'HandleVisibility', 'on');
end

feval(gui_State.gui_OpeningFcn, gui_hFigure, [], guidata(gui_hFigure), varargin{:});
```

Development • Supplementary information

```
    if isscalar(gui_hFigure) && ishghandle(gui_hFigure)
        % Handle the default callbacks of predefined toolbar tools in this
        % GUI, if any
        guidemfile('restoreToolbarToolPredefinedCallback',gui_hFigure);

        % Update handle visibility
        set(gui_hFigure,'HandleVisibility', gui_HandleVisibility);

        % Call openfig again to pick up the saved visibility or apply the
        % one passed in from the P/V pairs
        if ~gui_Exported
            gui_hFigure = local_openfig(gui_State.gui_Name, 'reuse',gui_Visible);
        elseif ~isempty(gui_VisibleInput)
            set(gui_hFigure,'Visible',gui_VisibleInput);
        end
        if strcmpi(get(gui_hFigure, 'Visible'), 'on')
            figure(gui_hFigure);

            if gui_Options.singleton
                setappdata(gui_hFigure,'GUIOnScreen', 1);
            end
        end

        % Done with GUI initialization
        if isappdata(gui_hFigure,'InGUIInitialization')
            rmappdata(gui_hFigure,'InGUIInitialization');
        end

        % If handle visibility is set to 'callback', turn it on until
        % finished with OutputFcn
        gui_HandleVisibility = get(gui_hFigure,'HandleVisibility');
        if strcmp(gui_HandleVisibility, 'callback')
            set(gui_hFigure,'HandleVisibility', 'on');
        end
        gui_Handles = guidata(gui_hFigure);
    else
        gui_Handles = [];
    end

    if nargout
        [varargout{1:nargout}] = feval(gui_State.gui_OutputFcn, gui_hFigure, [], gui_Handles);
    else
        feval(gui_State.gui_OutputFcn, gui_hFigure, [], gui_Handles);
    end

    if isscalar(gui_hFigure) && ishghandle(gui_hFigure)
        set(gui_hFigure,'HandleVisibility', gui_HandleVisibility);
    end
end

function gui_hFigure = local_openfig(name, singleton, visible)

% openfig with three arguments was new from R13. Try to call that first, if
% failed, try the old openfig.
if nargin('openfig') == 2
    % OPENFIG did not accept 3rd input argument until R13,
    % toggle default figure visible to prevent the figure
    % from showing up too soon.
    gui_OldDefaultVisible = get(0,'defaultFigureVisible');
    set(0,'defaultFigureVisible','off');
    gui_hFigure = matlab.hg.internal.openfigLegacy(name, singleton);
    set(0,'defaultFigureVisible',gui_OldDefaultVisible);
else
    % Call version of openfig that accepts 'auto' option"
    gui_hFigure = matlab.hg.internal.openfigLegacy(name, singleton, visible);
%     %workaround for CreateFcn not called to create ActiveX
%         peers=findobj(findall(allchild(gui_hFigure)),'type','uicontrol','style','text');
%         for i=1:length(peers)
%             if isappdata(peers(i),'Control')
%                 actxproxy(peers(i));
%             end
%         end
end

function result = local_isInvokeActiveXCallback(gui_State, varargin)

try
    result = ispc && iscom(varargin{1}) ...
             && isequal(varargin{1},gcbo);
catch
    result = false;
end

function result = local_isInvokeHGCallback(gui_State, varargin)

try
    fhandle = functions(gui_State.gui_Callback);
    result = ~isempty(findstr(gui_State.gui_Name,fhandle.file)) || ...
             (ischar(varargin{1}) ...
             && isequal(ishghandle(varargin{2}), 1) ...
```

Development • Supplementary information

```
            && (~isempty(strfind(varargin{1},[get(varargin{2}, 'Tag'), '_'])) || ...
               ~isempty(strfind(varargin{1}, '_CreateFcn'))) );
catch
    result = false;
end
```
